# Metabolites profiling and pharmacokinetics of troxipide and its pharmacodynamics in rats with gastric ulcer

**DOI:** 10.1038/s41598-020-70312-7

**Published:** 2020-08-12

**Authors:** Hongbin Guo, Baohua Chen, Zihan Yan, Jian Gao, Jiamei Tang, Chengyan Zhou

**Affiliations:** College of Pharmaceutical Sciences, Institute of Life Science and Green Development, Key Laboratory of Pharmaceutical Quality Control of Hebei Province, Hebei University, 180 WuSi Road, Lianchi District, Baoding, 071002 China

**Keywords:** Chemical biology, Molecular biology, Medical research, Molecular medicine

## Abstract

Troxipide is widely used to treat gastric ulcer (GU) in the clinic. However, a lack of systematic metabolic, pharmacokinetic and pharmacological studies limits its clinical use. This study aimed to firstly explore the metabolic, pharmacokinetic and pharmacological mechanisms of troxipide in rats with GU compared to normal control (NC) rats. First, metabolic study was perormed by a highly selective, high-resolution mass spectrometry method. A total of 45 metabolites, including 9 phase I metabolites and 36 phase II metabolites, were identified based on MS/MS spectra. Subsequently, the pharmacokinetics results suggested that the C_max_, K_a_, t_1/2_, AUC_(0−t)_ and AUC_(0−∞)_ of troxipide were significantly increased in rats with GU compared with NC rats. The V_z_, K_10_ and absolute bioavailability of troxipide were obviously decreased in rats with GU compared with NC rats, and its tissue distribution (in the liver, lung and kidney) was significantly different between the two groups of rats. Additionally, the pharmacodynamic results suggested that the levels of biochemical factors (IL-17, IL-6, TNF-α, IFN-γ, AP-1, MTL, GAS, and PG-II) were significantly increased, the PG-Ӏ level was obviously decreased, and the protein expression levels of HSP-90, C-Cas-3 and C-PARP-1 were markedly increased in rats with GU compared with NC rats. The above results suggested that the therapeutic mechanisms underlying the metabolic, pharmacokinetic and pharmacological properties of troxipide in vivo in rats deserve further attention based on the importance of troxipide in the treatment of GU in this study, and these mechanisms could be targets for future studies.

## Introduction

Gastric ulcer, (GU) a common disease in the clinic, is a peptic ulcer that affects humans of all ages. It is associated with morbidity and mortality, and has become a medical-social problem of global importance that has received increased attention^1–4^. The frequent occurrence of GU between the cardia and pylorus mainly results form tissue damage caused by digestive juices decomposing the gastric mucosa, especially near the lesser curvature of the stomach and gastric antrum^5–8^. Currently, GU is a widely considered multifactorial disease because it is related to gastric acid, gastric pepsin, infection, physical fitness, environment, living habits and so on^9–11^. If not treated adequately, GU can lead to serious complications such as perforation and bleeding^12–15^. In addition, the acetic acid-induced GU in rats is most similar to GU in humans^16–20^. Here, acetic acid-induced GU is more severe than other GU models (ethanol-, ligation- and reserpine-induced models), and is the most commonly used model for experimental research on GU^21,22^. Moreover,this model is reproducible and reliable. Therefore, we selected a rat model of GU to evaluate relative studies of troxipide in the paper.

**Figure 1:**
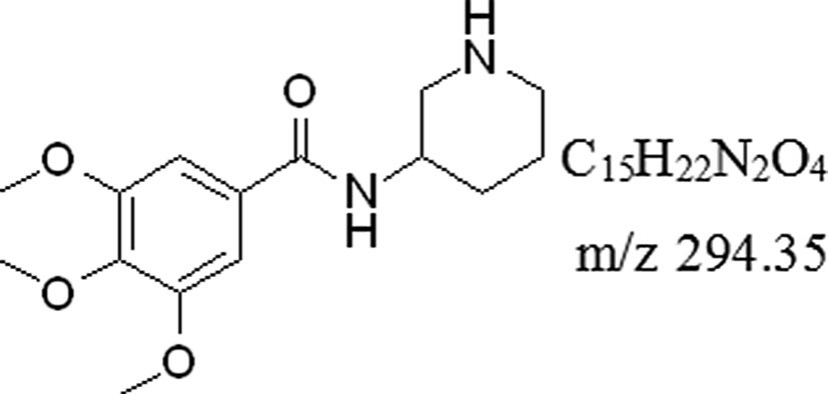
The formula of troxipide.

Troxipide (3,4,5-trimethoxy-N-3-piperidinyl, Fig. 1), a defensive factor-enhancing therapeutic agent for gastritis and GU that exerts inhibitory, therapeutic and preventive effects to specifically those in the stomach by enhancing gastric mucosal blood flow and gastric mucosal defense factors and promoting tissue repair, blood circulation, metabolism and GU repair^23–25^. It is used to alleviate gastric mucosal lesions (erosion, hemorrhage, redness, and edema) in the acute gastritis and acute exacerbation stages of chronic gastritis. Furthermore, troxipide can inhibit neutrophil-mediated inflammation and oxidative stress to increase gastric mucus secretion, increase the contents of mucopolysaccharide and prostaglandin E2 and enhance the positive effect of the gastric mucosa on barrier function^26–28^. And, the troxipide is soluble only at acidic pH and is mainly absorbed from the stomach^29^. However, few researchers have payed attention to the pharmacokinetic and metabolic process of troxipide. So, detailed studies on the metabolites, pharmacokinetics and therapeutic mechanisms of troxipide in rats with GU are lacking, limiting the clinical use of this drug.

Unsurprisingly, drug metabolism research in association with drug discovery and development has increased significantly. For many years, research on drug metabolism has been of utmost importance for drug research, development and application; the pharmacokinetic and toxicological properties of drugs confirm the importance of drug metabolism research and serve as drivers for further research^30^. Meanwhile, drug metabolism also plays an important role in optimizing pharmacokinetics, pharmacodynamics, and safety properties, which could help researchers understand the major soft spots to the potential metabolism liability of a drug and optimize lead compounds for further development. The drug metabolism studies are requisite at different stages and these data are helpful to understand the routes of drug elimination as well as safety profiles in humans. In addition, drugs should be subjected to an array of biotransformation reactions, which can be accompanied by various effects, such as pharmacological or toxicological activities^31^. The body commonly uses these biotransformation reactions as a major line of defense against drugs^32^. Because of the biotransformation reactions impact on the fate of drugs as a whole, such as drug absorption, distribution, metabolism, excretion and toxicity. Therefore, in vitro and in vivo metabolic, pharmacokinetic and pharmacodynamic studies are essential parts of the drug discovery and development processes^33^.

**Figure 2:**
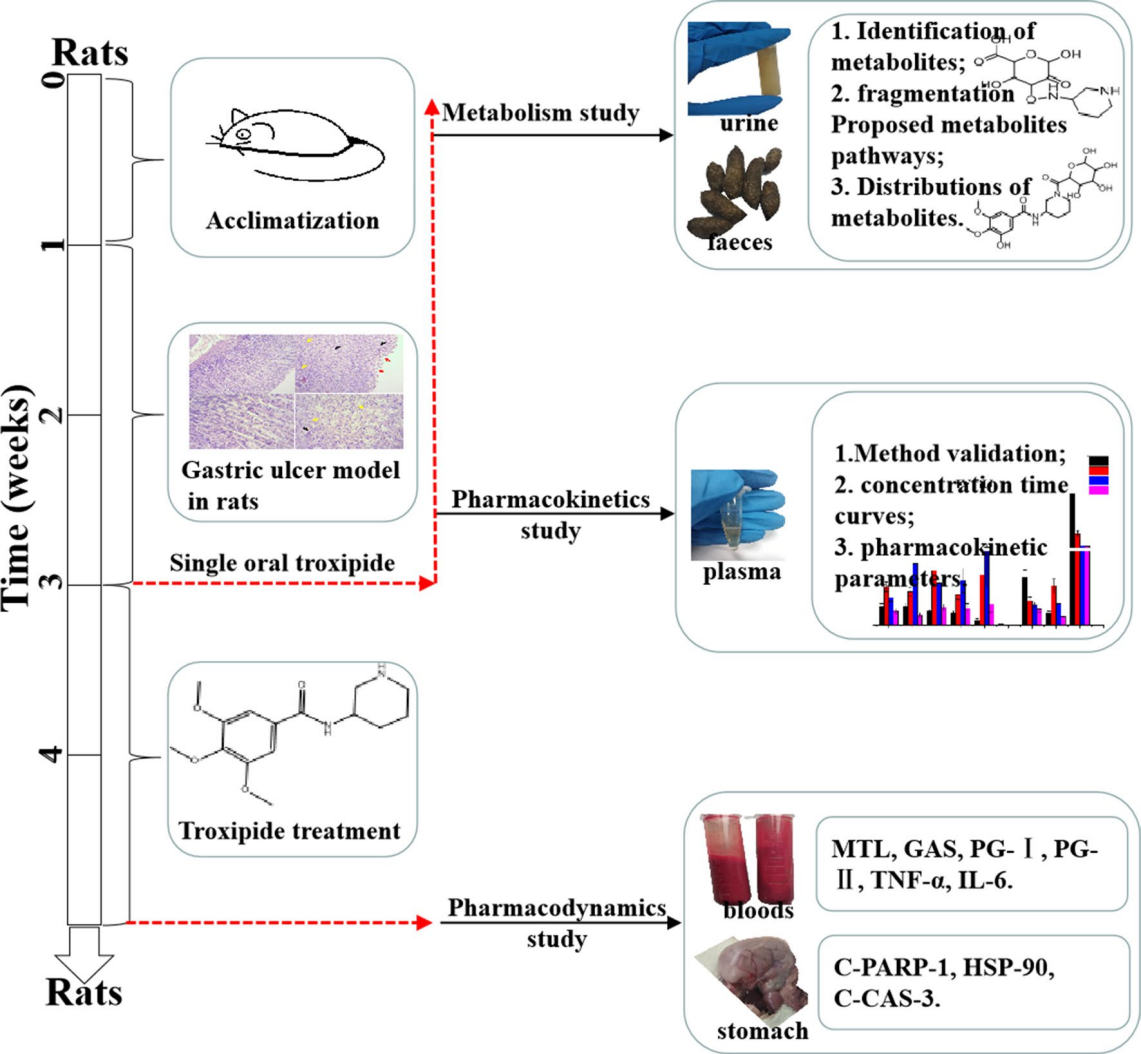
Workflow of the troxipide study.

For further understanding of troxipide, we systematically studied the metabolic and pharmacokinetic of troxipide in rats with GU and normal control (NC) rats in the present study for further research and development of troxipide in the clinic. To systematically investigate the major metabolites of troxipide in rats with acetic acid-induced GU, we first time evaluated metabolites in the feces and urine of rats with GU after oral administration of troxipide using the highly selective and high-resolution method of ultra-high-performance liquid chromatography quadrupole orbitrap mass spectrometry (UHPLC-Q-Orbitrap-MS). Then, we performed ultra-high-performance liquid chromatography-tandem mass spectrometry (UHPLC-MS/MS) to study the pharmacokinetics of troxipide in vivo in rats with carbamazepine as an internal standard (IS). This assay has some merits, such as precise sample preparation, good linearity and specificity and negligible carryover. To obtain a systematic view of dissection of the plasma UHPLC-MS-based pharmacokinetics of troxipide, the above method was firstly applied to the pharmacokinetic study of troxipide in vivo in the present study as an effective evaluation strategy for absorption and metabolism. In addition, we have also investigated the expression of relative apoptosis protein on pharmacodynamics in the paper. Overall, our study on the metabolic, pharmacokinetic and pharmacological effects of troxipide provide a foundation for the structural modification and dosage form design of troxipide. The outline of the workflow of this study was shown in Fig. 2.

## Results and discussion

### Metabolic study

#### Fragmentation behaviors of troxipide

**Figure 3:**
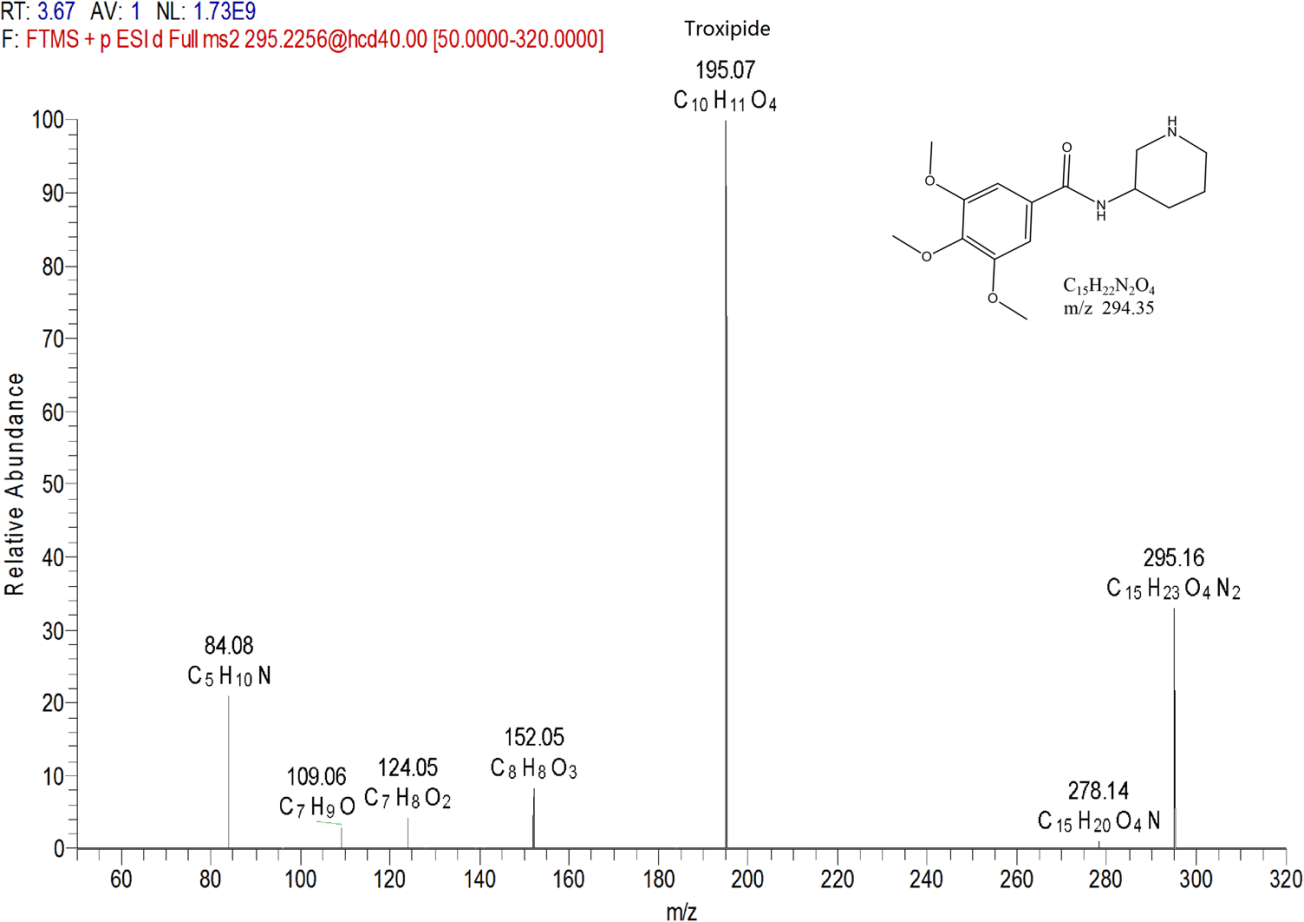
The product spectra, fragmentation reaction and structure of troxipide in positive electrospray ionization mode.

Drug metabolism can produce metabolites with altered functional parameters such as clearance rate, absorption rate, biological activity and toxicity; thus, it is an opportunity to improve the odds of drug discovery^32^. Because the metabolites exhibited similar structures to that of troxipide and because the biologically active conformation of troxipide was optimized during the biotransformation process, the metabolites may have a wide range of pharmacological activities^34^. To study the structure of troxipide metabolites, Compound Discovery software, Mass Frontier software and manual elucidation were used to evaluate the fragmentation behaviors of troxipide metabolites. As shown in Fig. 3, troxipide (C_15_H_23_N_2_O_4_) was protonated [M+H]^+^ with m/z 295.16 in the ESI–MS spectrum. In the ESI-MS^2^ spectra, the base peak at m/z 195.07 yielded an [M + H-C_5_H_12_N_2_]^+^ ion through the loss of 3-aminopiperidine. The molecular ion at m/z 295.16 also produced the product ion at m/z 84.08, which was a piperidine ring resulting from the loss of C_10_H_13_O_4_N. Moreover, minor ions, including the [295.16-NH3]^+^ ion at m/z 278.14, [295.16-C_5_H_12_N_2_-C_2_H_3_O]^+^ ion at m/z 152.05, [295.16-C_5_H_12_N_2_-C_2_H_3_O-CO]^+^ ion at m/z 124.05, and [295.16-C_5_H_12_N_2_-C_2_H_3_O–C_3_H_2_O_3_]^+^ ion at m/z 109.06, were also observed in the ESI-MS^2^ spectrum. According to the fragmentation pattern of the parent compound, the two fragmentation pathways of troxipide are shown in Fig. S1. The main pathway involved two steps: amide bond cleavage and piperidine separation. The more clear the fragmentation of troxipide, the easier it was to identify the metabolites. The fragmentation pathways mentioned above could be adopted to precisely diagnose the structural skeletons and substitution patterns of metabolites in the complex matrices.

#### Identification of troxipide metabolites

**Figure 4:**
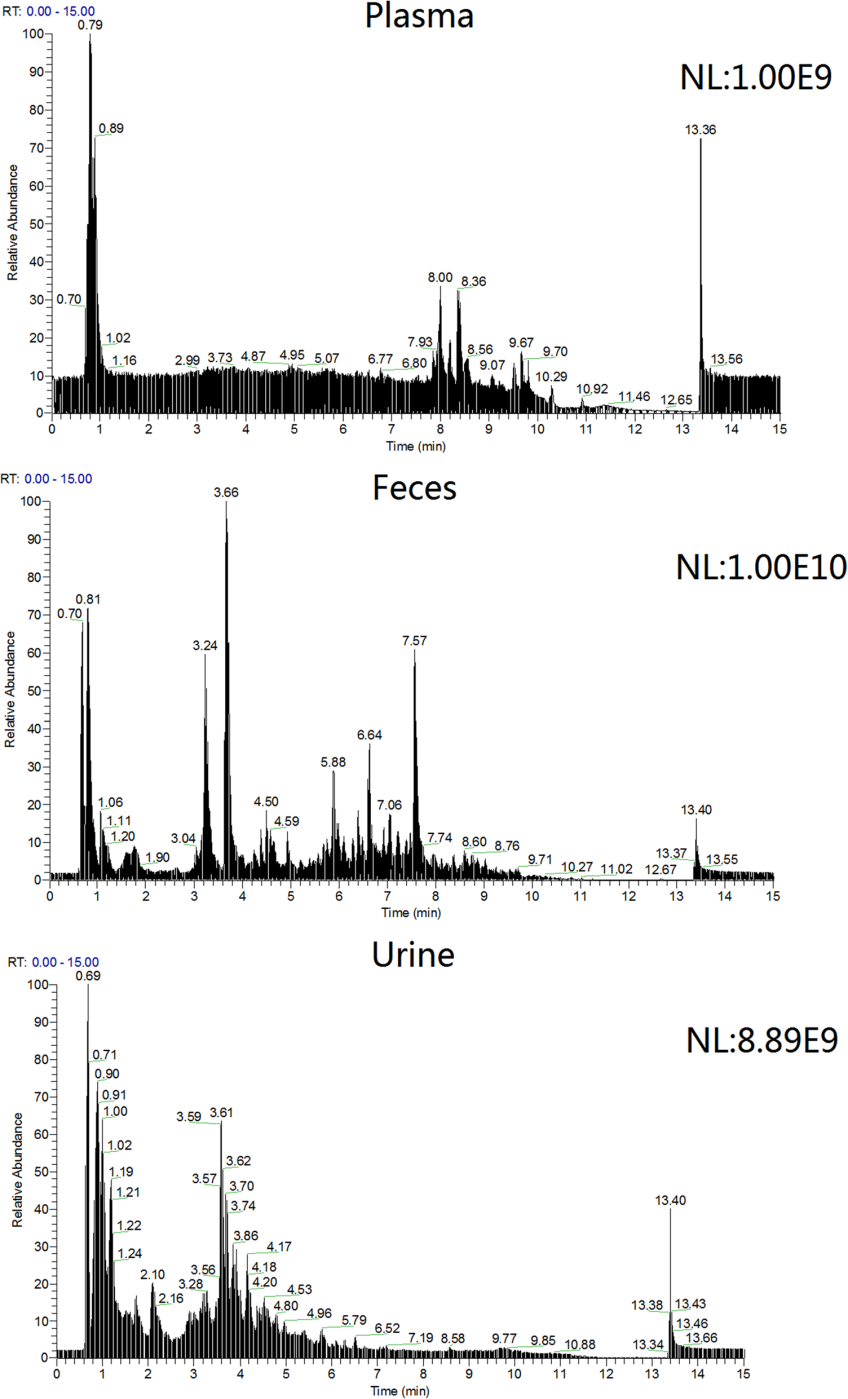
The TIC spectra of the main metabolites of troxipide in rat plasma, fecal and urine samples.

The main metabolite total ion current (TIC) spectra of troxipide in rat plasma, feces and urine samples are shown Fig. 4. The data were processed using Compound Discovery software (generic peak finding, multiple mass defect filtering, isotope pattern matching, finding metabolites based on common product ions or neutral losses, searched Mass Lists, searched mzcloud and ChemSpider)^35^. Many possible metabolites and metabolic pathways were identified by this software by automatically comparing a sample collected after drug administration and a blank sample. Information on the metabolites, such as the formula, m/z, retention time (RT), peak area, and peak height, was provided by the software. In addition, this software provided some metabolic pathways and MS/MS fragments to determine accurate masses of different parts of troxipide for metabolite structural identification. This method shown some features such as shorter run time, sample preparation simplicity and so on, which has been successfully applied for detecting metabolites products of troxipide and could be practical and reliable for clinical, pharmacodynamics and does-dependent pharmacokinetic study of troxipide and its active metabolite.

Approximately 10,000 metabolites related to precursor ions were identified in the plasma, urine and feces of rats with GU compared with control rat plasma, urine and feces, and more than 2000 metabolites were screened by peak area filtering. Finally, in positive full scan mode, 45 metabolites from the urine and feces were successfully detected and verified by the fragmentation library of Mass Frontier software. The structures of the metabolites were determined based on precise mass measurements, drug biotransformation knowledge, and characteristic fragmentation pathways. The main biochemical reactions were oxidation, reduction, demethylation, glucuronide conjugation, palmitoyl conjugation and so on. For our knowledge, the 45 metabolites was reported for the first time, the LC–MS/MS spectra of these metabolites were showed in Fig. S2 and the basic information was listed in Table 1.

**Table 1:**
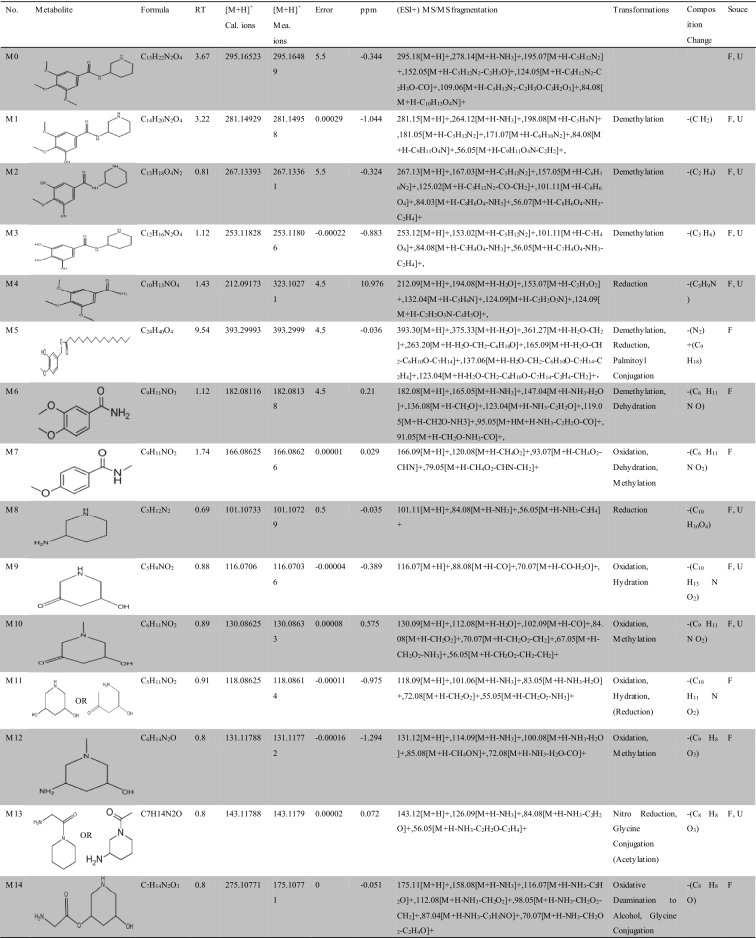

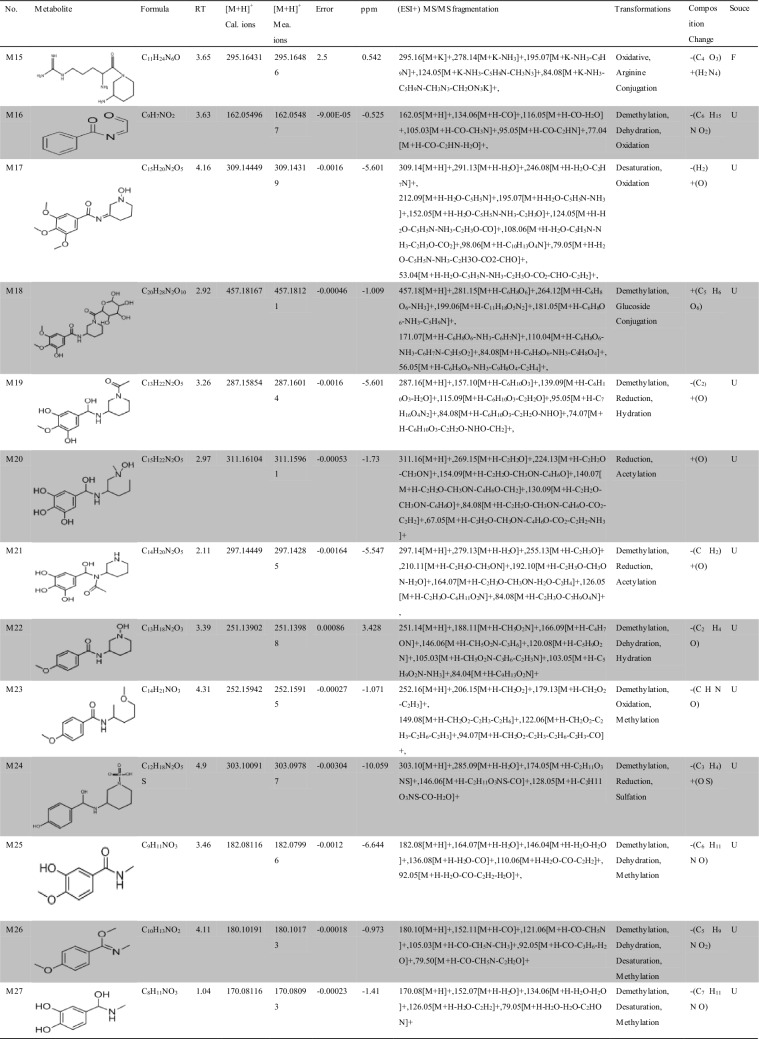

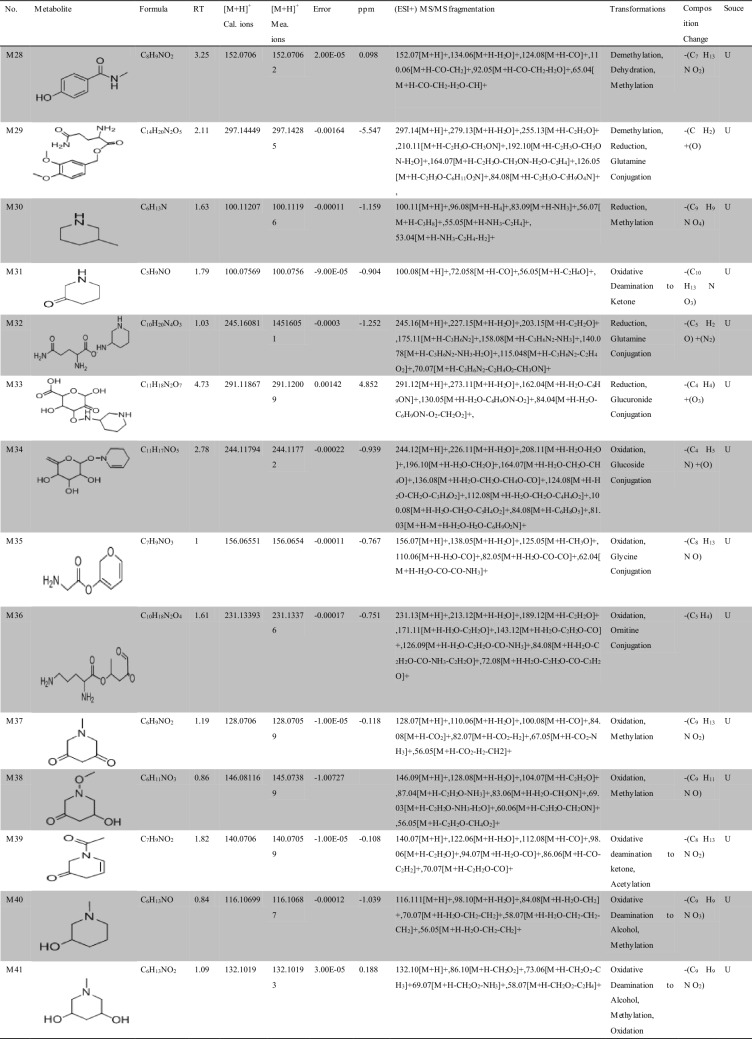

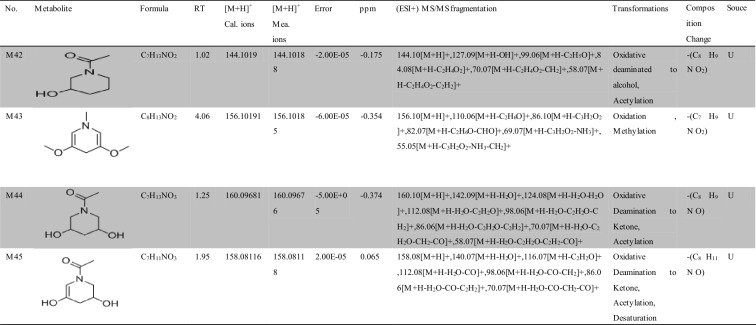
Troxipide metabolites detected and structurally characterized on a UHPLC-Q-Orbitrap-MS. U, urine sample; F, fecal sample; P, plasma sample.

**Figure 5:**
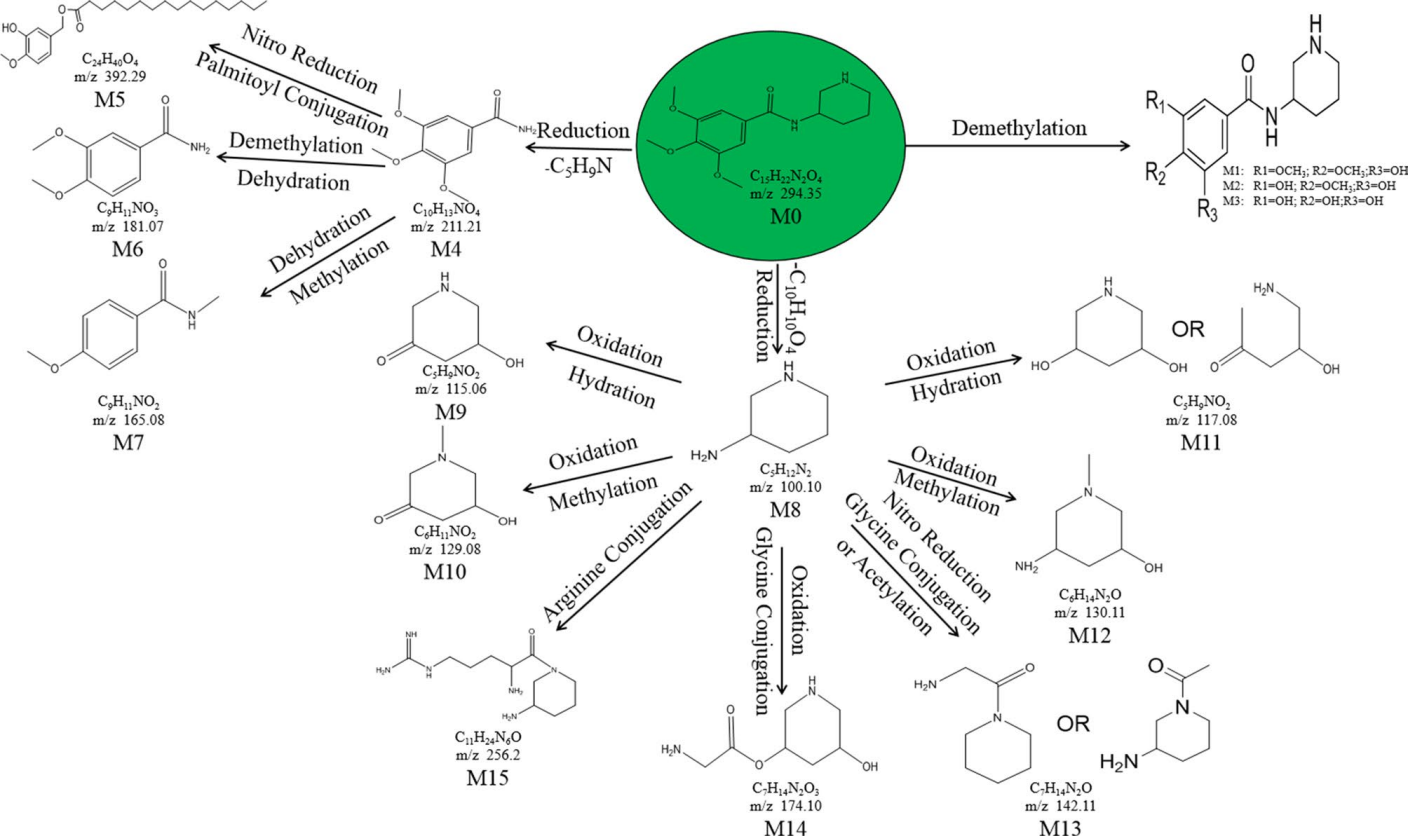
Proposed metabolic pathway of troxipide in the feces of rats with GU.

**Figure 6:**
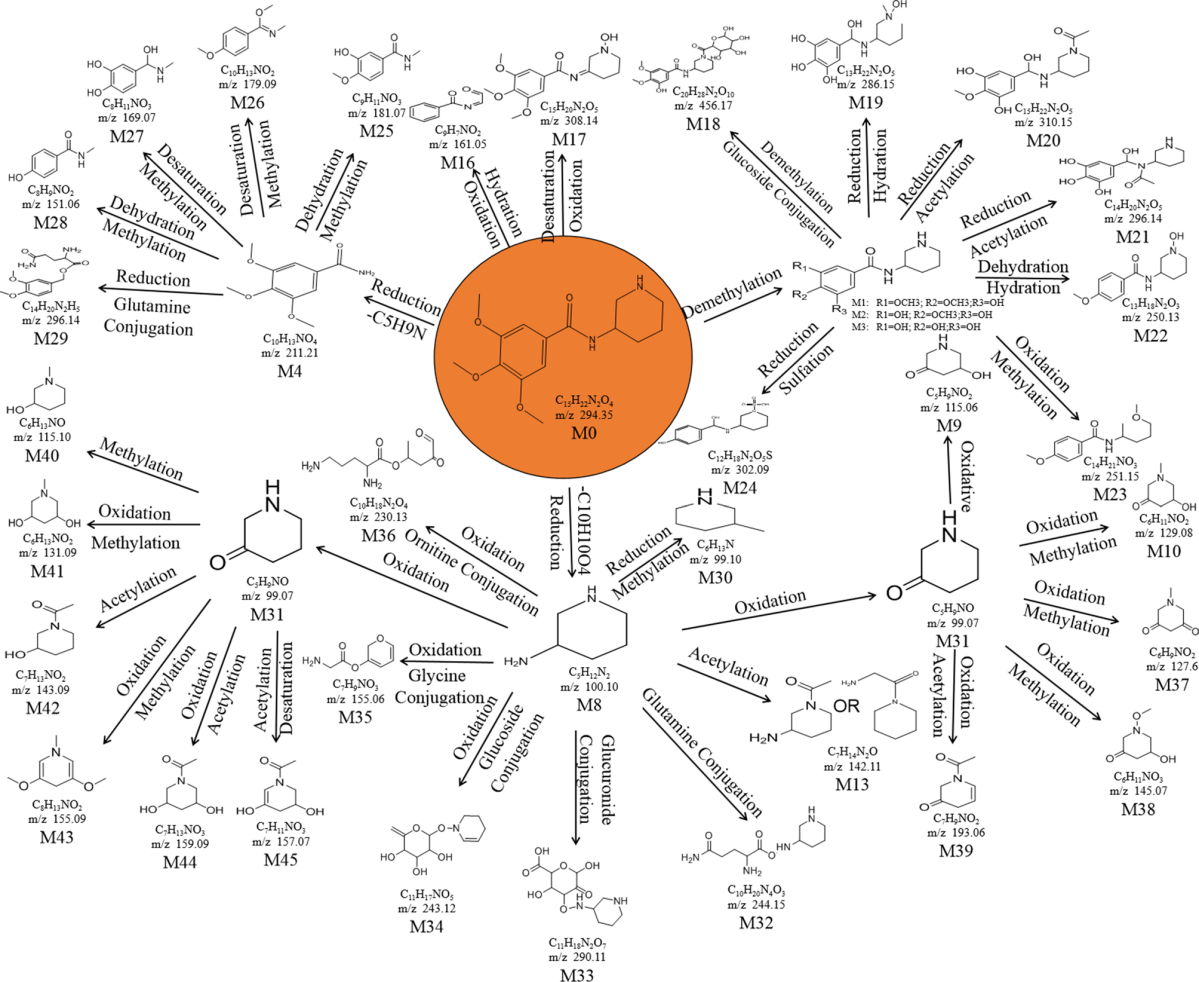
Proposed metabolic pathway of troxipide in the urine of rats with GU.

Here, we also showed the identification process on the following key metabolites of troxipide. Metabolites 4 and 8 (M4 and M8) were derivatives of the parent drug and its fragmentation and were detected and identified in the urine and feces of the rats. The results are shown in Figs. 5 and 6. The metabolite M0 was eluted at 3.67 min, with an protonated [M + H]^+^ molecular ion at m/z 295.16, and the product ions were at m/z 195.07 and 84.08, which was consistent with the results for troxipide. Therefore, M0 was confirmed to be troxipide. The [M + H]^+^ molecular ion of M4, which exhibited a mass that was 84 Da less than that of M0, was detected at m/z 211.21 at 1.43 min. The characteristic MS/MS ions of M4 were at m/z 132.05 and 153.07. Based on the difference between its composition and that of the parent drug, M4 was determined to be a metabolite that was generated from M0 through the loss of pyridine. The [M + H]^+^ molecular ion of M8, which had a RT of 0.69 min, was detected at m/z 100.10, and the fragment ions were at m/z 84.08 and 56.05. According to its elemental composition and degree of unsaturation, M8 was surmised to be a metabolite resulting from amide bond cleavage of M0, as detailed in Supplementary Results and Discussion.

The proposed major metabolic pathways were present in the feces and urine of the rats, and small fractions of the compounds were distributed in the blood. In the feces, troxipide mainly underwent demethylation, dehydration, hydration, reduction, oxidation, methylation, acetylation, palmitoyl conjugation, glycine conjugation, glycine conjugation, and arginine conjugation. These findings may be related to the intestinal flora in rats with GU^36,37^. But, the metabolic reactions of troxipide in feces weren’t all agreement with that in urine. In the urine, troxipide mainly underwent demethylation, dehydration, desaturation, hydration, reduction, oxidation, methylation, acetylation, sulfation conjugation, glutamine conjugation, ornithine conjugation, glucoside conjugation, glutamine conjugation, glycine conjugation, and glucuronide conjugation. There results may be related to liver metabolism^38,39^. Specially, oxidative metabolism is the most common mechanism of drug metabolism and involves a series of biochemical reactions that produce more readily excretable species. Much importance is given to cytochromes P450 (CYPs) in drug metabolism, and some medicinal chemists have prematurely suggested that CYPs are the only significant enzymes in drug metabolism. The actions of CYPs dominate those of other oxidoreductases and account for approximately half of metabolite formation. Hydrolysis may produce approximately 1/10 of all metabolites, and the remaining reactions are attributed to conjugating enzymes. Glucuronosyltransferases are the most important enzymes, providing structural variety and allowing the formation of functional groups to which glucuronic acid can be conjugated, while sulfotransferases and glutathione S-transferases, methyltransferases, N-acetyltransferases, and other coenzyme-A transferases play lesser roles^40^. On the above, different metabolites of troxipide may result from different metabolic pathways associating with oxidoreductases, hydrolytic enzyme and conjugating enzymes in the study. In addition, the blood was not a good matrix for monitoring exposure to troxipide metabolites in this study. Therefore, the findings of this study regarding troxipide metabolites in the blood have not done the further investigation.

### Pharmacokinetics and tissue distribution study

#### Method validation

The method met the pertinent Food and Drug Administration (FDA) and European Medicine Agency (EMA) guidelines, indicating that the proposed analytical method is applicable for the pharmacokinetic study of troxipide. Furthermore, the results showed that this method can significantly improve the response of troxipide. This study is original and practical and will therefore provide a good foundation for further research on troxipide and its drug development in the future, as detailed in Supplementary Results and Discussion.

#### Pharmacokinetics study

**Figure 7:**
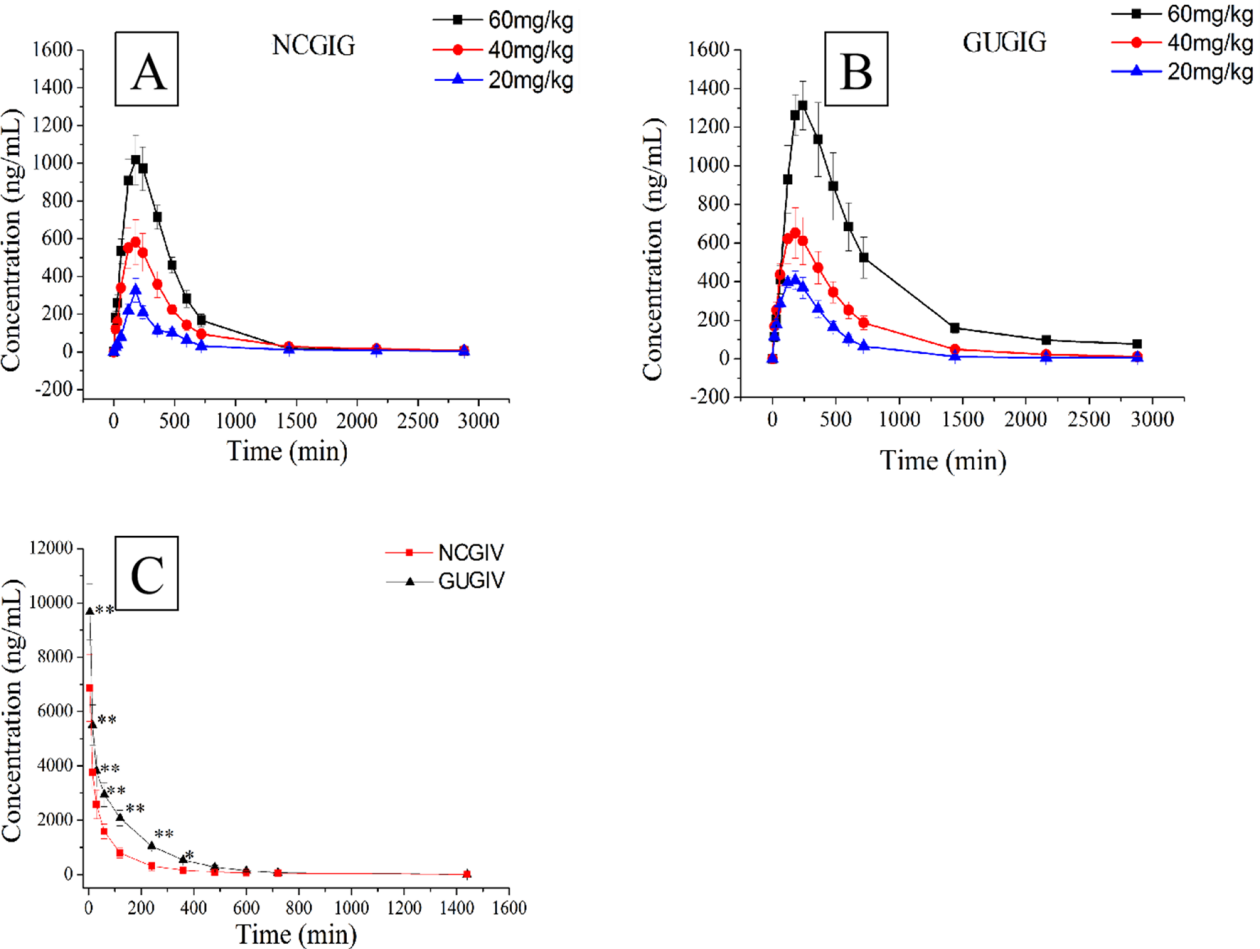
The plasma concentration–time curve of troxipide. (**A**) The NCGIG profiles after oral 20, 40 and 60 mg/kg troxipide; (**B**) The GUGIG profiles after oral 20, 40 and 60 mg/kg troxipide; (**C**) the NCGIV and GUGIV profiles after intravenous injection 40 mg/kg troxipide. *P < 0.05 and **P < 0.01 indicate statistically significant differences in GUGIV compared with that in the NCGIV after intravenous injection.

UHPLC-MS/MS is currently one of the most useful methods for analyzing biological samples due to its high sensitivity and selectivity. The validated UHPLC-MS/MS method was successfully applied to investigate the plasma concentration–time profile of normal control rats and rats with GU that received a single intragastric administration of 20, 40 or 60 mg/kg troxipide (the intragastrically treated normal control group (NCGIG) and intragastrically treated gastric ulcer group (GUGIG), respectively). The doses were selected based on the Chinese Guidance for Nonclinical Pharmacokinetics of Medicinal Products. To study the absolute availability, 40 mg/kg troxipide was administered intravenously. The mean plasma concentration–time profiles are presented in Fig. 7, and the noncompartmental pharmacokinetic parameters (C_max_, T_max_, MRT_(0−t)_, MRT_(0−∞)_, VRT_(0−t)_, VRT_(0−∞)_, t_1/2_, K10, V_z_, AUC_(0−t)_, and AUC_(0−∞)_) of all groups were calculated by DAS 2.0 software and were shown in Table 2. The pharmacokinetics of troxipide in both NCGIG and GUGIG could be fitted to a two-compartment model.

**Table 2:** Pharmacokinetic parameters in the NCG and GUG rat plasma (x ± s, ng/mL).

	Unit	NC				GU			
		IGLNC	IGMNC	IGHNC	IVNC	IGLGU	IGMGU	IGHGU	IVGU
AUC_(0−t)_	ng/mL min	117,685.099 ± 10,645.941	328,401.369 ± 34,148.983**	528,549.285 ± 28,947.109**	471,026.157 ± 29,839.445	220,780.166 ± 27,359.641^++^	424,498.194 ± 33,398.01^6##^^++^	1,180,876.811 ± 211,944.621^++^	890,543.171 ± 108,695.234^++^
AUC_(0−∞)_	ng/mL min	121,549.465 ± 9,918.633	340,315.089 ± 32,473.007**	548,524.618 ± 32,867.392**	484,516.748 ± 30,279.257	27,359.641 ± 7,709.965^++^	428,796.759 ± 33,699.626^##^^++^	1,333,781.847 ± 199,053.201^#^^++^	897,925.201 ± 105,998.307^++^
C_max_	ng/mL	326.523 ± 63.865	784.399 ± 83.091**	1,229.112 ± 213.197**	6,855.744 ± 1,257.845	474.16 ± 75.487^++^	923.821 ± 104.033^##^^+^	1545.947 ± 156.336#^#^^++^	9,666.664 ± 1,040.381^++^
T_max_	min	180.00 ± 0.00	180.00 ± 0.00	180.00 ± 0.00	5.00 ± 0.00	180.000 ± 0.000	180.00 ± 0.00	200.00 ± 30.98	5.00 ± 0.00
t_1/2_	min	453.56 ± 85.81	503.38 ± 90.60	565.90 ± 112.53	210.79 ± 39.33	682.36 ± 113.85^++^	716.65 ± 138.35^++^	776.248 ± 110.877^++^	428.73 ± 60.64^++^
MRT_(0−t)_	min	527.16 ± 94.18	540.44 ± 106.97	622.42 ± 116.77	198.66 ± 30.16	529.26 ± 96.54	578.16 ± 82.43	617.53 ± 124.23	345.25 ± 31.34^++^
MRT_(0−∞)_	min	644.16 ± 108.00	704.16 ± 129.20	723.12 ± 128.71	246.24 ± 38.43	677.07 ± 89.26	768.87 ± 118.66	872.38 ± 142.51	577.07 ± 39.26^++^
VRT_(0−t)_	min2	313,685.843 ± 21,475.069	400,932.399 ± 27,411.201**	528,911.098 ± 37,994.956**	105,439.586 ± 8,439.535	289,338.053 ± 22,390.161	392,313.407 ± 35,441.114^##^	530,205.293 ± 7,134.658^##^	78,199.978 ± 6,343.701^++^
VRT_(0−∞)_	min2	597,386.345 ± 88,064.757	787,738.057 ± 75,689.412**	992,300.21 ± 153,863.791**	159,320.463 ± 18,482.736	540,628.627 ± 75,457.035	783,486.638 ± 59,120.039^##^	1,155,356.02 ± 106,135.933^##^	128,837.383 ± 16,109.524^++^
V_z_	L/kg	0.102 ± 0.015	0.117 ± 0.024	0.123 ± 0.021	0.039 ± 0.004	0.069 ± 0.012^++^	0.061 ± 0.008^++^	0.064 ± 0.003^++^	0.012 ± 0.006^++^
K_a_	1/min	0.014 ± 0.002	0.019 ± 0.002	0.022 ± 0.003	–	0.039 ± 0.006^++^	0.045 ± 0.005^++^	0.054 ± 0.006^++^	–
K_10_	1/min	0.012 ± 0.002	0.018 ± 0.003	0.024 ± 0.005	0.071 ± 0.012	0.007 ± 0.001^++^	0.012 ± 0.002^++^	0.015 ± 0.003^++^	0.016 ± 0.003^++^
AUC_(0−t)_/AUC_(0−∞)_		96.79 ± 2.27	96.45 ± 1.86	96.40 ± 2.22	97.21 ± 0.65	98.47 ± 0.477	99.00 ± 0.30	88.64 ± 8.63	99.15 ± 1.83
F_a_		70.51 ± 8.33				48.13 ± 4.90^++^			

*P < 0.05; **P < 0.01, compared with 20 mg/kg in the NCG. ^#^*P* < 0.05; ^##^*P* < 0.01, compared with 20 mg/kg in the GUG. ^+^*P* < 0.05; ^++^*P* < 0.01, compared with the same dose and administration in the NCG.

The C_max_, K_a_, V_z_, K_10_, t_1/2_, AUC_(0−t)_ and AUC_(0−∞)_ in the NCGIG were significantly different from those of the GUGIG after oral administration of troxipide (20 mg/kg, 40 mg/kg, 60 mg/kg); however, no significant differences in the T_max_, MRT_(0−t)_, MRT_(0−∞),_ VRT_(0−t)_ and VRT_(0−∞)_ were found between the groups. The C_max_ (474.1675.487, 923.821 ± 134.033, 1545.947 ± 156.336 ng/mL) of the GUGIG was slightly increased (*P* < 0.05) compared with the NCGIG (326.523 ± 63.865, 784.399 ± 123.09, 1,229.112 ± 213.197 ng/mL). The K_a_ (0.039 ± 0.006, 0.045 ± 0.005, 0.054 ± 0.006 1/min) of the GUGIG were significantly increased (*P* < 0.01) compared with the NCGIG (0.014 ± 0.002, 0.019 ± 0.002, 0.022 ± 0.003 1/min). More importantly, from the Concentration–time (C–t) curve the troxipide concentration in the GUGIG was slightly increased compared with that in the NCGIG, suggesting that troxipide was more easily absorbed into the blood of rats with GU than that of NC rats^41^. The T_max_ of the GUGIG was not significantly different from that of the NCGIG, which suggested that the T_max_ not related to dose and disease; this result is consistent with the findings of Gao’s report^23^. The V_z_ (0.069 ± 0.012, 0.061 ± 0.008, 0.064 ± 0.003 L/kg) of the GUGIG was significantly decreased (*P* < 0.01) compared with the V_z_ (0.102 ± 0.015, 0.117 ± 0.024, 0.123 ± 0.02 L/kg) of the NCGIG, which suggested that the drug was more slowly distributed in GUGIG rats than in NCGIG rats. The K_10_ (0.007 ± 0.001, 0.012 ± 0.002, 0.015 ± 0.003 1/min) of the GUGIG were significantly decreased (*P* < 0.01) compared with the K_10_ (0.012 ± 0.002, 0.018 ± 0.003, 0.024 ± 0.005) of the NCGIG. The t_1/2_ (682.365 ± 113.857, 716.656 ± 138.357, 776.248 ± 110.877 L/min) of the GUGIG was significantly decreased (*P* < 0.01) compared with the t_1/2_ (453.561 ± 85.809, 503.385 ± 90.600, 565.902 ± 112.531) of the NCGIG. The K_10_ and t_1/2_ results indicated that the excretion rate of the drug was decreased in the GUGIG compared with the NCGIG. The AUC_(0−t)_ (117,685.099 ± 10,645.94 − 528,549.285 ± 28,947.109 μg/L min) and AUC_(0−∞)_ (121,549.465 ± 9,918.633 − 548,524.618 ± 32,867.392 μg/L*min) of the GUGIG were significantly increased (*P* < 0.05) compared with the AUC_(0−t)_ and AUC_(0−∞)_ of the NCGIG, and these results were consistent with the C_max_ results. Furthermore, there were no significant differences in the MRT_(0−t)_, MRT_(0−∞)_, VRT_(0−t)_ or VRT_(0−∞)_ between the GUGIG and the NCGIG.

In addition, significant differences (*P* < 0.05) in the C_max_, VRT_(0−t)_, VRT_(0−∞)_, AUC_(0−t)_ and AUC_(0−∞)_ were observed in the NCGIG after oral administration of different doses of troxipide (20 mg/kg, 40 mg/kg, 60 mg/kg), but no significant differences in the K_a_, V_z_, K_10_, t_1/2_, T_max_, MRT_(0−t)_ or MRT_(0−∞)_ were observed. Moreover, the C_max_, K_a_, T_max_, V_z_, K_10_, t_1/2_, AUC_(0−t)_, AUC_(0−∞)_, MRT_(0−t)_, MRT_(0−∞),_ VRT_(0−t)_ and VRT_(0−∞)_ were similar between the GUGIG and NCGIG after oral administration of different doses of troxipide. Linear regression analysis of the oral administration data (NCGIG and GUGIG) showed that the C_max_ was dose-dependent at concentrations between 20 to 60 mg/kg, and the linear equations of the C_max_ in the NCGIG and GUGIG were y = 22.565x − 122.58, R^2^ = 0.9999 and y = 26.795x − 90.478, R^2^ = 0.9914, respectively (Fig. S4). There was a linear increase in the AUC_(0−t)_ following oral administration of troxipide at the studied doses, and the linear equations of the AUC_(0−t)_ in the NCGIG and GUGIG were y = 10272x − 85986, R^2^ = 0.9998 and y = 24002x − 351378, R^2^ = 0.9005, respectively (Fig. S5). The linear equations of the AUC_(0−∞)_ in the NCGIG and GUGIG were y = 10674x − 90179, R^2^ = 0.9998 and y = 27825x − 451882, R^2^ = 0.8844, respectively (Fig. S6). Thus, the C_max_, AUC_(0−t)_ and AUC_(0−∞)_ of troxipide were dose-dependent at concentrations between 20 to 60 mg/kg, indicating that the pharmacokinetic behavior of troxipide in rats was linear with respect to dose.

After intravenous injection of 40 mg/kg troxipide, the AUC_(0−t)_, AUC_(0−∞)_, C_max_, t_1/2_, MRT_(0−t)_, MRT_(0−∞)_, VRT_(0−t)_, VRT_(0−∞)_, V_z_ and K_10_ of the intravenously injected gastric ulcer group (GUGIV) were significantly different (*P* < 0.05) from the AUC_(0−t)_, AUC_(0−∞)_, C_max_, t_1/2_, MRT_(0−t)_, MRT_(0−∞)_, VRT_(0−t)_, VRT_(0−∞)_, V_z_ and K_10_ of the intravenously injected normal control group (NCGIV). These results suggested that in this study, the pharmacokinetics of troxipide in were dramatically altered in rats with GU compared with NC rats, which is consistent with the results presented in Fig. 7.

The AUC_(0−t)_/AUC_(0−∞)_ of the NCGIG and GUGIG were 96.40 ± 2.22% and 88.64 ± 8.63% (far higher than 80.0%), respectively, which indicated that the time elapsed before blood sampling was sufficient to detect the pharmacokinetic parameters of troxipide with high accuracy and precision. Additionally, according to the above calculation, absolute bioavailability following oral administration in the NCGIG and GUGIG were 70.51 ± 8.33% and 48.13 ± 4.90%, respectively. The absolute bioavailability in the GUGIG was significantly decreased (*P* < 0.01) compared with that in the NCGIG, possibly due to the slower distribution and reduced excretion in the GU rats^42, 43^. This study of the pharmacokinetics of troxipide may provide accurate and detailed support for further development and clinical application of this drug in the near future. Meanwhile, structural modifications, dosage form changes and drug combinations need to be further investigated to prevent adverse reactions to troxipide.

#### Tissue distribution study

**Figure 8:**
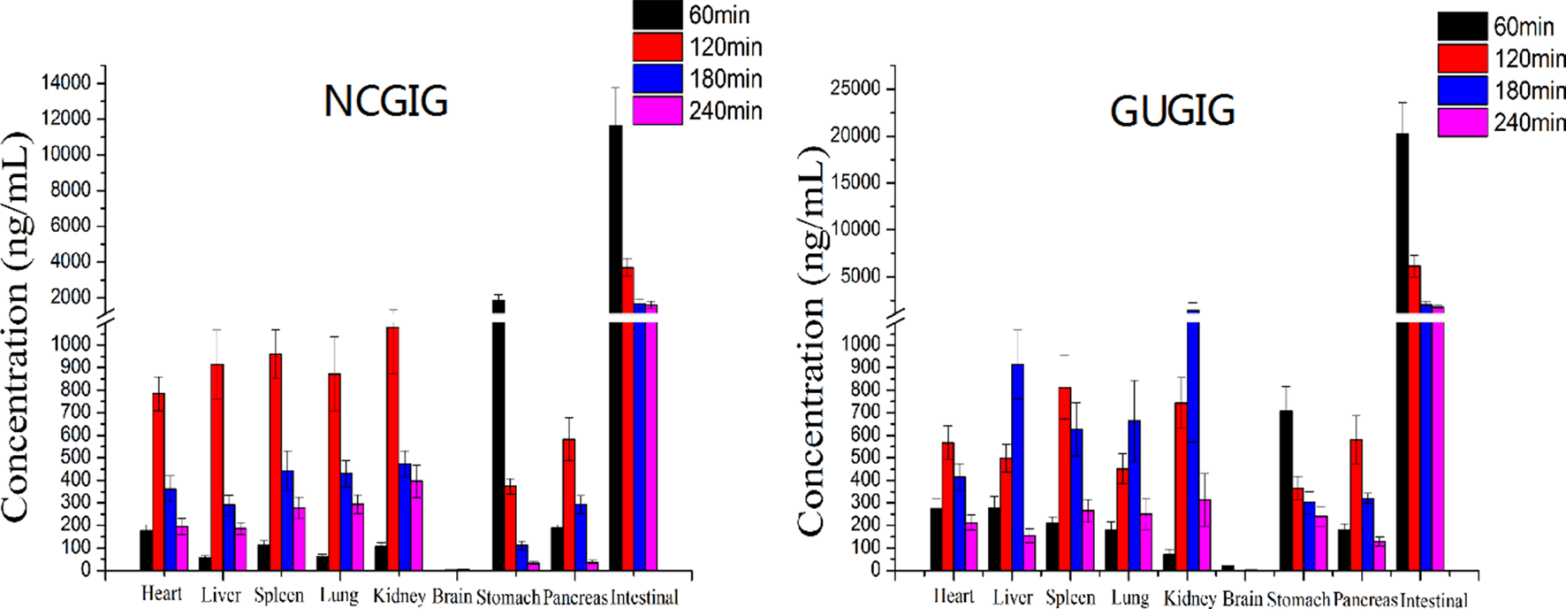
The tissue distribution of troxipide in the NCGIG (40 mg/kg) and GUGIG (40 mg/kg) after oral administration.

Here, we performed the study of tissue distribution in heart, liver, spleen, lung, kidney, brain, stomach, intestinal, pancreas by the above UHPLC-MS/MS method. The tissues distribution of troxipide in the NCGIG and GUGIG after oral administration (40 mg/kg) are shown in Fig. 8. The results suggested that troxipide could be quickly detected in the organs of the rats after oral administration, which demonstrated that troxipide was rapidly distributed to a variety of tissues. Specifically, the trend of the change in troxipide concentration in most tissues was consistent with the pharmacokinetic behavior of the drug in the plasma. However, the distribution of troxipide in the brain, stomach and intestines was unique. Troxipide was barely detectable in the brain, which indicates that it cannot cross the blood–brain barrier or cause brain damage. In the NCGIG and GUGIG, the highest concentration of troxipide in the stomach and intestines was detected at 1 h, which was inconsistent with the pharmacokinetic behavior of troxipide in the plasma. These results may have been because the stomach and intestines are the main site of drug absorption^44^. The pharmacokinetic behavior of troxipide in the GUGIG was significantly different from that in the NCGIG. For example, in the GUGIG, the highest concentration of troxipide in the liver, lung and kidney was observed at 3 h, but in the NCGIG, the highest troxipide concentration in the liver, lung and kidney was observed at 2 h. These results suggested that GU can cause poor absorption and membrane permeability in the liver, lung and kidney, which is consistent with the findings of Liu et al.^45^. The above reasons may be due to the absorption and distribution of patients obviously different from that of healthy human.

### Pharmacodynamic study

#### Effect of troxipide on biochemical indexes and stomach histopathology in rats with GU

**Figure 9:**
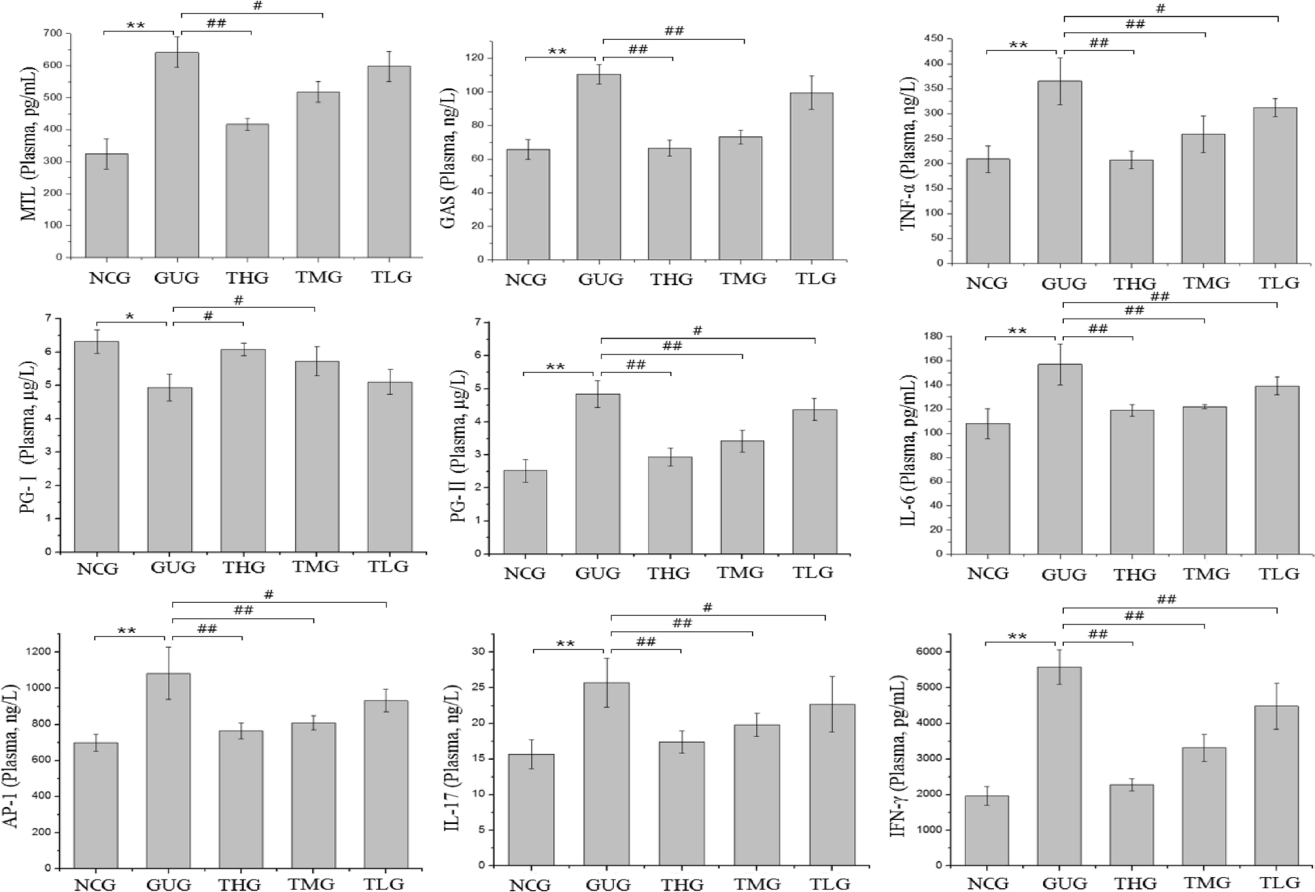
MTL, GAS, TNF-α, PG-Ӏ, PG-II, IL-17, IFN-γ, AP-1 and IL-6 levels in the rat plasma after troxipide treatment. NCG, normal group (0.9% normal saline 10 mL kg^−1^ day^−1^); GUG, gastric ulcer group (5% acetic acid 10 mL kg^−1^ day^−1^); THG, high-dose troxipide group (5% acetic acid 10 mL kg^−1^ day^−1^ + Troxipide 60 mg kg^−1^ day^−1^); TMG, medium-dose troxipide group (5% acetic acid 10 mL kg^−1^ day^−1^ + Troxipide 40 mg kg^−1^ day^−1^); TLG, low-dose troxipide group (5% acetic acid 10 mL kg^−1^ day^−1^ + Troxipide 20 mg kg^−1^ day^−1^). Values are presented as means ± SD for all groups (n = 10). *P < 0.05 and **P < 0.01 indicate statistically significant differences when the GUG is compared with the NCG. ^#^*P* < 0.05 and ^##^*P* < 0.01 indicate statistically significant differences when the troxipide treatment groups is compared with the GUG.

In the present study, body weight, food intake and water intake were recorded and measured during the experimental period (Tables S8, S9, S10). Significant differences in body weight, food intake and water intake were observed between the normal control group (NCG) and the gastric ulcer group (GUG). However, in the rats with GU, these values gradually recovered to normal levels after troxipide administration for another 2 weeks, which suggested that troxipide can have beneficial effects on rats with GU. The pathogenesis of GU is multifactorial and includes complex interactions between so-called aggressive and protective factors, in which inflammatory cytokines (such as IL-17, IL-6, TNF-α, IFN-γ and AP-1) play an important role^46–52^. Before oral administration of troxipide, the plasma levels of cytokines (IL-17, IL-6, TNF-α, IFN-γ and AP-1) were significantly increased in the GUG compared to the NCG (Fig. S7). Thus, inflammation and gastric mucosal damage levels were worse in the GUG than in the NCG due to the interaction among IL-17, IL-6, TNF-α, IFN-γ and AP-1^53–59^. After 2 weeks of troxipide treatment, the levels of these cytokines in the troxipide-treated groups were significantly lower than those in the GUG (Fig. 9), suggesting that GU was significantly alleviated after oral administration of troxipide. Furthermore, high-dose troxipide (THG) had a more significant therapeutic effect than low-dose troxipide, indicating that troxipide alleviated GU in a dose-dependent manner.

Moreover, MTL, GAS, PG-Ӏ and PG-II are acknowledged as diagnostic markers of GU. MTL and GAS are important gastrointestinal hormone that can stimulate parietal cells to secrete hydrochloric acid and chief cells to secrete pepsinogen^60–63^. PG-Ӏ and PG-II, which reflects the physiological or pathophysiological function of the gastric system, is secreted by the pyloric glands and Brunner’s glands as well as chief cells and mucous neck cells^64–68^. Therefore, we investigated the levels of MTL, GAS, PG-Ӏ and PG-II in all groups. The levels of these markers before oral administration of troxipide are shown in Fig. S7. MTL, GAS and PG-II levels were increased (*P* < 0.05) in the GUG compared with the NCG, while the PG-Ӏ level was decreased (*P* < 0.05) in the GUG. These results are consistent with the findings of Suo, Cao and Ghoshal et al.^69–71^. The levels of MTL, GAS and PG-II were significantly decreased (*P* < 0.01, *P* < 0.01, and *P* < 0.01, respectively) and the PG-Ӏ level was significantly increased (*P* < 0.01) in the troxipide-treated groups compared with the GUG after 2 weeks of troxipide treatment. Consistently, in the GUG, the PG-Ӏ level was low, and the PG-II level was high; thus, the PG-Ӏ/PG-II ratio was reduced in the GUG compared to the NCG (Fig. 9), which is consistent with the findings of Ghoshal and Kumar et al.^70,72^. These results indicated that gastric injury occurred in the GUG.

**Figure 10:**
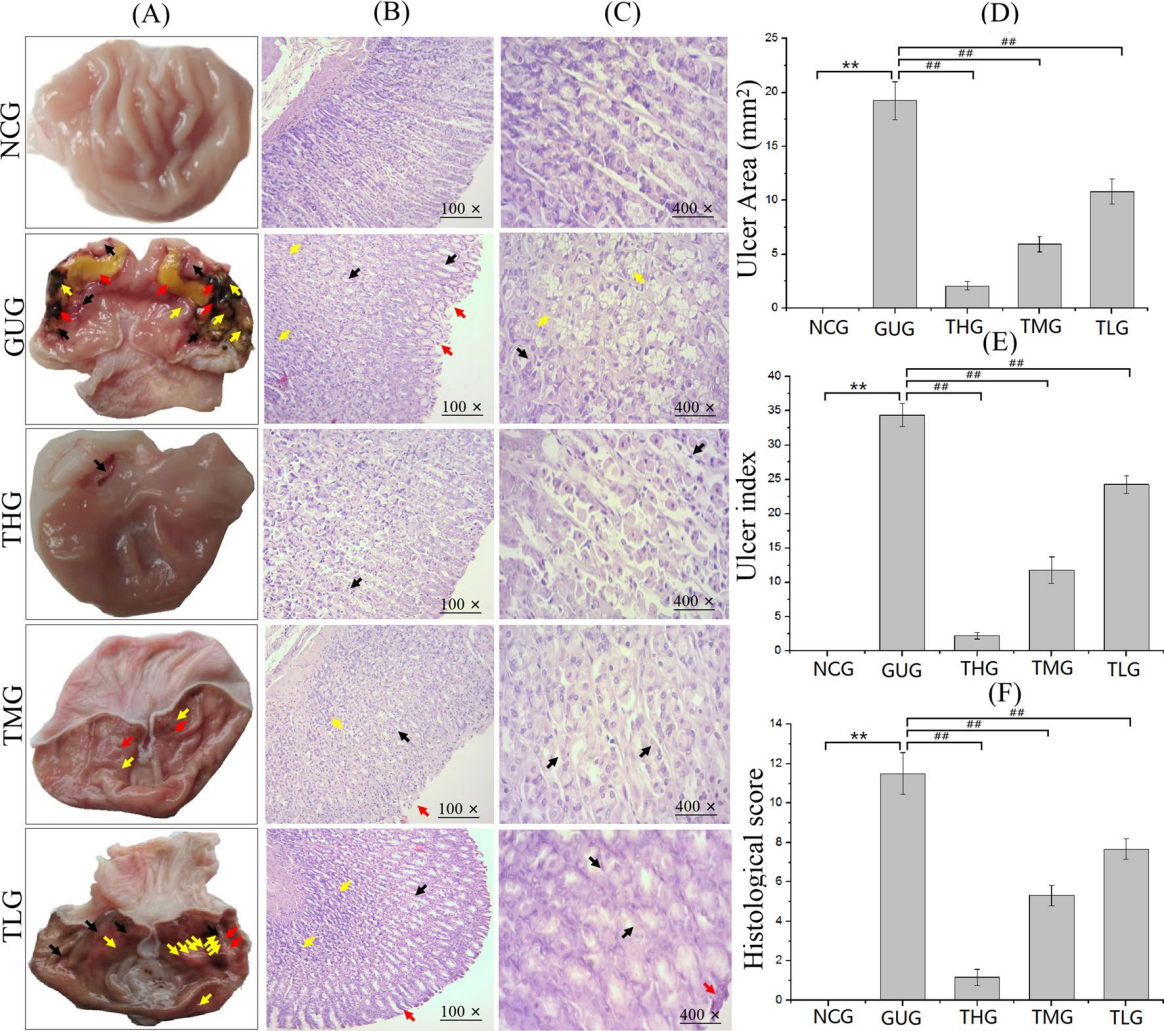
Macroscopic and microscopic analysis of stomach tissue after troxipide treatment. (**A**) Macroscopic analysis of GU after troxipide treatment. (**B**) Sections of the gastric mucosa (H&E staining) (100×). (**C**) Sections of the gastric mucosa (H&E staining) (400×). (**D**) Ulcer area. (**E**) Ulcer index. (**F**) Histological scores. NCG, normal group (0.9% normal saline 10 mL kg^−1^ day^−1^); GUG, gastric ulcer group (5% acetic acid 10 mL kg^−1^ day^−1^); THG, high-dose troxipide group (5% acetic acid 10 mL kg^−1^ day^−1^ + Troxipide 60 mg kg^−1^ day^−1^); TMG, medium-dose troxipide group (5% acetic acid 10 mL kg^−1^ day^−1^ + Troxipide 40 mg kg^−1^ day^−1^); TLG, low-dose troxipide group (5% acetic acid 10 mL kg^−1^ day^−1^ + Troxipide 20 mg kg^−1^ day^−1^). Values are presented as means ± SD for all groups (n = 10). *P < 0.05 and **P < 0.01 indicate statistically significant differences when the GUG is compared with the NCG. ^#^*P* < 0.05 and ^##^*P* < 0.01 indicate statistically significant differences when the troxipide treatment groups is compared with the GUG.

Before troxipide treatment, there were significant differences between the NCG ang GUG at the macroscopic level (Fig. S8A). In the GUG, gastric injury, as evidenced by loss of mucosal folding, edema, hemorrhage, ulcer perforation and so on, was observed, which indicated that the model of GU had been successfully established. After 2 weeks of troxipide treatment, gastric injury was significantly reduced in the troxipide-treated groups compared to the GUG (Fig. 10A), suggesting that troxipide can alleviate gastric injury. The ulcer area and ulcer index are shown in Fig. 10D,E. The results suggested that the ulcer area and ulcer index were markedly increased (*P* < 0.01) in the GUG compared to the NCG, high-dose troxipide group (THG), medium-dose troxipide group (TMG) and low-dose troxipide group (TLG). Ulcer inhibition levels are shown in Table S11. Ulcer inhibition was 100% in the NCG and 0% in the GUG. However, ulcer inhibition in the THG, TMG and TLG (89.3%, 69.2% and 43.8%, respectively) was significantly increased compared with that in the GUG. These results are consistent with the results of biochemical indexes presented above. Images of hematoxylin and eosin (H&E) staining prior to troxipide treatment are shown in Fig. S8B (100×) and Fig. S8C (400×). Edema, inflammatory infiltration, degeneration and necrosis were observed in the GUG but not the NCG. After 2 weeks of troxipide treatment, the extent of gastric injury in the troxipide-treated groups was markedly reduced compared with that in the GUG group (Fig. 10B,C). The H&E scoring results, which are in good agreement with the macroscopic observations, are shown in Fig. 10F. These results suggest that troxipide is effective in treating GU.

#### Effect of troxipide on the expression of the apoptosis-related proteins HSP-90, C-Cas-3 and C-PARP-1 in the stomach of rats with GU

**Figure 11:**
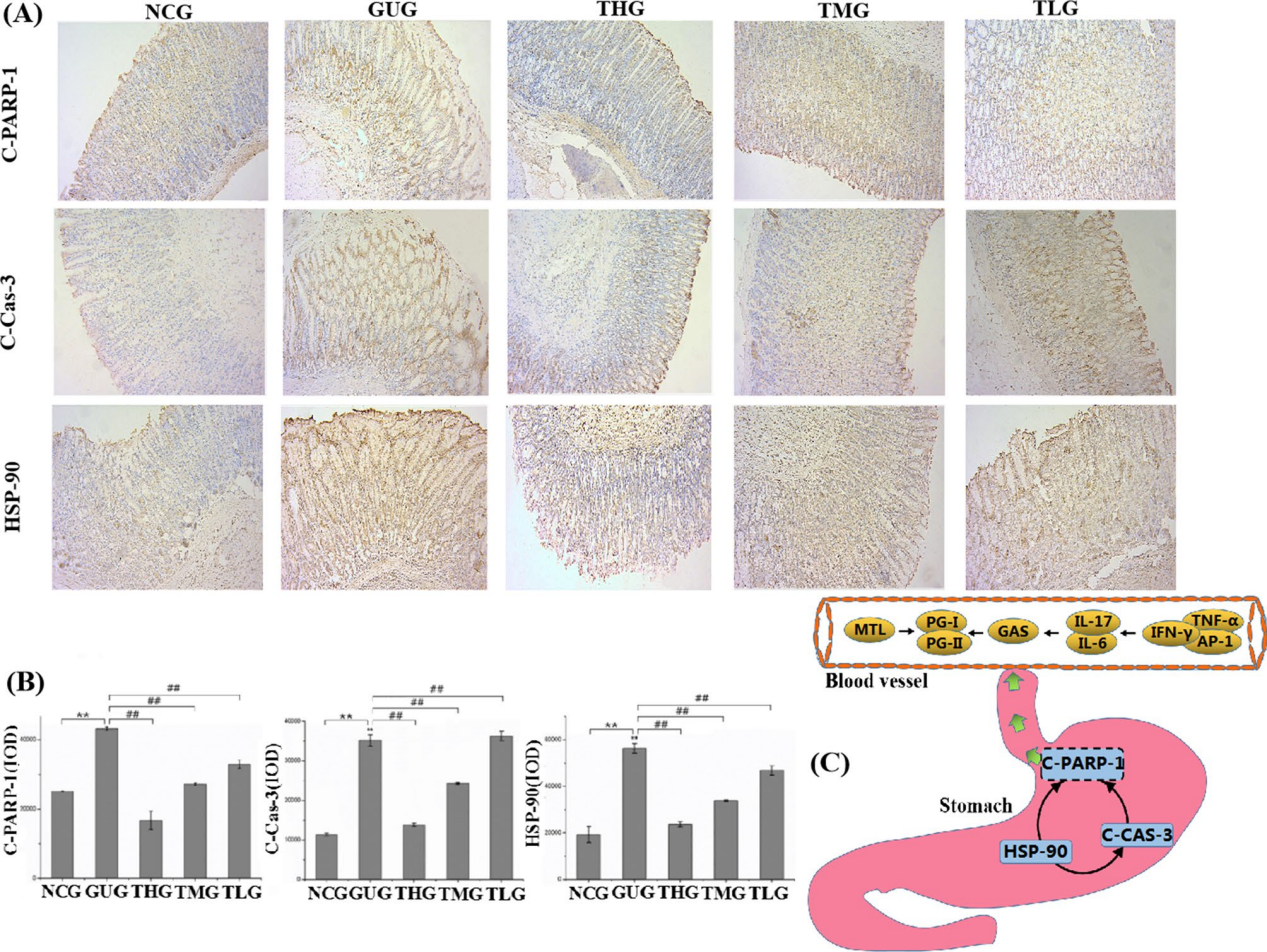
Troxipide alleviated GU by regulating the expression of HSP-90, C-CAS-3 and C-PARP-1 in rats with GU. (**A**, **B**) The expression levels of HSP-90, C-CAS-3 and C-PARP in the stomach were evaluated by immunohistochemical staining (100×). (**C**) Possible pathways underlying the development of GU in vivo in rats. NCG, normal group (0.9% normal saline 10 mL kg^−1^ day^−1^); GUG, gastric ulcer group (5% acetic acid 10 mL kg^−1^ day^−1^); THG, high-dose troxipide group (5% acetic acid 10 mL kg^−1^ day^−1^ + Troxipide 60 mg kg^−1^ day^−1^); TMG, medium-dose troxipide group (5% acetic acid 10 mL kg^−1^ day^−1^ + Troxipide 40 mg kg^−1^ day^−1^); TLG, low-dose troxipide group (5% acetic acid 10 mL kg^−1^ day^−1^ + Troxipide 20 mg kg^−1^ day^−1^). Values are presented as means ± SD for all groups (n = 10). *P < 0.05 and **P < 0.01 indicate statistically significant differences when the GUG is compared with the NCG. ^#^*P* < 0.05 and ^##^*P* < 0.01 indicate statistically significant differences when the troxipide treatment groups were compared with the GUG.

Apoptosis and inflammation play a significant role in GU pathogenesis, and cell apoptosis in the gastric mucosa is closely related to ulcers^6,73,74^. Therefore, the protein expression of HSP-90, C-Cas-3 and C-PARP-1 in the rats was also investigated. HSP-90 has been shown to induce apoptosis in various cells, and C-PARP-1 and C-Cas-3 are major apoptotic markers. HSP-90 is a heat shock protein that can promote the transportation, folding, and rearrangement of other proteins as a “molecular chaperone”^75,76^. Many recent studies have reported that HSP-90 is induced when cells are exposed to sublethal stressors, such as inflammation, infection or ischemia^77,78^. In our study, a small number of yellow/brown areas were observed in the tissues of rats from the NCG, suggesting low expression levels of HSP-90. Thus, we speculated that HSP-90 levels would be upregulated (*P* < 0.01) in the GUG compared with the NCG, and the results were in agreement with our hypothesis. C-Cas-3, a protease with multiple functions, plays a key role in cell apoptosis and is responsible for the cleavage of PARP^79^. Previous studies have shown that inflammation can activate the mitochondrial apoptotic pathway, thereby activating the caspase cascade^80^. Our results demonstrated that troxipide downregulated (*P* < 0.01) the expression of C-Cas-3, as shown in Fig. 11A,B. C-PARP-1 cleaves PARP-1, which is a marker of apoptosis and is presumed to play a role in DNA repair^81–83^. The protein expression rats of C-PARP-1 was significantly upregulated (*P* < 0.01) (Fig. 11A,B) in the GUG compared to the NCG, which is consistent with previous studies^84^.

Overall, GU is the result of an interaction between inflammation and apoptosis. On the one hand, inflammatory factors (IL-17, IL-6, TNF-α, IFN-γ and AP-1) promote protein expression of HSP-90, C-Cas-3 and C-PARP-1 and further stimulate cell apoptosis to aggravate gastric injury. On the other hand, cell apoptosis also promotes inflammation and further aggravates gastric injury levels. Moreover, GU promotes changes in MTL, GAS, PG-Ӏ, PG-II and PG-Ӏ/PG-II levels (Fig. 11C). Thus, the above results indicate that troxipide alleviates GU by increasing the expression of inflammatory factors and apoptosis-related proteins.

## Conclusion

In summary, for the first time, we studied the metabolism and pharmacokinetics of troxipide in rats with 5% acetic acid-induced GU. The drug study on the disease rats was closer to the clinical application of the drug, because the pharmacodynamics mechanism of patients is obviously different from that of healthy human. A total of 45 metabolites of orally administered troxipide were studied in vivo in rats for the first time. The pharmacokinetic parameters observed for troxipide were significantly different in GU rats when compared with NC rats.

Studying the pharmacokinetics and metabolism of troxipide is helpful for elucidating its potential pharmacological mechanisms and for its development as a drug for clinical application^85^. Therefore, the metabolism, pharmacokinetics and pharmacodynamics of troxipide in vivo in rats deserve further attention due to the importance of troxipide in the treatment of GU in this study. Metabolic studies can provide comprehensive insight into safety monitoring and the design of drug delivery systems^86–89^. Although the structures of the metabolites could not be determined conclusively by LC–MS/MS alone, the results significantly expanded our understanding of the pharmacological effects and therapeutic mechanism of troxipide and its associated metabolic pathways, and these metabolites could be targets for future studies on the structural modification of troxipide and further development of new drugs. This study provides an available strategy for identifying and characterizing metabolic reactions in the further study. Here, we developed a simple, rapid and sensitive method for quantifying troxipide levels in biosamples, including plasma, urine, and fecal samples, that can be successfully used to study the pharmacokinetics of troxipide and its major metabolites in GU rats after oral administration in accordance with the guidelines of FDA, EMA and ICH . The data were complex and multifarious, and artificial intelligence and big data banks could be used to identify and characterize metabolites in the future.

The results of the pharmacokinetic study suggested that the absorption of troxipide was slightly increased, and the distribution and excretion of the drug were decreased in rats with GU compared with NC rats. The absolute bioavailability and tissue distribution of troxipide following oral administration were significantly different between rats with GU and NC rats. This study fills a gap in the literature and provides a theoretical basis for the reasonable use of troxipide for individualized therapy. In addition, this work also identified the main mechanisms by which inflammatory factors and apoptosis are inhibited in the stomachs of rats with GU. Moreover, our macroscopic, microscopic and biochemical analyses demonstrated that enhancing blood circulation and metabolism at the site of GU can alleviate gastric mucosal injury and maintain gastric mucosal homeostasis.

Overall, this systematic and comprehensive study of the metabolism, pharmacokinetics and pharmacodynamics of troxipide and its major metabolites in vivo in rats with GU provides important reference information regarding the clinical application of troxipide in the study.

## Materials and methods

### Chemicals and reagents

The chemicals and reagents of study were detailed shown in Supplementary Materials and Methods.

### Animals and ethical statement

Male, Specific pathogen Free (SPF), 8–12 weeks old Sprague–Dawley (SD) rats of weighing 200 ± 20 g were obtained from the Vital River Laboratory Animal Technology (certification number: 11400700243083, license No. SCXK (Jing) 2016-0006, Beijing, China). All SD rats were maintained in pathogen-free facility standard cages at constant temperature (25 ± 2 °C) and humidity (55 ± 5%) with 12-h light/dark cycle, and can access to water and food ad libitum. Moreover, all animals were acclimated in accordance to the above condition in the laboratory for 1 week prior to the experiments in this study. In all protocols and procedures, maintenance and treatment of the animal experiment were performed in accordance to guideline on administration of lab animals by State Council and National Institute of Health for the Care, and approved by Animal Ethical and Welfare Committee (AEWC) of Hebei University (He Bei, China; approval number: IACUC-2018048 in 2018). All in all, all effects were made to alleviate unnecessary suffering and distress of animals.

### Analytical methodology

#### UHPLC TSQ Altis MS instrument conditions

The ultra high performance liquid chromatography TSQ Altis tandem mass spectrometry system (UHPLC TSQ Altis MS) consisted of a Vanquish UHPLC system (Thermo Scientific, USA) and a Thermo Scientific TSQ Altis Triple Quadrupole mass spectrometry (Thermo Scientific, USA). The analytical column was an ACQUITY UHPLC BEH-C18 column (1.7 μm, 2.1 mm × 50 mm, Waters, USA). A binary mobile solvent composed of phase A (0.1% formic acid in water) and phase B (acetonitrile) at a flow rate of 0.3 mL/min. The procedure of gradient elution was as follows: 5–5% B at 0–1.0 min, 5–100% B at 1.0–7.0 min, 100–100% at 7.0–10.0 min, 100–5% B at 10.0–10.2 min, 5–5% B at 10.2–12 min. The temperatures of column and auto-sampler room were maintained at 40 °C and 10 °C, respectively. The injection volume of the sample was 2 µL. The mass spectrometer was applied with electrospray ionization (ESI) source in selective reaction monitoring (SRM) mode. The optimized instrument parameters were shown as below: positive ion, 3,500 V; negative ion, 2,500 V; sheath Gas, 50 Arb; Aux gas, 23.2 Arb; sweep Gas, 0 Arb; ion transfer tube temperature, 52 °C; vaporizer temperature, 350 °C. The finalized precursor → product transitions of troxipide and carbamazepine were m/z 295.072 → 195.071 and m/z 237.012 → 179.071, respectively.

#### UHPLC-Q-Orbitrap-HRMS instrument conditions

*The UHPLC-Q-Orbitrap-HRMS system was equipped with a Dionex Ultimate 3,000 UHPLC system (Thermo Scientific, USA) and a Q-Exactive hybrid quadrupole-Orbitrap mass spectrometer (Thermo Scientifiic, USA) with electrospray ionization (ESI). Chromatographic separation was performed using a ACQUITY UHPLC BEH C18 column (2.1 mm × 50 mm, 1.7 µm, Waters, USA) at 40 °C. Acetonitrile (phase A) and 0.1% formic acid in water (phase B) were used as the mobile phases at a flow of 0.35 mL/min. The procedure of gradient elution was as follows: 0–1 min, 5% A; 1–9 min, 5–100% A; 9–12 min, 100% A; 12–12.1 min, 100–5% A; 12.1–15 min 5% A. The injection volume of sample was 5 µL at 4 °C. The Q-Exactive hybrid quadrupole-Orbitrap mass spectrometer was an analytical instruments of rapid, sensitive, precision and accuracy for detecting unknown metabolites in bio-logical samples by the following parameters: sheath gas 30 Arb, auxiliary 10 Arb, sweep gas 0 Arb, positive spray voltage + 3.5 kV, negative spray voltage -2.8 kV, capillary temperature 320 °C, auxiliary gas heater temperature 300 °C and S-lens RF 50 V. The full scan mode of positive and negative were used by the apparatus under nitrogen conditions, and the ranges of full scan mode were from 50 to 1,200 m/z. All data were acquired under preset model, and processed using Compound Discovery (CD, Thermo Scientific, USA) and extracted mass spectrometry using Xcalibur 3.0 software (Thermo Scientific, USA).

#### Preparation of calibration standard and quality control (QC) samples

A 100 µg/ml standard stock solution was prepared by dissolving 1.0 mg of accurately weighed troxipide in methanol in a 10-mL volumetric flask. The standard stock solution was serially diluted with methanol to obtain several standard working solutions with concentrations of 0.05, 0.15, 0.2, 0.4, 0.5, 1, 2, 5, 10, 50, 80 and 100 µg/mL. Plasma and tissue calibration standards were prepared by diluting the corresponding standard working solutions with blank rat plasma and tissue to concentrations of 5, 15, 20, 40, 50, 100, 200, 500, 1,000, 5,000, 8,000 and 10,000 ng/mL troxipide. The concentrations of the QC samples (plasma and tissue) were 15, 400 and 8,000 ng/mL troxipide. A 100 µg/ml IS stock solution was made by transferring approximately 1.0 mg of accurately weighed IS to a 10-mL volumetric flask and dissolving it in methanol. The IS stock solution was further diluted with methanol to obtain a 1 µg/mL IS working solution. All solutions and samples were immediately stored at 4 °C until analysis.

### Sample preparation

Sample preparation of metabolite identification and pharmacokinetic study were detailed shown in Supplementary Materials and Methods.

### Metabolism study

To investigate the major metabolites of troxipide, we performed a metabolic study. For this experiment, a rat model of GU (n = 20) was established successfully as described above by using 5% acetic acid (10 mL/kg/day). Each rat with GU was placed in a metabolic cage for 3 days before oral administration for the adaptation period. Then, the rats with GU were orally administered troxipide (40 mg/kg) (n = 10) or an equivalent volume of saline (n = 10). Blood samples (0.3 mL) from the orbital vein were collected in heparinized tubes before administration and 15, 30, 60, 120, 240, 480, 720, 1,440, and 2,880 min after administration. The samples were centrifuged at 5,000 rpm for 10 min at 4 °C to obtain plasma. The urine and feces of each rat were collected between oral administration 0–48 h, and then the urine was centrifuged at 3,000 rpm at 4 °C for 10 min. The supernatant was transferred to a new tube. All biological samples were stored at -80 °C until analysis.

Raw metabolic data were obtained by systematic analysis using UHPLC-Q-Orbitrap-HRMS. In brief, to identify the metabolites of troxipide, the base peak chromatograms of samples from troxipide-treated rats and saline-treated rats were analyzed and compared. The metabolic data were processed by Compound Discovery (Thermo Scientific, USA) software and Mass Frontier software (Thermo Scientifiic, USA).

### Method validation

The methods were based on the FDA and EMA guidelines to determine troxipide in rat plasma and tissue by validating analytical method, as detailed in Supplementary Materials and Methods.

### Pharmacokinetic study

110 SD rats were randomly divided into two groups according to the average body weight: NCG (n = 55) and GUG (n = 55). The rats of GUG were orally administrated 5% acetic acid (10 mL/kg/day) at 9.00 am for 2 weeks. Meantime, the rats of NCG were given an equivalent volume of saline.

#### Intragastric study

After 2 weeks, NCGIG rats (n = 30) and GUGIG rats (n = 30) were randomly divided into three groups. All rats were fasted for at least 12 h before the administration but were given free access to water. The NCGIG rats and GUGIG rats were weighed and given a single dose of 20 mg/kg, 40 mg/kg, or 60 mg/kg troxipide (10 mL/kg) via intragastric administration. The rat blood collection method used in our study was performed according to ICH guidelines and the guidelines of the Nonclinical Pharmacokinetics of Medicinal Products by National Medical Products Administration (NMPA) and was based on previous reports and our preliminary studies^90–92^. The approximately 0.3 mL of plasma was collected from the orbital venous plexus of the NCGIG and GUGIG rats before administration and 15, 30, 60, 120, 180, 240, 360, 480, 600, 720, 1,440, 2,160, 2,880 min after administration .

#### Intravenous study

After 2 weeks, NCGIV rats (n = 10) and GUGIV rats (n = 10) were weighed and intravenously injected with a single dose of 40 mg/kg troxipide (10 mL/kg) via the tail vein to evaluate absolute availability. Approximately 0.3 mL of plasma was collected from the NCGIV rats and GUGIV rats before after intravenous injection and 5, 10, 30, 60, 120, 180, 240, 360, 480, 600, 720, and 1,440 min after intravenous injection. After collection, the plasma samples were immediately centrifuged at 3,000 rpm for 10 min at 4 °C, and the resulting supernatants were collected and stored at -80 °C until analysis.

#### Tissue distribution studies

NCGIG rats (n = 15) and GUGIG rats (n = 15) were randomly divided into five groups according to execution time. All rats were weighed and orally administered 40 mg/kg troxipide (10 mL/kg). Then, the rats were sacrificed on ice before treatment or 60, 120, 180, 240 min after oral administration. Tissues were quickly excised, carefully rinsed with saline (pH 7.0) to remove blood from the tissue surface, dried with filter paper and weighed. A total of 1.0 g of each tissue sample was accurately weighed (if the total weight of the tissue sample was < 1.0 g, the entire sample was used, and the precise weight was recorded) and homogenized with physiological saline (0.33 g/mL) by using a tissue homogenizer. Finally, all tissue homogenates were stored at − 80 °C until analysis.

### Pharmacodynamics study

After a week of acclimatization, Sprague–Dawley (SD) rats were randomly assigned to the NCG (n = 16) or GUG (n = 46). Prior to the initiation of troxipide treatment, the GUG rats were given 5% acetic acid (10 mL kg^−1^) by oral gavage every day at 9:00 am for 2 weeks. The NCG rats were orally administered an equivalent volume of saline. After 2 weeks, to investigate whether the GU model was successfully established, 6 rats were randomly chosen from each group and sacrificed. Then, the GUG rats were randomly divided into the following four groups (n = 10): the GUG, THG, TMG and TLG. The rats in the GUG, THG, TMG and TLG groups were orally administered 5% acetic acid (10 mL kg^−1^) every day at 9.00 am for another 2 weeks. The rats in the TMG and TLG and THG were orally gavaged with 20 mg kg^−1^, 40 mg kg^−1^ or 60 mg kg^−1^ troxipide every day at 4:00 pm. The rats in the NCG and GUG were orally administered an equivalent volume of saline every day at 4:00 pm. The dose of troxipide was calculated and adjusted according to a previous study and our preliminary experiment. All rats were given a normal diet and fresh water ad libitum throughout the experiment. After 2 weeks of troxipide treatment, the rats were fasted for 12 h and then anesthetized via intraperitoneal (ip) injection of 3% pentobarbital (40 mg/kg). Serum was collected and stored at -80 °C, and stomach tissue samples were collected after the rats were sacrificed, fixed in 10% formalin, rapidly frozen in liquid nitrogen and stored at -80 °C.

Enzyme-linked immunosorbent assay (ELISA) kits were utilized for measurement of rats serum of Motilin (MTL), Gastrin (GAS), Pepsinogen I (PG-I), Pepsinogen II (PG-II), Interleukin (IL-17), Interleukin (IL-6), Tumor Necrosis Factor (TNF-α), Interferon-γ (IFN-γ), Activator Protein (AP-1) following the manufacturer’s protocols. The above commercial assay kits were purchased from Jiangsu mbbiology biological technology Co., Ltd. (Jiangsu, China). And, we were investigated the ulcer area (mm^2^), ulcer index and ulcer inhibition (%) in the rats in the study. The stomachs were inflated by injecting 10% formalin solution to fix the tissues overnight, and then opened along the greater curvature and photographed. The GU area (mm^2^) was calculated by the longest lengthand (d_1_) and width (d_2_) of the ulcer using the following equation: $S = \pi (d1/2) \times (d2/2)$, where $S$ represents the ulcer area (mm^2^). And, the gastric tissues were directly observed to determine the ulcerative lesion index according to the severity of GU lesion scoring system. The ulcer index of each rats were calculated in accordance to the scoring criteria of ulcer index (Table S12). Then, the inhibition of ulceration was computed using follow equation:

$${\text{Ulcer Inhibition}}\,(\%) = \frac{{\text{Ulcer Area (GUG group)}} - {\text{Ulcer Area (Troxipide group)}}}{{\text{Ulcer Area (GUG group)}}} \times 100\%$$

To further investigate the stomach injury, the hematoxylin and eosin (H&E) staining was performed in this study. The stomachs from 10% formalin were embedded in paraffin after dehydration by ethanol, xylene transparent. The paraffin sections (5 µm) were acquired using a microtome (HM340E, Thermo Scientific, Germany) and were stained with hematoxylin and eosin. The sections were observed by light microscope for histopathological changes. The tissue sections were graded according to the lesion scoring system by Gamberini algorithm^4^. The relative scoring criteria of H&E staining are also listed in Table S12. To evaluate the effect of troxipide, we assessed the expression of heat shock protein 90 (HSP-90, immunoway, USA, YT5327), Cleaved-Caspase-3 (CCAS-3, immunoway, USA, YC0006YT5327) and cleaved poly (ADP-ribose) polymerase-1 (C-PARP-1, Beijing bioss biotechnology Co., Ltd) in stomach tissue sections by immunohistochemistry (IHC). The paraffin-embedded rats stomach sections were prepared by a routine procedure. The staining and the images analyses for IHC were performed according to previously described methods and our preliminary studies^93–95^. Briefly, paraffin sections (5 µm) of the rats stomach were used to analyze IHC with polyclonal antibody of HSP-90, CCAS-3 and C-PARP-1. Firstly, the paraffin sections were incubated with primary antibody at 4 °C all night. After washing, the sections were incubated with secondary antibody and the DAB kit (Bei jing bioss biotechnology Co., Ltd) was used to stain tissue sections. Then, the sections in each group (n = 10) were observed with light microscopy (ZEISS Primo Start, Germany). For each slice, at least 20 regions were randomly selected and the correspondin micrographs were captured with magnifications of 100. Subsequently, the positive staining of the images was observed using the software Image Pro Plus 6.0 (Media Cybernetics, USA). Later, the expression of proteins was demonstrated by the ratio of integral optical density (IOD). IOD = average optical density × positive area. Bar graphs in this point were performed with OriginLab Origin Pro.2019b (Northampton, MA 01060, USA).

### Data analysis

Pharmacokinetic parameters of troxipide were calculated by the pharmacokinetic software Drug and Statistics 2.1 vision (Mathematical Pharmacology Professional Committee of China, Shanghai, China). The maximum concentration (C_max_) and time to reach C_max_ (T_max_) were directly obtained from pharmacokinetic concentration–time data. The area under the concentration–time curve to the last measurable concentration point (AUC_(0−t)_) was evaluated by the linear trapezoidal rule, and the AUC_(0−∞)_ was calculated as following equation: AUC_(0−∞)_ = AUC_(0−t)_ + Ct/Ke, where the Ct was the last concentration of detectable and the Ke was a constant of terminal elimination. Moreover, the elimination half-life (t_1/2_), mean residence time (MRT), variance of residence time (VRT), elimination constant (K_10_) and absorption constant (Ka) of the drug were all determined. The absolute bioavailability (Fa) was calculated using the formula:

$$Fa(\% ) = ({\text{AUC}}_{\text{i.g.}} \times {\text{D}}_{\text{i.v.}})/({\text{AUC}}_{{\text{i.v.}}} \times {\text{D}}_{\text{i.g.}}) \times 100\%$$

where i.g. and i.v. were intragastric and intravenous of troxipide, respectively. All results of tables and figures are expressed as the mean ± standard deviation (SD). Statistical analysis was conducted by using the SPSS software version 22.0 (SPSS Inc., Chicago, IL, USA). The differences of the experiment groups were analyzed using one-factor analysis of variance (ANOVA) followed by a Tukey’s post hoc test. A significant difference was accepted for all tests at a level of *P* < 0.05 or *P* < 0.01. In additionally, all charts and figures were performed by using OriginLab Origin Pro. 2019b (Northampton, MA 01060, USA).

## Supplementary information

Supplementary Information.

## References

[1] Chen JY, Wang XD, Zhong GP, Qin XL, Li JL, Huang ZY (2011). Development and validation of a highly rapid and sensitive LC–MS/MS method for determination of SZ-685C, an investigational marine anticancer agent, in rat plasma—application to a pharmacokinetic study in rats. J. Chromatogr. B.

[2] Ineu RP, Pereira ME, Aschner M, Nogueira CW, Rocha JBT (2008). Diphenyl diselenide reverses gastric lesions in rats: involvement of oxidative stress. Food Chem. Toxicol..

[3] Shaker E, Mahmoud H, Mnaa S (2010). Anti-inflammatory and anti-ulcer activity of the extract from *Alhagi maurorum* (camelthorn). Food Chem. Toxicol..

[4] Amaral GP, Carvalho NRD, Rômulo PB, Dobrachinski F, Fachinetto R (2012). Protective action of ethanolic extract of *Rosmarinus officinalis* L. in gastric ulcer prevention induced by ethanol in rats. Food Chem. Toxicol..

[5] Leushacke M, Ng A, Galle J, Loeffler M, Barker N (2013). Lgr5+ gastric stem cells divide symmetrically to effect epithelial homeostasis in the pylorus. Cell Rep..

[6] Bianca B, Meyer TF (2011). The human gastric pathogen, helicobacter pylori, and its association with gastric cancer and ulcer disease. Ulcers.

[7] Hayakawa T, Kuwahara S, Maeda S, Tanaka K, Seki M (2007). Morphology and ultrastructure of the sympathetic celiac ganglion neurons projecting to the cardia and pylorus of the rat stomach. Auton. Neurosci..

[8] Hwang SA, Hwang IY, Jung J (2012). Dipsacussaponin C from *Dipsacus asper* reduces the risk of gastritis and gastric ulcer in rats. Food Nutr. Sci..

[9] Choi EY, Hwang HJ, Kim IH, Nam TJ (2009). Protective effects of a polysaccharide from Hizikia fusiformis against ethanol toxicity in rats. Food Chem. Toxicol..

[10] Belaiche J, Burette A, Vos MD, Louis E, Deltenre M (2002). Observational survey of NSAID-related upper gastro-intestinal adverse events in Belgium. Acta Gastroenterol..

[11] Canavese G (2004). Gastric metaplasia and small bowel ulcerogenesis in a case of ulcerative jejunitis not related to celiac disease. Int. J. Surg. Pathol..

[12] Sushi K, Niraj K, Rama DM, Uday CG (2015). Pepsinogen-II 100 ins/del gene polymorphism and its elevated circulating levels are associated with gastric cancer, particularly with Helicobacter pylori infection and intestinal metaplasia. Gastric Cancer.

[13] Li TJ, Wang S, Meng XS, Bao YR, Guan SS, Liu B (2014). Metabolomics coupled with multivariate data and pathway analysis on potential biomarkers in gastric ulcer and intervention effects of corydalis yanhusuo alkaloid. PLoS ONE.

[14] Wang L, Wang X, Zhang SL, Zhu XM, Liu YQ, Song ZJ (2017). Gastroprotective effect of palmatine against acetic acid-induced gastric ulcers in rats. J. Nat. Med..

[15] Biswas K, Bandyopadhyay U, Chattopadhyay I, Varadaraj A, Ali E, Banerjee RK (2003). A novel antioxidant and antiapoptotic role of omeprazole to block gastric ulcer through scavenging of hydroxyl radical. J. Biol. Chem..

[16] Zhang Z (2007). The risk of gastric cancer in patients with duodenal and gastric ulcer: research progresses and clinical implications. J. Gastrointest. Cancer.

[17] Nardone G, Compare D (2015). The human gastric microbiota: is it time to rethink the pathogenesis of stomach diseases?. United Eur. Gastroenterol. J..

[18] Nwidu LL, Nwafor PA (2010). Gastroprotective effects of leaf extracts of *Carpolobia lutea*(polygalaceae) G. Don. in rats. Afr. J. Biotechnol..

[19] Young OT, Ok Ahn B, Jung JE, Sang PJ, Jong PS, Wook BH (2008). Accelerated ulcer healing and resistance to ulcer recurrence with gastroprotectants in rat model of acetic acid-induced gastric ulcer. J. Clin. Biochem. Nutr..

[20] Jing S, Jiang WH, Sun W (2014). Effects of smoking on serum sod and GSH-PX activities and MDA contents in rats with gastric ulcer. Appl. Mech. Mater..

[21] Mckay DM, Wallace JL (2009). Acetic acid induced ulceration in rats is not affected by infection with hymenolepis diminuta. J. Parasitol..

[22] Tannaes T, Bukholm IK, Bukholm G (2005). High relative content of lysophospholipids of helicobacter pylorimediates increased risk for ulcer disease. FEMS Immunol. Med. Microbiol..

[23] Gao Y, Gao Y, Yin F, Wang M, Wang Z, Ye T (2014). Preparation and pharmacokinetics study on gastro-floating sustained-release tablets of troxipide. Drug Dev. Ind. Pharm..

[24] Watanabe K, Nagamatsu S, Hoshiya S, Tokunaga K, Tanaka AK (2000). The effect of a novel gastric mucosal protective agent, troxipide, on helicobacter pylori induced gastric cellular apoptosis. Gastroenterology.

[25] Dewan B, Balasubramanian A (2010). Troxipide in the management of gastritis: a randomized comparative trial in general practice. Gastroenterol. Res. Pract..

[26] Kusugami K, Ina K, Hosokawa T (2000). Troxipide, a novel antiulcer compound, has inhibitory effects on human neutrophil migration and activation induced by various stimulants. Dig. Liver Dis..

[27] Inoue M, Takahashi M, Ishibashi Y, Sueyoshi S, Kobayashi F, Matsuzawa S (2005). Effects of troxipide on the prednisolone-induced aggravation of gastric mucosal lesions occurred by HC1 in rats. Jpn. Pharmacol. Ther..

[28] Matsui H, Murata Y, Kobayashi F, Shiba R, Momo K, Kondo Y (2001). Diclofenac-induced gastric mucosal fluorescence in rats. Dig. Dis. Sci..

[29] Jagdale SC, Kamble SB, Kuchekar BS, Chabukswar AR (2014). Design and evaluation of polyox and pluronic controlled gastroretentive delivery of troxipide. J. Drug Deliv..

[30] Park BK, Kitteringham NR, Maggs JL, Pirmohamed M, Williams DP (2005). The role of metabolic activation in drug-induced hepatotoxicity. Annu. Rev. Pharmacol. Toxicol.

[31] Kumar GN, Surapaneni S (2001). Role of drug metabolism in drug discovery and development. Med. Res. Rev..

[32] Genovino J, Sames D, Hamann LG, Toure BB (2016). Accessing drug metabolites via transition-metal catalyzed C–H oxidation: liver as synthetic inspiration. Angew. Chem. Int. Ed..

[33] Mukhtar YM, Adu-Frimpong M, Xu XM, Yu J (2018). Biochemical significance of limonene and its metabolites: future prospects for designing and developing highly potent anticancer drugs. Biosci. Rep..

[34] Fura A (2006). Role of pharmacologically active metabolites in drug discovery and development. Drug Discov. Today.

[35] Shankar G, Borkar RM, Udutha S (2018). Identification and structural characterization of in vivo metabolites of balofloxacin in rat plasma, urine and feces samples using Q-TOF/LC/MS/MS: in silico toxicity studies. J. Pharm. Biomed. Anal..

[36] Wang G, Fu H, Ye W, Zheng X, Xiao J, Kang D (2016). Comprehensive characterization of the in vitroand in vivo metabolites of ziyuglycoside I in rat microsome, intestinal flora, excretion specimen and fresh tissues based on LC-Q-TOF/MS. J. Pharm. Biomed. Anal..

[37] Chen HX, Chen Y, Du P, Han FM (2007). LC–MS for identification and elucidation of the structure of in-vivo and in-vitro metabolites of atropine. Chromatographia.

[38] Elsohly MA, Gul W, Elsohly KM, Murphy TP, Madgula VLM, Khan SI (2011). Liquid chromatography-tandem mass spectrometry analysis of urine specimens for K2 (JWH-018) metabolites. J. Anal. Toxicol..

[39] Hu K, Zhao G, Fu Y, Wang S, Yuan H, Xie F (2017). Screening and identification of the main metabolites of 2-amino-9h-pyrido[2,3-b]indole (aαc) in liver microsomes and rat urine by using UPLC-Q-TOF-MS/MS. J. Chromatogr. B.

[40] Testa B (2009). Drug metabolism for the perplexed medicinal chemist. Chem. Biodivers..

[41] Li XN, Rao T, Xu YF, Hu KR, Zhu ZP, Li HF, Kang D, Shao YH, Shen BY, Yin XX, Xie L, Wang GJ, Liang Y (2018). Pharmacokinetic and pharmacodynamic evidence for developing an oral formulation of octreotide against gastric mucosal injury. Acta Pharmacol. Sin..

[42] Manocha S, Lal D, Venkataraman S (2016). Administration of H2 blockers in nsaid induced gastropathy in rats: effect on histopathological changes in gastric, hepatic and renal tissues. Arq. Gastroenterol..

[43] Borato DG, Scoparo CT, Maria-Ferreira D, Silva LM, Souza LM, Iacomini M, Werner MFP, Baggio CH (2016). Healing mechanisms of the hydroalcoholic extract and ethyl acetate fraction of green tea (*Camellia sinensis* (L.) Kuntze) on chronic gastric ulcers. Naunyn Schmiedebergs Arch. Pharmacol..

[44] Yang L, Pang Y, Moses HL (2010). TGF-β and immune cells: an important regulatory axis in the tumor microenvironment and progression. Trends Immunol..

[45] Liu J, Sun D, He J (2016). Gastroprotective effects of several H2RAs on ibuprofen-induced gastric ulcer in rats. Life Sci..

[46] Qin Z (2005). Synergistic action of famotidine and chlorpheniramine on acetic acid-induced chronic gastric ulcer in rats. World J. Gastroenterol..

[47] Ghannam S, Jérôme P, Torcy-Moquet G, Jorgensen C, Yssel H (2010). Mesenchymal stem cells inhibit human th17 cell differentiation and function and induce a t regulatory cell phenotype. J. Immunol..

[48] Matsuzaki G, Umemura M (2017). Interleukin-17 family cytokines in protective immunity against infections: role of hematopoietic cell-derived and non-hematopoietic cell-derived interleukin-17s. Microbiol. Immunol..

[49] Wang R, Hasnain SZ, Tong H, Das I, Chen CH, Oancea I (2015). Neutralizing il-23 is superior to blocking il-17 in suppressing intestinal inflammation in a spontaneous murine colitis model. Inflamm. Bowel Dis..

[50] Mus AM, Corneth O, Asmawidjaja P, Lubberts E (2011). Lack of IL-17RA signalling prevents autoimmune inflammation of the joint and give rise to a TH2-like phenotype in collagen-induced arthritis. Ann. Rheum. Dis..

[51] Arezoo S, Elham A, Gholamreza S, Ehsan M, Vahid S (2016). The effects of methanolic extract of melissa officinalis on experimental gastric ulcers in rats. Iran. Red Crescent Med. J..

[52] Schulz A, Toedt G, Zenz T, Stilgenbauer S, Lichter P, Seiffert M (2011). Inflammatory cytokines and signaling pathways are associated with survival of primary chronic lymphocytic leukemia cells in vitro: a dominant role of CCl2. Haematologica.

[53] Martín F, Thomsen LL, Brett S, Gerard C, Lipp M, Lanzavecchia A (2004). Induced recruitment of NK cells to lymph nodes provides IFN-γ for Th1 priming. Nat. Immunol..

[54] Yuan W, Dimartino SJ, Redecha PB, Ivashkiv LB, Salmon JE (2011). Systemic lupus erythematosus monocytes are less responsive to interleukin-10 in the presence of immune complexes. Arthritis Rheum..

[55] Yang T, Shi Y, Lin CJ, Yan CD, Zhang DH, Lin JY (2019). Pharmacokinetics, bioavailability and tissue distribution study of JCC-02, a novel N-methyl-d-aspartate (NMDA) receptor inhibitor, in rats by LC-MS/MS. Eur. J. Pharm. Sci..

[56] Cho SO, Lim JW, Kim KH (2010). Involvement of RAS and AP-1 inhelicobacter pylori-induced expression of COX-2 and iNOS in gastric epithelial AGS cells. Dig. Dis. Sci..

[57] Khodir AE, Atef H, Said E, Elkashef HA, Salem HA (2017). Implication of NRF2/HO-1 pathway in the coloprotective effect of coenzyme Q10 against experimentally induced ulcerative colitis. Inflammopharmacology.

[58] Walters RD, Drullinger LF, Kugel JF, Goodrich JA (2013). NFATC2 recruits CJUN homodimers to an NFAT site to synergistically activate interleukin-2 transcription. Mol. Immunol..

[59] Tai JT, Michiro O, Masaru O, Kumiko T, Makiko T, Yuko I (2010). Correlation of heat shock protein expression to gender difference in development of stress-induced gastric mucosal injury in rats. J. Clin. Biochem. Nutr..

[60] Sipponen P, Graham DY (2007). Importance of atrophic gastritis in diagnostics and prevention of gastric cancer: application of plasma biomarkers. Scand. J. Gastroenterol..

[61] Sun LP, Guo XL, Zhang Y, Chen W, Bai XL, Liu J (2009). Impact of pepsinogen c polymorphism on individual susceptibility to gastric cancer and its precancerous conditions in a northeast Chinese population. J. Cancer Res. Clin. Oncol..

[62] Sumii K (2000). What is the role of helicobacter pyloriinfection in ZES-associated peptic ulcer. J. Gastroenterol..

[63] Al Menhali A, Keeley TM, Demitrack ES, Samuelson LC (2017). Gastrin induces parathyroid hormone-like hormone expression in gastric parietal cells. Am. J. Physiol. Gastrointest. Liver Physiol..

[64] Alshaker HA, Matalka KZ (2011). IFN-γ, IL-17 and TGF-β involvement in shaping the tumor microenvironment: The significance of modulating such cytokines in treating malignant solid tumors. Cancer Cell Int..

[65] Qian Y, Li GJ, Zhu K, Suo HY, Zhao X (2013). Effects of three types of resistant starch on intestine and their gastric ulcer preventive activities in vivo. J. Korean Soc. Appl. Biol. Chem..

[66] Schumacher U, Duku M, Katoh M, Julia J, Krause WJ (2004). Histochemical similarities of mucins produced by Brunner's glands and pyloric glands: a comparative study. Anat. Rec. A Discov. Mol. Cell. Evol. Biol..

[67] Paulrayer A, Mariadhas VA, Muniappan D, Choon CK, Jong-Hoon K (2010). Helicobacter pylori eradication improves gastric histology and decreases serum gastrin, pepsinogen I and pepsinogen ii levels in patients with duodenal ulcer. J. Gastroenterol. Hepatol..

[68] Ghoshal UC, Kumar S, Krishnani N, Kumari N, Tripathi S (2012). Serological assessment of gastric intestinal metaplasia and atrophy using pepsinogen-I, pepsinogen-ii and gastrin-17 levels in a low incidence area of gastric cancer endemic for h. pylori infection. Trop. Gastroenterol..

[69] Hua YS, Xia F, Kai Z, Cun W, Xin Z, Jianquan K (2015). Shuidouchi (fermented soybean) fermented in, different vessels attenuates hcl/ethanol-induced gastric mucosal injury. Molecules.

[70] Kumar S, Kumari N, Mittal RD (2015). Pepsinogen-II 100 bp ins/del gene polymorphism and its elevated circulating levels are associated with gastric cancer, particularly with Helicobacter pylori infection and intestinal metaplasia. Gastric Cancer.

[71] Yi R, Wang R, Sun P, Zhao X (2015). Antioxidant-mediated preventative effect of dragon-pearl tea crude polyphenol extract on reserpine-induced gastric ulcers. Exp. Ther. Med..

[72] Depoortere I, Thijs T, Janssen S, Smet BD, Tack J (2010). Colitis affects the smooth muscle and neural response to motilin in the rabbit antrum. Br. J. Pharmacol..

[73] Huang XR, Hui CWC, Chen YX, Chun B, Wong Y, Fung PCW (2001). Macrophage migration inhibitory factor is an important mediator in the pathogenesis of gastric inflammation in rats. Gastroenterology.

[74] Mondal A, Bennett LL (2016). Resveratrol enhances the efficacy of sorafenib mediated apoptosis in human breast cancer mcf7 cells through ros, cell cycle inhibition, caspase 3 and parp cleavage. Biomed. Pharmacother..

[75] Messaoudi S, Peyrat JF, Brion JD, Alami M (2011). Heat-shock protein 90 inhibitors as antitumor agents: a survey of the literature from 2005 to 2010. Expert Opin. Ther. Pat..

[76] Kozeko YL (2014). Changes in heat-shock protein synthesis and thermotolerance of arabodopsis thaliana seedlings resulting from hsp90 inhibition by geldanamycin. Cell Tissue Biol..

[77] Lyall EG, Blott M, Ruiter AD, Hawkins D, Taylor GP (2010). Guidelines for the management of HIV infection in pregnant women and the prevention of mother to child transmission. HIV Med..

[78] Shah F, Huang J, Cui K, Nie L, Wang K (2011). Impact of high-temperature stress on rice plant and its traits related to tolerance. J. Agric. Sci..

[79] Wang H, Lu C, Tan Y, Xie J, Jiang J (2014). Effect of adriamycin on brca1 and parp-1 expression in MCF-7 breast cancer cells. Int. J. Clin. Exp. Pathol..

[80] Yang JS, Ramanathan MP, Muthumani K, Choo AY, Jin SH, Yu QC (2002). Induction of inflammation by west nile virus capsid through the caspase-9 apoptotic pathway. Emerg. Infect. Dis..

[81] Tu Z, Chu W, Zhang J, Dence CS, Welch MJ, Mach RH (2005). Synthesis and in vivo evaluation of 11CPJ34, a potential radiotracer for imaging the role of PARP-1 in necrosis. Nucl. Med. Biol..

[82] Wang HH, Song YX, Hao DJ, Bai M, Jin L, Gu J (2014). Ultrasound-targeted microbubble destruction combined with dual targeting of HSP72 and HSC70 inhibits HSP90 function and induces extensive tumor-specific apoptosis. Int. J. Oncol..

[83] Ghafari F, Gutierrez CG, Hartshorne GM (2007). Apoptosis in mouse fetal and neonatal oocytes during meiotic prophase one. BMC Dev. Biol..

[84] Liu W, Yang ML, Chen XJ, Li L, Zhou AJ, Chen SH (2018). Mechanisms of antiulcer effect of an active ingredient group of modified Xiao Chaihu decoction. Evid. Based Complement. Altern. Med..

[85] Kandhare AD, Bodhankar SL, Mohan V, Thakurdesai PA (2015). Pharmacokinetics, tissue distribution and excretion study of a furostanol glycoside-based standardized fenugreek seed extract in rats. Ren. Fail..

[86] Xu H, Feng C, Cao YY, Lu Y, Xi J, Ji JY, Lu DS, Zhang XY, Luan Y (2019). Distribution of the parent compound and its metabolites in serum, urine, and feces of mice administered 2,20,4,40-tetrabromodiphenyl ether. Chemosphere.

[87] Diao Z, Li J, Liu Q, Wang Y (2018). In-vivo metabolite profiling of chicoric acid in rat plasma, urine and feces after oral administration using liquid chromatography quadrupole time of flight mass spectrometry. J. Chromatogr. B.

[88] Qin ZF, Dai Y, Yao ZH, He LL, Wang QY, Geng JL (2016). Study on chemical profiles and metabolites of allii macrostemonis bulbus as well as its representative steroidal saponins in rats by ultra-performance liquid chromatography coupled with quadrupole time-of-flight tandem mass spectrometry. Food Chem..

[89] Afzal A, Zhong YX, Sarfraz M, Peng Y, Sheng LS, Wu ZM, Sun JG, Wang GJ (2016). Identification and characterization of in vivo metabolites of asulacrine using advanced mass spectrophotometry technique in combination with improved data mining strategy. J. Chromatogr. A.

[90] Wei HM, Shang LY, Zhan CG, Zheng F (2020). Effects of cebranopadol on cocaine-induced hyperactivity and cocaine pharmacokinetics in rats. Sci. Rep..

[91] Manvich DF, Webster KA, Foster SL, Farrell MS, Ritchie JC, Porter JH, Weinshenker D (2018). The DREADD agonist clozapine N-oxide (CNO) is reverse-metabolized to clozapine and produces clozapine-like interoceptive stimulus effects in rats and mice. Sci. Rep..

[92] Zeng X, Su W, Zheng Y, He YD, He Y, Rao HY, Peng W, Yao HL (2019). Pharmacokinetics, tissue distribution, metabolism, and excretion of naringin in aged rats. Front. Pharmacol..

[93] Alzokaky AA, Abdelkader EM, El-Dessouki AM, Khaleel SA, Raslan NA (2020). C-phycocyanin protects against ethanol-induced gastric ulcers in rats: role of HMGB1/NLRP3/NF-κB pathway. Basic Clin Pharmacol Toxicol.

[94] Piao XH, Li SD, Sui XD, Guo LY, Liu XM, Li HM, Gao LM, Cai SS, Li YR, Wang TT, Liu BH (2018). 1-Deoxynojirimycin (DNJ) ameliorates indomethacin-indu gastric ulcer in mice by affecti NF-kappaB signaling pathway. Front. Pharmacol..

[95] Liang JL, Dou YX, Wu X, Li HL, Wu JZ, Huang QH, Luo DD, Yi TG, Liu YH, Su ZR, Chen JP (2018). Prophylactic efficacy of patchoulene epoxide against ethanol-induced gastric ulcer in rats: Influence on oxidative stress, inflammation and apoptosis. Chem. Biol. Interact..

