# Supplementary material for: Metabolites profiling and pharmacokinetics of troxipide and its pharmacodynamics in rats with gastric ulcer
Source: Sci Rep. 2020 Aug 12;10:13619. doi: 10.1038/s41598-020-70312-7 (PMC7423950; doi:10.1038/s41598-020-70312-7)
Supplement: Supplementary file 1 — Supplementary Information. [file 41598_2020_70312_MOESM1_ESM.pdf]

# Supplementary Information

Metabolites profiling and pharmacokinetics of troxipide and its pharmacodynamics in rats with gastric ulcer

*Hongbin Guo, Baohua Chen, Zihan Yan, Jian Gao, Jiamei Tang, Chengyan Zhou**

*College of Pharmaceutical Sciences, Institute of Life Science and Green Development, Key Laboratory of Pharmaceutical Quality Control of Hebei Province, Hebei University, Baoding, 180 WuSi Road, Lianchi District, 071002, China*

**Corresponding author at: College of Pharmaceutical Sciences, Institute of Life Science and Green Development, Key Laboratory of Pharmaceutical Quality Control of Hebei Province, Hebei University, Baoding, 180 WuSi Road, Lianchi District, 071002, China*

*Tel/Fax: +86 312 5971107; E-mail address: xuefanone@163.com (C.Y. Zhou).*

**Table S1 The calibration curve of plasma and tissue.**

**Table S2 Precision and accuracy of troxipide in rat plasma and stomach (x ± s, ng/mL).**

**Table S3 Extraction recovery of troxipide in rat plasma and tissue.**

**Table S4 Matrix effects of troxipide in rat plasma and tissue.**

**Table S5 Stability of troxipide in rat plasma and Stomach (x ± s, ng/mL).**

**Table S6 The dilution integrity of troxipide in rat plasma (x ± s, ng/mL).**

**Table S7 The instrument carryover of troxipide in rat plasma (x ± s, ng/mL).**

**Table S8 The rats body weights record at the end of the experiment (x ± s, g).**

**Table S9 The rats food intake record in at the end of the experiment (x ± s, g).**

**Table S10 The rats water intake record at the end of the experiment (x ± s, mL).**

**Table S11 The Ulcer Area and the Ulcer Inhibition in rats.**

**Table S12 The scoring criteria of ulcer index and HE staining in stomach .**

**Figure S1. The possible fragmentations pattern of the troxipide.**

**Figure S2. LC-MS/MS spectra of troxipide metabolites (M0-M45).**

**Figure S3. Representative SRM chromatograms of troxipide and IS in the rat plasma and tissues.** (A) a blank plasma and all tissues sample; (B) a blank plasma and all tissue sample spiked with troxipide (LLOQ, 5 ng/mL); (C) plasma and all tissue sample 60 min after administration of single dosage 40 mg/kg.

**Figure S4 A linear relationship between drug $C_{max}$ to dose in vivo in rats.**

**Figure S5 A linear relationship between drug $AUC_{(0-t)}$ to dose in vivo in rats.**

**Figure S6 A linear relationship between drug $AUC_{(0-\infty)}$ to dose in vivo in rats.**

**Figure S7. MTL, GAS, TNF-α, PG-I , PG-II, IL-17, IFN-γ, AP-1 and IL-6 levels in the rat plasma before troxipide treatment.** NCG, normal group (0.9% normal saline 10 mL kg-1 day-1); GUG, gastric ulcer group (5% acetic acid 10 mL kg-1 day-1). Values are presented as means ± SD for all groups (n = 10). *P < 0.05 and **P < 0.01 indicate statistically significant differences when the GUG is compared with the NCG.

**Figure S8. Macroscopic and microscopic analysis of stomach tissue before troxipide treatment.** (A) Macroscopic analysis of GU before troxipide treatment. (B) Sections of the gastric mucosa (H&E staining) (100×). (C) Sections of the gastric mucosa (H&E staining) (400×). NCG, normal group (0.9% normal saline 10 mL kg-1 day-1); GUG, gastric ulcer group (5% acetic acid 10 mL kg-1 day-1).

## Supplementary Results and Discussion

Metabolic study: Identification of troxipide metabolites in feces; Identification of troxipide metabolites in urine

Pharmacokinetics and tissue distribution study: Method validation

## Supplementary Materials and Methods

Chemicals and reagents

Sample preparation

Method validation

**Table S1 The calibration curve of plasma and tissue.**

| Analytes | y=ax+b | R2 |
|---|---|---|
| Plasma | y=0.0035x+0.0029 | 0.9999 |
| Heart | y=0.0039x+0.0225 | 0.9999 |
| Liver | y=0.0031x+0.0489 | 0.9999 |
| Spleen | y=0.0035x+0.0045 | 0.9999 |
| Lung | y=0.0047x+0.1664 | 0.9999 |
| Kidney | y=0.0207x+0.7752 | 0.9999 |
| Brain | y=0.0195+0.1174 | 0.9999 |
| Stomach | y=0.0126x+0.1917 | 0.9999 |
| Pancreas | y=0.0065x+0.2015 | 0.9999 |
| Intestinal | y=0.0067x+0.1528 | 0.9999 |

**Table S2 Precision and accuracy of troxipide in rat plasma and stomach (x ± s, ng/mL).**

| Concentration | | | Mean ± SD | Intra-day | | Inter-day | |
|---|---|---|---|---|---|---|---|
| | | | | RSD(%) | RE(%) | RSD(%) | RE(%) |
| Plasma | 15 | Day1 | 15.29 ± 0.54 | 3.6 | 2.0 | 1.8 | 0.2 |
| | | Day2 | 15.05 ± 0.03 | 5.4 | 0.4 | | |
| | | Day3 | 14.76 ± 0.85 | 5.8 | -1.6 | | |
| | 400 | Day1 | 420.07 ± 20.35 | 4.8 | 5.0 | 1.8 | 3.0 |
| | | Day2 | 410.18 ± 16.65 | 4.1 | 2.5 | | |
| | | Day3 | 405.71 ± 10.31 | 2.5 | 1.4 | | |
| | 8000 | Day1 | 8381.56 ± 376.90 | 5.6 | -4.3 | 4.8 | 1.1 |
| | | Day2 | 8250.54 ± 381.42 | 4.4 | 4.7 | | |
| | | Day3 | 7648.20 ± 433.14 | 4.6 | 3.1 | | |
| Stomach | 15 | Day1 | 15.51 ± 1.55 | 10.0 | 3.4 | 4.3 | 4.4 |
| | | Day2 | 16.41 ± 2.26 | 13.8 | 9.4 | | |
| | | Day3 | 15.07 ± 1.17 | 7.8 | 0.5 | | |
| | 400 | Day1 | 424.75 ± 30.65 | 7.2 | 6.2 | 2.1 | 4.9 |
| | | Day2 | 409.53 ± 19.14 | 4.7 | 2.4 | | |
| | | Day3 | 424.62 ± 12.14 | 2.9 | 6.2 | | |
| | 8000 | Day1 | 7643.76 ± 623.82 | 8.2 | -4.5 | 4.2 | 0.3 |
| | | Day2 | 8047.84 ± 599.26 | 7.4 | 0.6 | | |
| | | Day3 | 8317.34 ± 293.58 | 3.5 | 4.0 | | |

**Table S3 Extraction recovery of troxipide in rat plasma and tissue.**

| | 15 | | | 400 | | | 8000 | | |
|---|---|---|---|---|---|---|---|---|---|
| | Mean±SD (%) | RSD(%) | RE(%) | Mean(%) | RSD(%) | RE(%） | Mean(%) | RSD(%) | RE(%) |
| Plasma | 103.28 ± 4.49 | 4.35 | 4.49 | 108.37±8.43 | 7.78 | 8.37 | 94.43 ± 7.85 | 8.32 | -5.57 |
| Heart | 102.60 ± 5.60 | 5.45 | 5.60 | 105.30 ± 12.65 | 12.01 | 5.30 | 93.22 ± 5.86 | 6.29 | -6.79 |
| Liver | 101.00 ± 5.65 | 5.59 | 5.65 | 102.08 ± 8.35 | 8.18 | 2.08 | 98.04 ± 9.50 | 9.68 | -1.96 |
| Spleen | 98.00 ± 1.45 | 1.48 | 1.45 | 103.57 ± 9.58 | 9.25 | 3.57 | 99.76 ± 6.83 | 6.85 | -0.23 |
| Lung | 102.92 ± 7.14 | 6.94 | 7.14 | 110.00 ± 1.84 | 1.67 | 10.01 | 95.98± 9.62 | 10.03 | -4.02 |
| Kidney | 101.85 ± 11.05 | 8.91 | 9.00 | 108.99 ± 3.47 | 3.18 | 8.99 | 92.10 ± 5.70 | 7.22 | -12.74 |
| Brain | 98.35 ± 11.23 | 11.42 | 11.23 | 103.99 ± 8.50 | 8.18 | 3.99 | 90.64 ± 3.33 | 7.72 | -12.42 |
| Stomach | 92.50 ±2.07 | 2.23 | 2.07 | 100.67 ± 5.19 | 5.16 | 6.68 | 96.30 ± 10.23 | 14.31 | -5.98 |
| Intestinal | 92.31 ±1.91 | 2.07 | 1.91 | 101.96 ± 7.02 | 6.89 | 1.97 | 101.72 ± 6.91 | 6.80 | 1.72 |
| Pancreas | 95.17 ±3.07 | 3.22 | 3.07 | 105.41 ± 3.56 | 3.37 | 5.42 | 101.86 ± 6.67 | 6.55 | 1.87 |

**Table S4 matrix effects of troxipide in rat plasma and tissue.**

| | 15 | | | 400 | | | 8000 | | |
|---|---|---|---|---|---|---|---|---|---|
| | Mean±SD (%) | RSD(%) | RE(%) | Mean(%) | RSD(%) | RE（%） | Mean(%) | RSD(%) | RE(%) |
| Plasma | 106.39 ± 5.71 | 5.37 | 6.39 | 98.54 ± 3.41 | 3.46 | -1.46 | 95.65 ± 9.53 | 9.96 | -4.35 |
| Heart | 101.82 ± 8.41 | 8.26 | 1.82 | 98.61± 3.50 | 3.56 | -1.39 | 99.06 ± 4.54 | 4.58 | -0.94 |
| Liver | 100.78 ± 6.74 | 6.69 | 0.78 | 99.18 ± 2.64 | 2.67 | -0.82 | 96.57 ± 7.20 | 7.45 | -3.43 |
| Spleen | 100.95 ± 6.72 | 6.66 | 0.95 | 103.57 ± 9.58 | 4.90 | -2.99 | 91.66 ± 3.43 | 3.74 | -8.34 |
| Lung | 99.83 ± 8.41 | 8.43 | -0.17 | 110.00 ± 1.84 | 3.56 | -6.93 | 93.44 ± 6.51 | 6.97 | 6.56 |
| Kidney | 95.01 ± 4.89 | 5.15 | -4.99 | 108.99 ± 3.47 | 3.49 | -6.96 | 100.43 ± 10.71 | 10.67 | 0.43 |
| Brain | 93.00 ± 17.24 | 1.85 | -7.00 | 103.99 ± 8.50 | 4.35 | -5.17 | 102.80 ± 7.33 | 7.13 | 2.80 |
| Stomach | 92.50 ± 2.07 | 2.23 | -7.50 | 100.67 ± 5.19 | 1.85 | -3.78 | 99.49 ± 10.24 | 10.29 | -0.51 |
| Intestinal | 92.31 ± 1.91 | 2.07 | -7.69 | 101.96 ± 7.02 | 2.11 | -3.32 | 95.05 ± 3.51 | 3.69 | -4.95 |
| Pancreas | 95.17 ± 3.42 | 3.59 | -4.83 | 105.41 ± 3.56 | 2.02 | -4.31 | 95.44 ± 3.81 | 4.00 | -4.56 |

**Table S5 Stability of troxipide in rat plasma and Stomach (x ± s, ng/mL).**

| | Plasma | | | | Stomach | | | |
|---|---|---|---|---|---|---|---|---|
| | Concentration (ng/mL) | | | | Concentration (ng/mL) | | | |
| | Nominal | Found | RSD(%) | RE(%) | Nominal | Found | RSD(%) | RE(%) |
| room temperature (4H) | 15 | 15.66± 1.34 | 8.5 | 4.4 | 15 | 14.32 ± 0.84 | 5.8 | -4.5 |
| | 400 | 389.90 ± 20.90 | 5.3 | -2.5 | 400 | 407.05 ±33.12 | 8.1 | 1.8 |
| | 8000 | 8395.40 ± 402.58 | 4.8 | 4.9 | 8000 | 7935.956±342.258 | 4.3 | -0.8 |
| auto-sampler (24H) | 15 | 15.43 ± 0.85 | 5.5 | 2.8 | 15 | 15.69 ± 1.25 | 7.9 | 3.6 |
| | 400 | 379.27 ± 17.59 | 4.6 | -5.2 | 400 | 39.58±13.26 | 3.4 | -1.0 |
| | 8000 | 8123.76 ± 344.58 | 4.2 | 1.5 | 8000 | 8350.76±517.40 | 6.2 | 4.4 |
| Three freeze–thaw | 15 | 14.87 ± 0.58 | 4.0 | -0.9 | 15 | 14.64 ± 0.74 | 5.1 | -0.02 |
| | 400 | 385.62 ± 24.60 | 6.4 | -3.6 | 400 | 404.07±11.67 | 2.9 | 1.0 |
| | 8000 | 8166.72 ± 318.06 | 3.9 | 2.1 | 8000 | 8077.28±166.10 | 2.1 | 0.1 |
| −80°C, 30 days | 15 | 15.40 ± 0.86 | 5.6 | 2.7 | 15 | 15.58 ± 0.97 | 6.3 | 3.9 |
| | 400 | 389.82 ± 18.55 | 4.8 | -2.5 | 400 | 377.64±23.99 | 6.3 | -0.6 |
| | 8000 | 7985.42 ± 270.27 | 3.4 | -0.1 | 8000 | 7761.92±387.84 | 5.0 | -2.9 |

**Table S6 The dilution integrity of troxipide in rat plasma (x ± s, ng/mL).**

| Concentration (ng/mL) | Observed concentration (mean ± SD, ng/mL) | RSD(%) | RE(%) |
|---|---|---|---|
| 15 | 14.620 ± 0.563 | 3.9 | -2.5 |
| 2000 | 1910.792 ± 130.280 | 6.9 | -4.1 |
| 100000 | 104000.588 ± 7740.264 | 7.4 | 4.0 |

**Table S7 The instrument carryover of troxipide in rat plasma (x ± s, ng/mL).**

| Concentration | Observed concentration | RSD(%) | RE(%) |
|---|---|---|---|
| 0 | 0.185 ± 0.007 | 6.7 | 8.4 |
| 8000 | 8731.110 ± 504.706 | 5.8 | 9.1 |

**Table S8 The rats body weights record at the end of the experiment (x ± s, g).**

| | 0 days | 4 days | 8 days | 12 days | 16 days | 20 days | 24 days | 28 days |
|---|---|---|---|---|---|---|---|---|
| NC | 201.31±20.17 | 227.42±16.04 | 250.07±15.86 | 267.54±15.60 | 288.18±15.90 | 307.28±14.74 | 327.89±13.26 | 345.06±15.44 |
| GUG | 203.54±24.47 | 220.89±22.23 | 235.53±18.12$^{*}$ | 247.50±17.40$^{**}$ | 256.45±17.26$^{**}$ | 265.02±14.31$^{**}$ | 274.27±13.99$^{**}$ | 282.00±13.83$^{**}$ |
| THG | 200.54±21.77 | 220.40±21.28 | 237.75±19.63$^{\#}$ | 245.39±19.80$^{\#\#}$ | 260.84±21.06$^{\#\#}$ | 288.20±18.95$^{\#\#}$ | 312.34±16.70$^{\#}$ | 340.19±14.92 |
| TMG | 205.30±23.78 | 218.53±20.19 | 234.50±19.55$^{\#}$ | 246.95±19.73$^{\#\#}$ | 260.30±19.16$^{\#\#}$ | 280.43±16.94$^{\#\#}$ | 305.19±14.79$^{\#\#}$ | 321.50±11.74$^{\#}$ |
| TLG | 206.49±24.49 | 217.74±20.88 | 237.58±20.31$^{\#}$ | 249.86±18.27$^{\#\#}$ | 258.36±17.33$^{\#\#}$ | 263.93±16.06$^{\#\#}$ | 285.75±16.33$^{\#\#}$ | 308.92±15.28$^{\#}$ |

**Table S9 The rats food intake record at the end of the experiment (x ± s, g).**

| | 4 days | 8 days | 12 days | 16 days | 20 days | 24 days | 28 days |
|---|---|---|---|---|---|---|---|
| NC | 23.37±2.09 | 25.52±2.50 | 29.47±3.23 | 30.20±4.98 | 33.32±3.76 | 34.21±3.86 | 36.25±5.07 |
| GUG | 17.73±1.92$^{**}$ | 20.59.±1.90$^{**}$ | 22.74±3.34$^{**}$ | 24.11±3.38$^{**}$ | 23.35±2.36$^{**}$ | 21.34±2.86$^{**}$ | 20.58±4.50$^{**}$ |
| THG | 16.17±2.90$^{\#\#}$ | 19.31±2.45$^{\#\#}$ | 21.05±2.34$^{\#\#}$ | 24.28±2.35$^{\#\#}$ | 28.58±1.28$^{\#\#}$ | 32.80±3.45$^{\#}$ | 34.84±3.67 |
| TMG | 17.59±3.38$^{\#\#}$ | 21.18±1.58$^{\#\#}$ | 22.02±2.16$^{\#\#}$ | 25.42±2.14$^{\#\#}$ | 27.46±3.29$^{\#\#}$ | 30.83±3.50$^{\#}$ | 31.56±6.06$^{\#}$ |
| TLG | 16.59±2.38$^{\#\#}$ | 20.09±2.55$^{\#\#}$ | 21.16±2.30$^{\#\#}$ | 23.44±2.56$^{\#\#}$ | 24.22±2.46$^{\#\#}$ | 25.05±3.53$^{\#\#}$ | 26.02±4.24$^{\#\#}$ |

**Table S10 The rats water intake record at the end of the experiment (x ± s, mL).**

| | 4 days | 8 days | 12 days | 16 days | 20 days | 24 days | 28 days |
|---|---|---|---|---|---|---|---|
| NC | 16.32±2.06 | 20.32±2.48 | 21.13±2.55 | 23.02±2.45 | 25.19±3.57 | 26.38±3.85 | 28.09±2.57 |
| GUG | 24.37±2.41$^{**}$ | 26.05±2.17$^{**}$ | 28.58±2.56$^{**}$ | 29.87±3.43$^{**}$ | 32.17±4.27$^{**}$ | 34.09±3.16$^{**}$ | 37.01±3.56$^{**}$ |
| THG | 25.23±1.56$^{\#\#}$ | 26.24±2.18$^{\#\#}$ | 28.47±2.31$^{\#\#}$ | 28.10±3.89$^{\#\#}$ | 29.55±4.50$^{\#\#}$ | 30.41±3.58$^{\#\#}$ | 31.40±4.81$^{\#\#}$ |
| TMG | 26.98±2.37$^{\#\#}$ | 27.02±3.77$^{\#\#}$ | 29.48±3.91$^{\#\#}$ | 29.57±3.15$^{\#\#}$ | 30.16±3.56$^{\#\#}$ | 31.30±4.14$^{\#\#}$ | 33.53±4.50$^{\#\#}$ |
| TLG | 25.18±2.14$^{\#\#}$ | 28.14±3.49$^{\#\#}$ | 30.31±3.05$^{\#\#}$ | 30.65±4.15$^{\#\#}$ | 31.31±4.47$^{\#\#}$ | 32.70±3.93$^{\#\#}$ | 34.28±4.48$^{\#\#}$ |

**Table S11 The Ulcer Area and the Ulcer Inhibition in rats.**

| | Ulcer Area($mm^2$) | Ulcer Inhibition (%) |
|---|---|---|
| NC | 0 | 100 |
| GUG | 19.243 ± 1.771 | 0 |
| THG | 2.053 ± 0.407 | 89.3 |
| TMG | 5.93 ± 0.717 | 69.2 |
| TLG | 10.82 ± 1.189 | 43.8 |

**Table S12 The scoring criteria of ulcer index and HE staining in stomach .**

| | **scoring criteria** | **score** |
|---|---|---|
| Ulcer index | normal stomachs | 0 |
| | loss of mucosal folding | +1 |
| | mucosal discoloration | +1 |
| | edema | +1 |
| | hemorrhage | +1 |
| | ulcers/$cm^2$ less than 1 mm | +2 |
| | ulcers more than 1 mm/$cm^2$ | +3 |
| | perforated ulcers | +4 |
| H&E staining | normal stomachs | 0 |
| | mild damage (edema ) | +1 |
| | Moderate damage (inflammatory infiltration) | +2 |
| | usevere damage (degeneration and necrosis) | +3 |

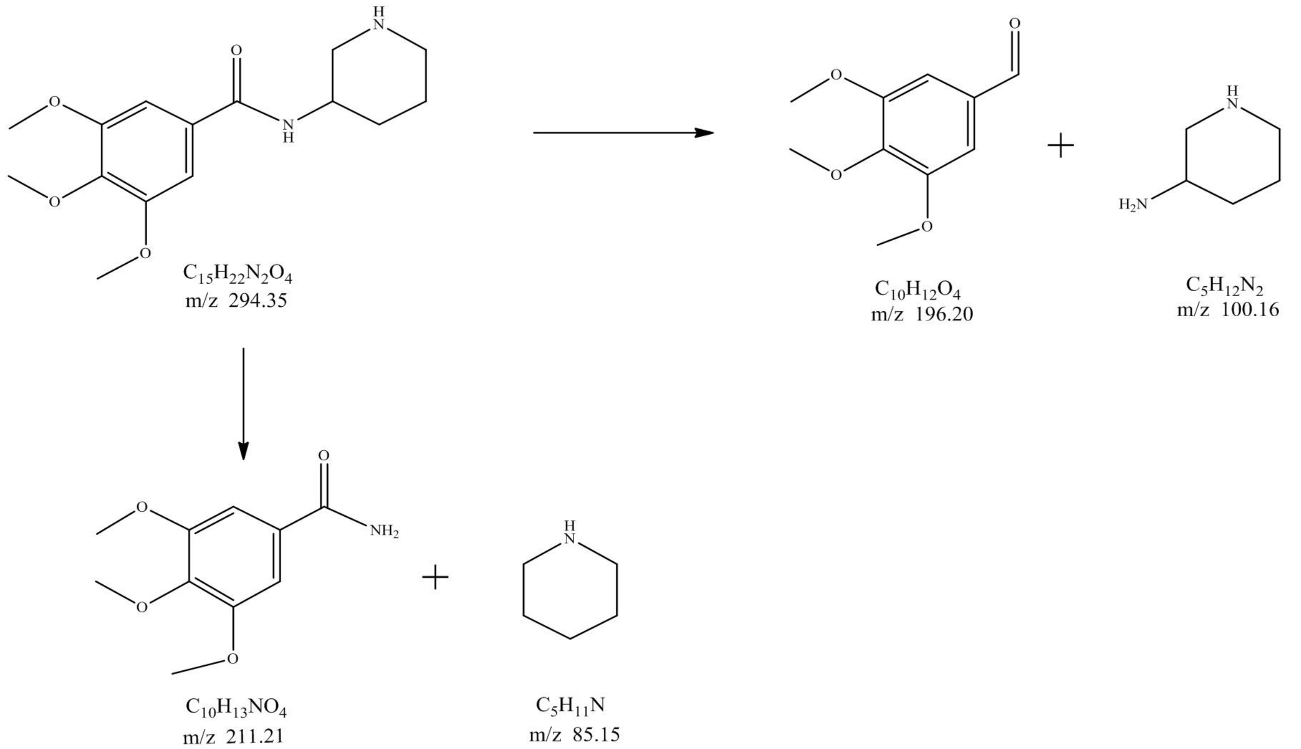


**Figure S1. The possible fragmentations pattern of the troxipide.**

M0

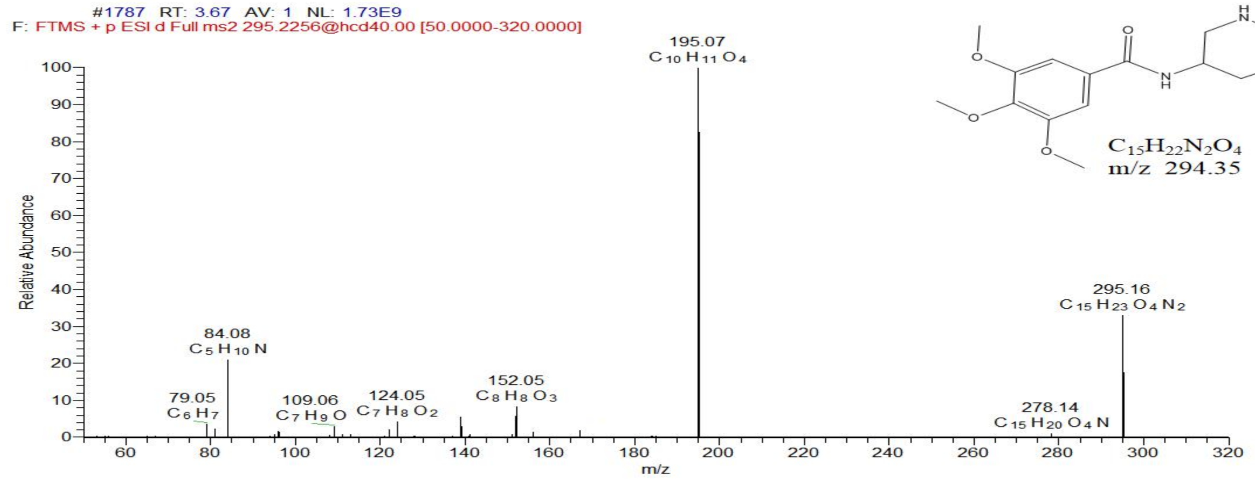
#1787 RT: 3.67 AV: 1 NL: 1.73E9
F: FTMS + p ESI d Full ms2 295.2256@hcd40.00 [50.0000-320.0000]
Relative Abundance
m/z
195.07 C10 H11 O4
295.16 C15 H23 O4 N2
278.14 C15 H20 O4 N
84.08 C5 H10 N
79.05 C6 H7
109.06 C7 H9 O
124.05 C7 H8 O2
152.05 C8 H8 O3
C15H22N2O4
m/z 294.35

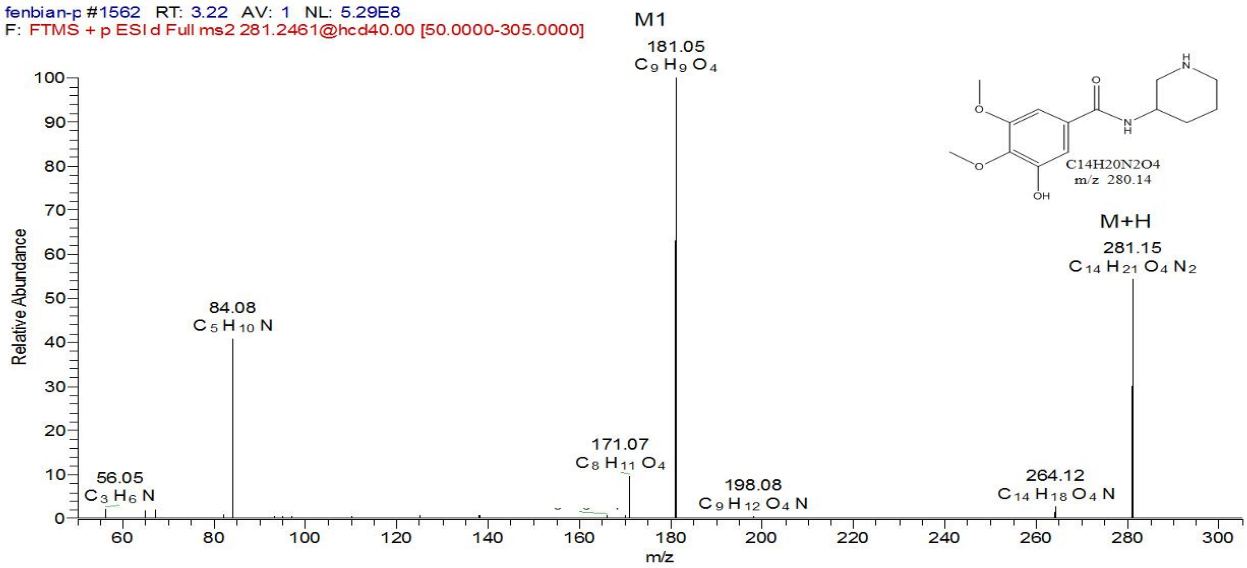
fenbian-p #1562 RT: 3.22 AV: 1 NL: 5.29E8
F: FTMS + p ESI d Full ms2 281.2461@hcd40.00 [50.0000-305.0000]
M1
181.05 C9 H9 O4
C14H20N2O4
m/z 280.14
M+H
281.15 C14 H21 O4 N2
84.08 C5 H10 N
56.05 C3 H6 N
171.07 C8 H11 O4
198.08 C9 H12 O4 N
264.12 C14 H18 O4 N
Relative Abundance
m/z

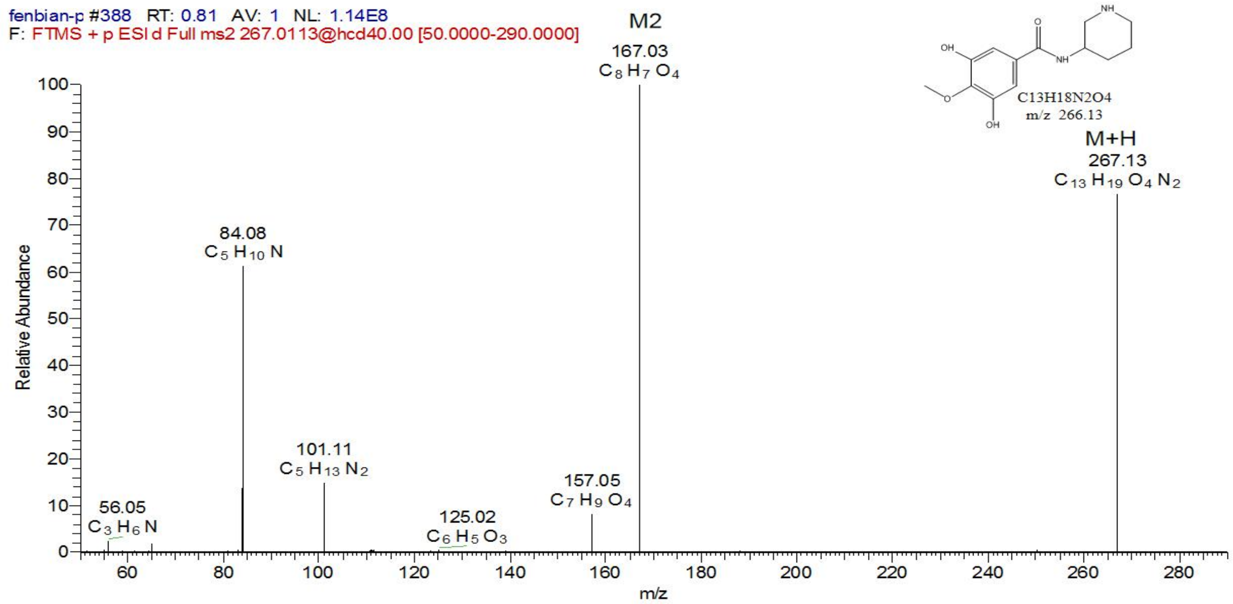
fenbian-p #388 RT: 0.81 AV: 1 NL: 1.14E8
F: FTMS + p ESI d Full ms2 267.0113@hcd40.00 [50.0000-290.0000]
M2
167.03 C8 H7 O4
C13H18N2O4
m/z 266.13
M+H
267.13 C13 H19 O4 N2
84.08 C5 H10 N
101.11 C5 H13 N2
157.05 C7 H9 O4
125.02 C6 H5 O3
56.05 C3 H6 N
Relative Abundance
m/z

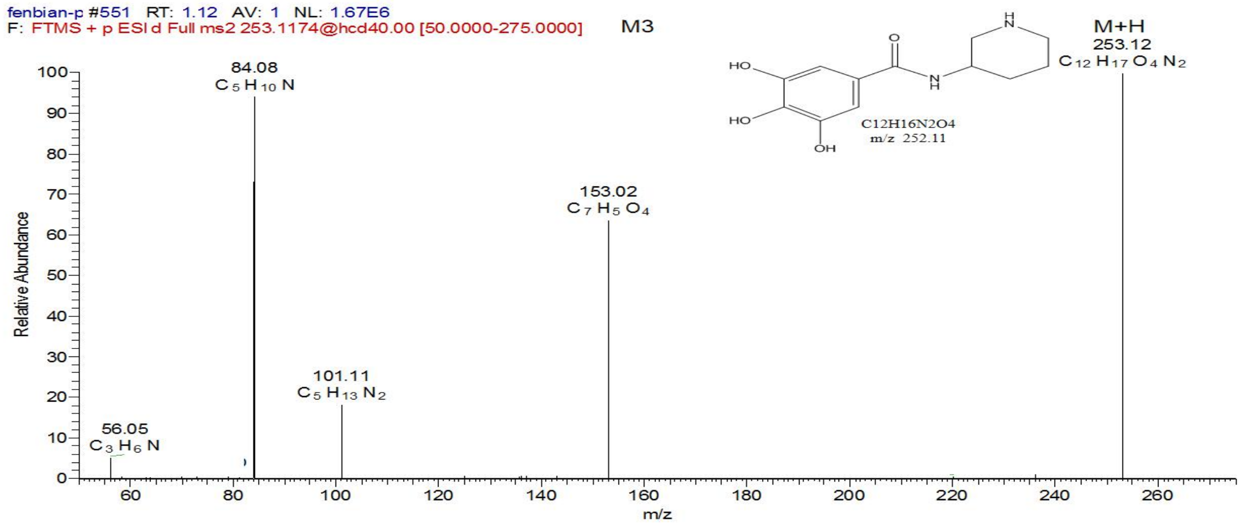
fenbian-p #551 RT: 1.12 AV: 1 NL: 1.67E6
F: FTMS + p ESI d Full ms2 253.1174@hcd40.00 [50.0000-275.0000]
M3
M+H
253.12
C 12 H 17 O 4 N 2
84.08
C 5 H 10 N
153.02
C 7 H 5 O 4
101.11
C 5 H 13 N 2
56.05
C 3 H 6 N
C12H16N2O4
m/z 252.11
Relative Abundance
m/z

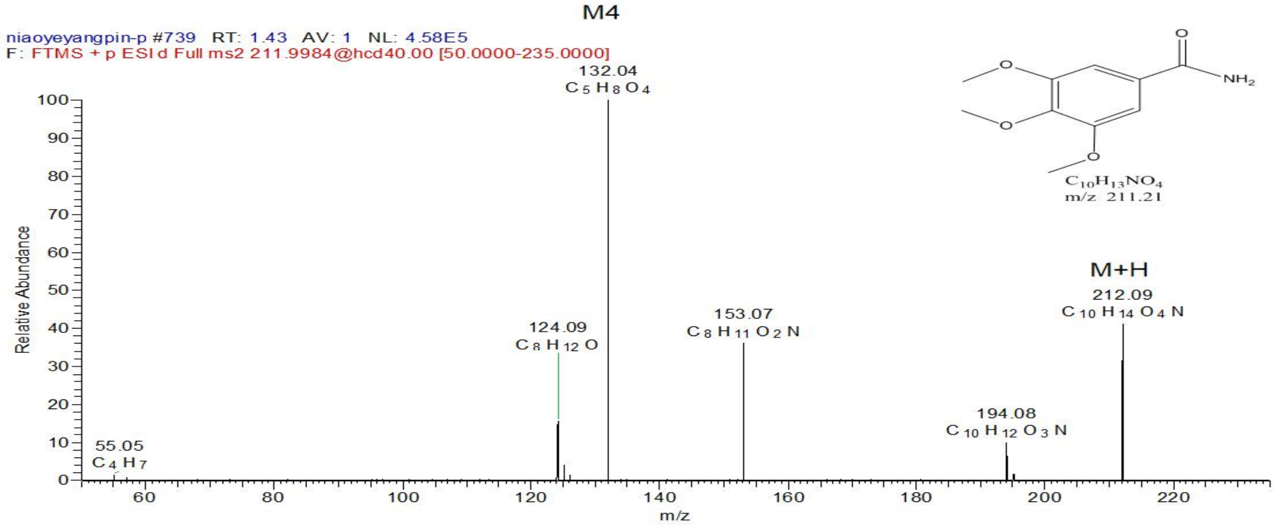
M4
niaoyeyangpin-p #739 RT: 1.43 AV: 1 NL: 4.58E5
F: FTMS + p ESI d Full ms2 211.9984@hcd40.00 [50.0000-235.0000]
132.04
C 5 H 8 O 4
124.09
C 8 H 12 O
153.07
C 8 H 11 O 2 N
M+H
212.09
C 10 H 14 O 4 N
194.08
C 10 H 12 O 3 N
55.05
C 4 H 7
C10H13NO4
m/z 211.21
Relative Abundance
m/z

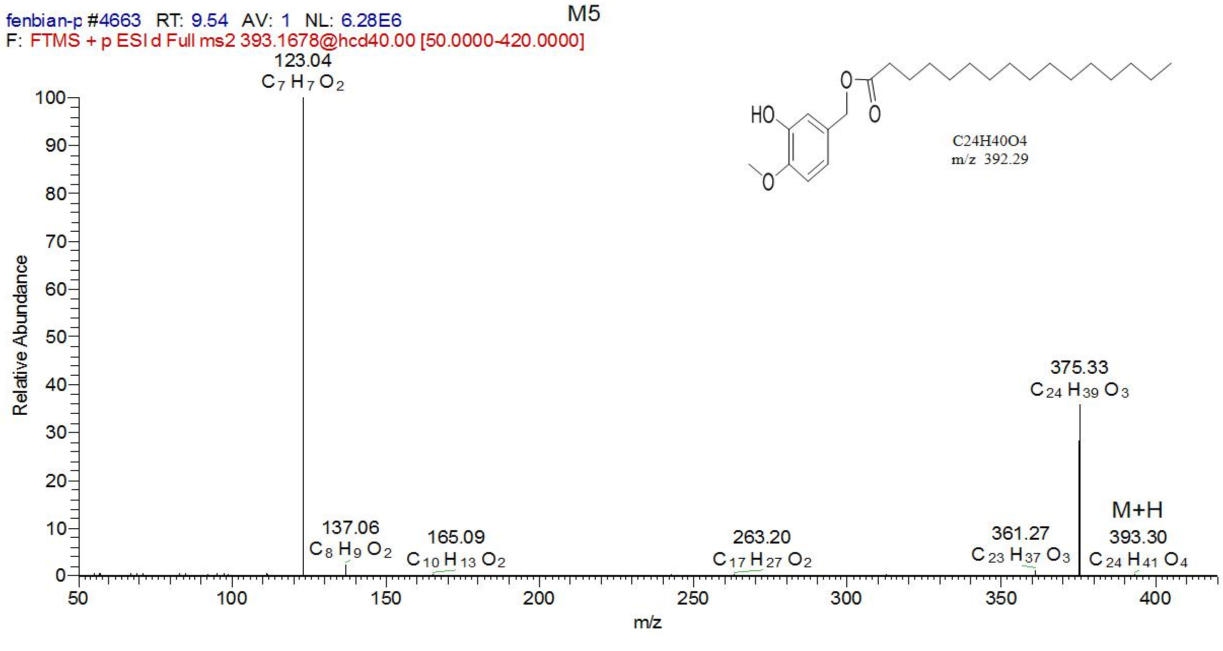
fenbian-p #4663 RT: 9.54 AV: 1 NL: 6.28E6
M5
F: FTMS + p ESI d Full ms2 393.1678@hcd40.00 [50.0000-420.0000]
123.04
C 7 H 7 O 2
C24H40O4
m/z 392.29
375.33
C 24 H 39 O 3
137.06
C 8 H 9 O 2
165.09
C 10 H 13 O 2
263.20
C 17 H 27 O 2
361.27
C 23 H 37 O 3
M+H
393.30
C 24 H 41 O 4
Relative Abundance
m/z

M6

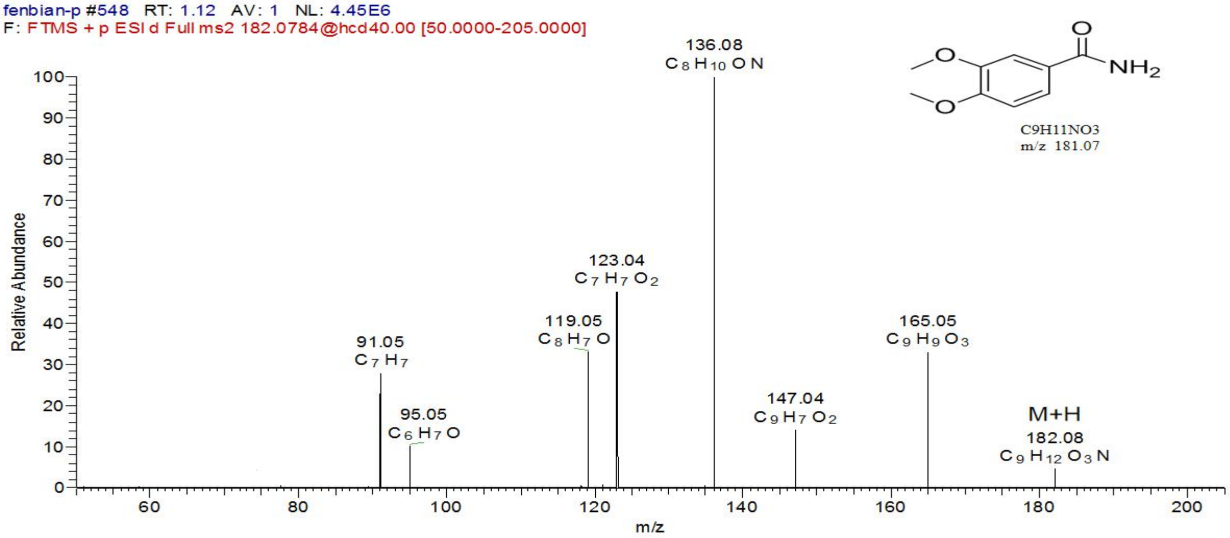
fenbian-p #548 RT: 1.12 AV: 1 NL: 4.45E6
F: FTMS + p ESI d Full ms2 182.0784@hcd40.00 [50.0000-205.0000]
Relative Abundance
m/z
136.08
C8 H10 O N
123.04
C7 H7 O2
119.05
C8 H7 O
91.05
C7 H7
95.05
C6 H7 O
147.04
C9 H7 O2
165.05
C9 H9 O3
M+H
182.08
C9 H12 O3 N
NH2
C9H11NO3
m/z 181.07

M7

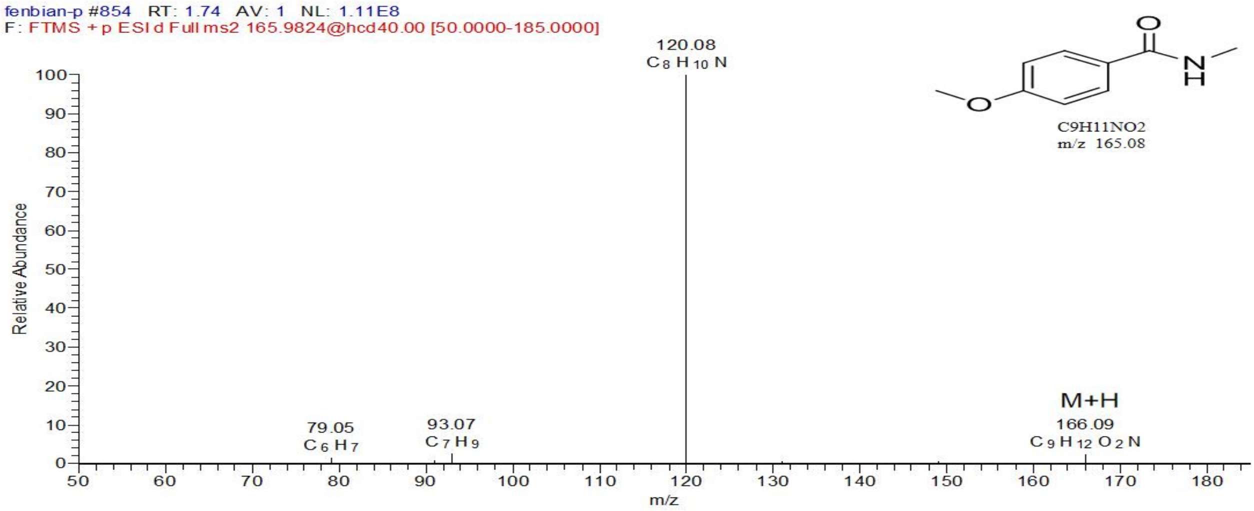
fenbian-p #854 RT: 1.74 AV: 1 NL: 1.11E8
F: FTMS + p ESI d Full ms2 165.9824@hcd40.00 [50.0000-185.0000]
Relative Abundance
m/z
120.08
C8 H10 N
79.05
C6 H7
93.07
C7 H9
M+H
166.09
C9 H12 O2 N
C9H11NO2
m/z 165.08

M8

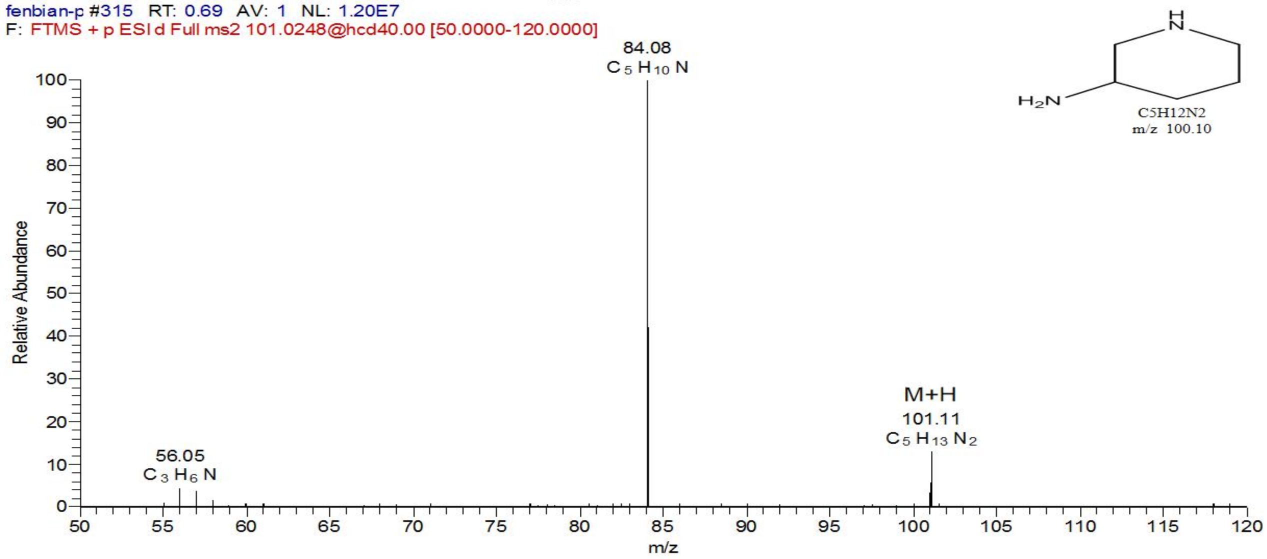
fenbian-p #315 RT: 0.69 AV: 1 NL: 1.20E7
F: FTMS + p ESI d Full ms2 101.0248@hcd40.00 [50.0000-120.0000]
Relative Abundance
m/z
84.08
C5 H10 N
56.05
C3 H6 N
M+H
101.11
C5 H13 N2
H2N
C5H12N2
m/z 100.10

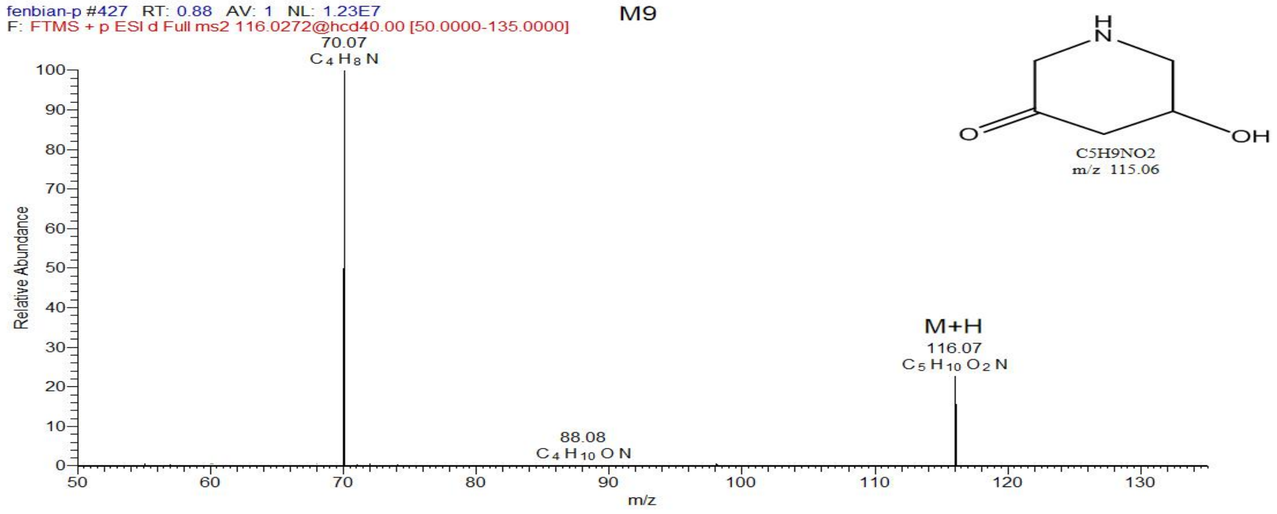
fenbian-p #427 RT: 0.88 AV: 1 NL: 1.23E7
F: FTMS + p ESI d Full ms2 116.0272@hcd40.00 [50.0000-135.0000]
M9
70.07
C4 H8 N
88.08
C4 H10 O N
M+H
116.07
C5 H10 O2 N
C5H9NO2
m/z 115.06
Relative Abundance
m/z

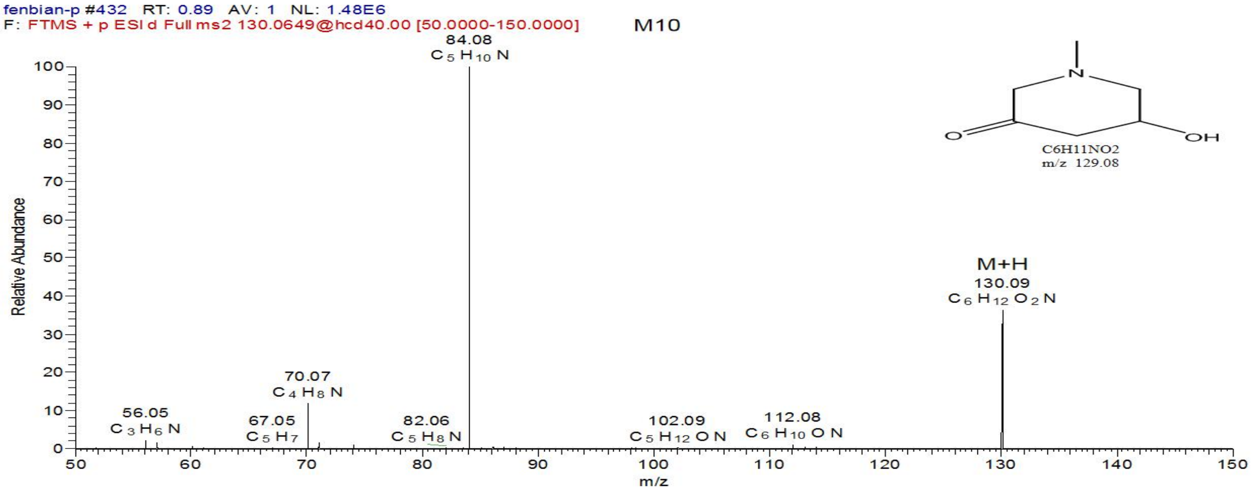
fenbian-p #432 RT: 0.89 AV: 1 NL: 1.48E6
F: FTMS + p ESI d Full ms2 130.0649@hcd40.00 [50.0000-150.0000]
M10
84.08
C5 H10 N
56.05
C3 H6 N
67.05
C5 H7
70.07
C4 H8 N
82.06
C5 H8 N
102.09
C5 H12 O N
112.08
C6 H10 O N
M+H
130.09
C6 H12 O2 N
C6H11NO2
m/z 129.08
Relative Abundance
m/z

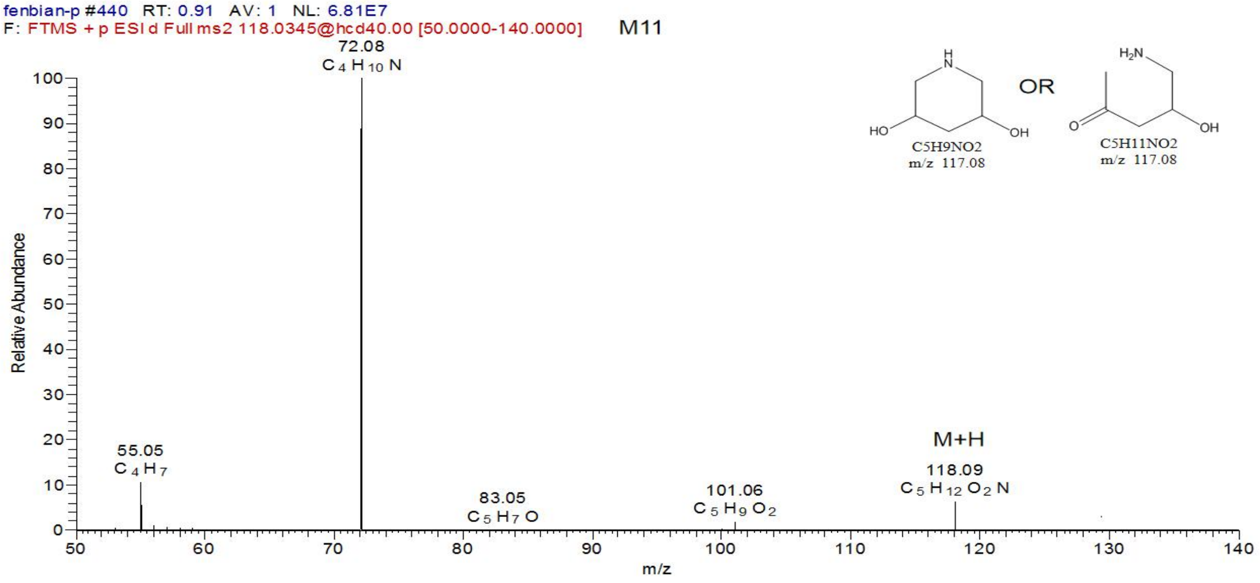
fenbian-p #440 RT: 0.91 AV: 1 NL: 6.81E7
F: FTMS + p ESI d Full ms2 118.0345@hcd40.00 [50.0000-140.0000]
M11
72.08
C4 H10 N
55.05
C4 H7
83.05
C5 H7 O
101.06
C5 H9 O2
M+H
118.09
C5 H12 O2 N
OR
C5H9NO2
m/z 117.08
C5H11NO2
m/z 117.08
Relative Abundance
m/z

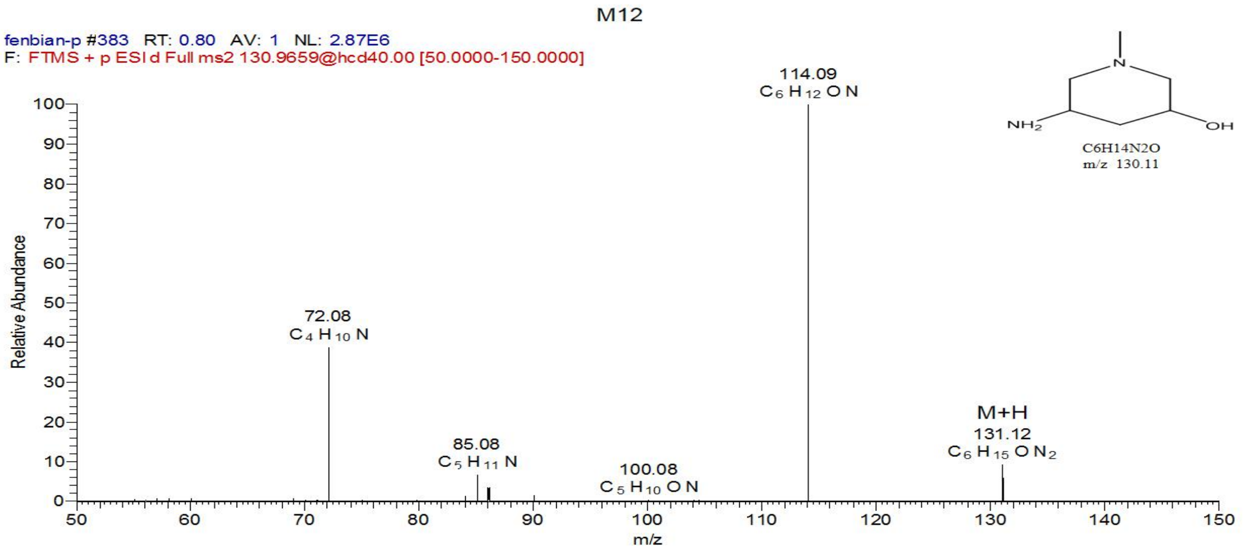
M12
fenbian-p #383 RT: 0.80 AV: 1 NL: 2.87E6
F: FTMS + p ESI d Full ms2 130.9659@hcd40.00 [50.0000-150.0000]
114.09
C6 H12 O N
72.08
C4 H10 N
85.08
C5 H11 N
100.08
C5 H10 O N
M+H
131.12
C6 H15 O N2
Relative Abundance
m/z
NH2
N
OH
C6H14N2O
m/z 130.11

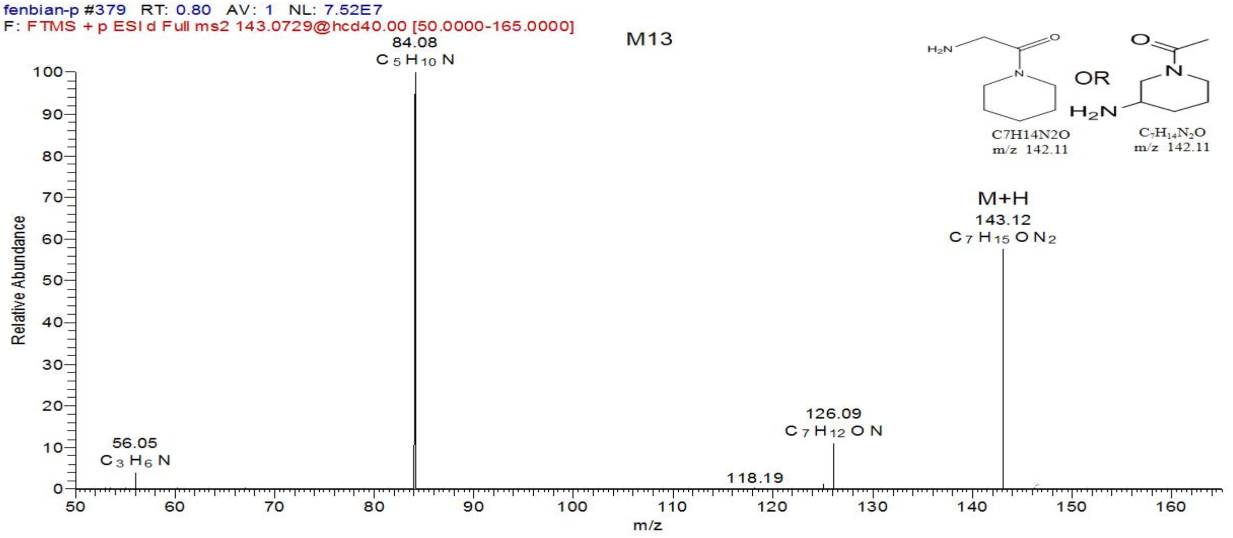
fenbian-p #379 RT: 0.80 AV: 1 NL: 7.52E7
F: FTMS + p ESI d Full ms2 143.0729@hcd40.00 [50.0000-165.0000]
M13
84.08
C5 H10 N
M+H
143.12
C7 H15 O N2
126.09
C7 H12 O N
56.05
C3 H6 N
118.19
Relative Abundance
m/z
H2N
O
N
OR
H2N
C7H14N2O
m/z 142.11
C7H14N2O
m/z 142.11

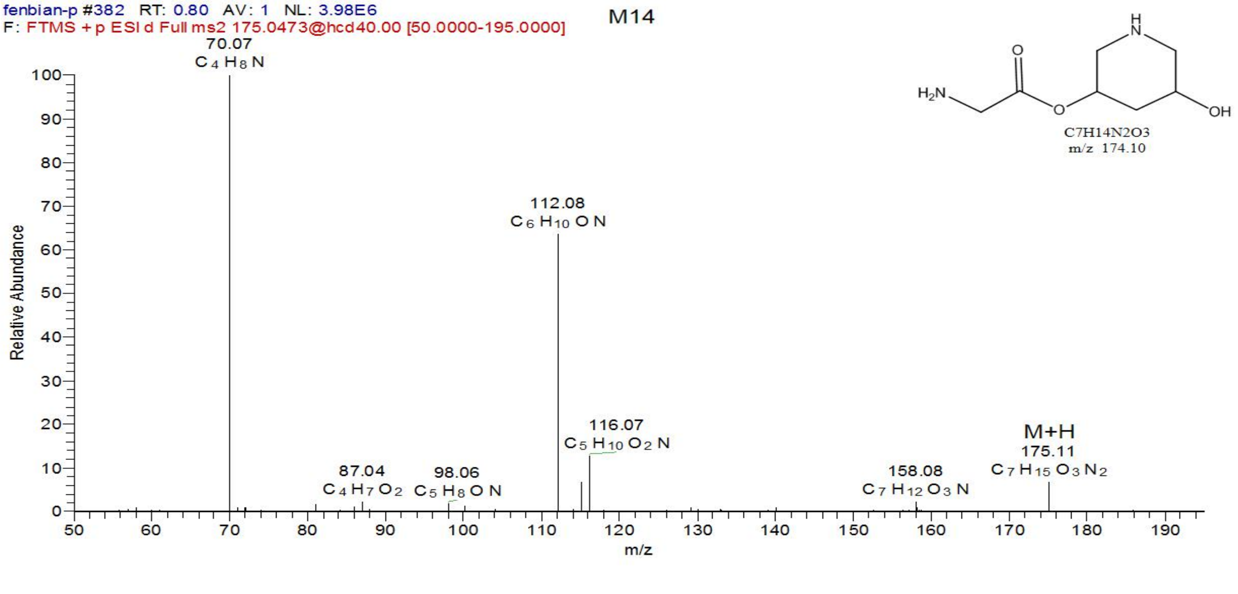
fenbian-p #382 RT: 0.80 AV: 1 NL: 3.98E6
F: FTMS + p ESI d Full ms2 175.0473@hcd40.00 [50.0000-195.0000]
M14
70.07
C4 H8 N
112.08
C6 H10 O N
116.07
C5 H10 O2 N
87.04
C4 H7 O2
98.06
C5 H8 O N
158.08
C7 H12 O3 N
M+H
175.11
C7 H15 O3 N2
Relative Abundance
m/z
H
N
O
H2N
O
OH
C7H14N2O3
m/z 174.10

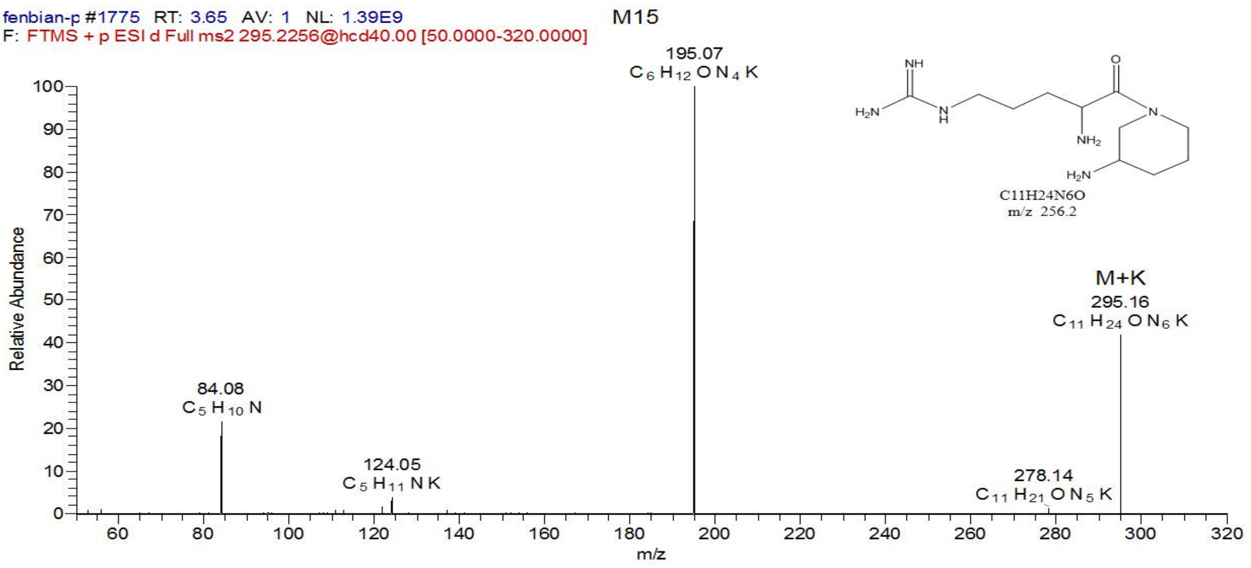
M15
fenbian-p #1775 RT: 3.65 AV: 1 NL: 1.39E9
F: FTMS + p ESI d Full ms2 295.2256@hcd40.00 [50.0000-320.0000]
195.07
C6 H12 O N4 K
C11H24N6O
m/z 256.2
M+K
295.16
C11 H24 O N6 K
84.08
C5 H10 N
124.05
C5 H11 N K
278.14
C11 H21 O N5 K
Relative Abundance
m/z

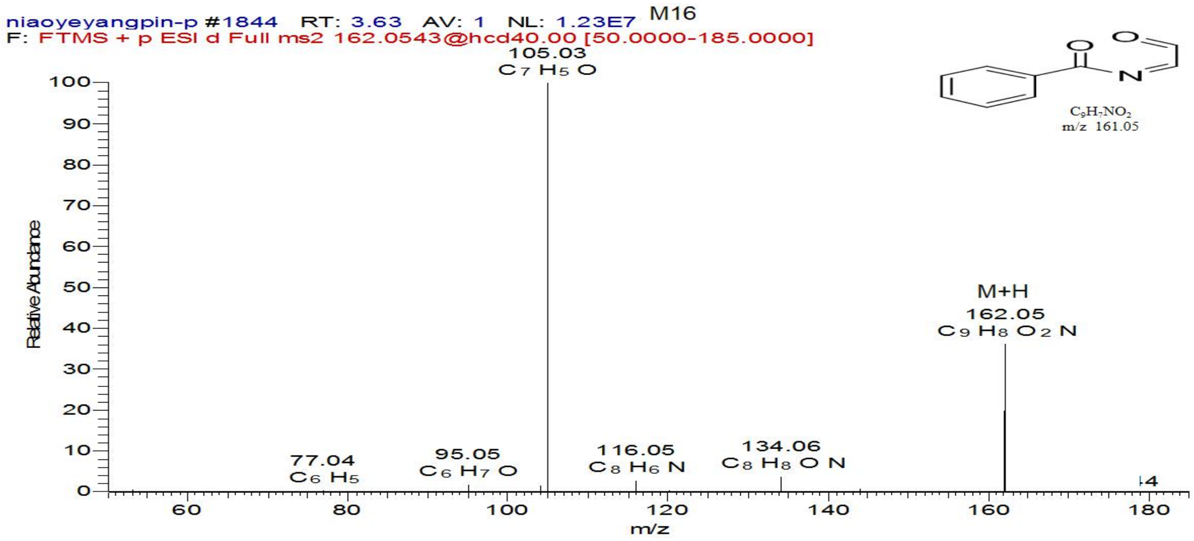
M16
niaoyeyangpin-p #1844 RT: 3.63 AV: 1 NL: 1.23E7
F: FTMS + p ESI d Full ms2 162.0543@hcd40.00 [50.0000-185.0000]
105.03
C7 H5 O
C9H7NO2
m/z 161.05
M+H
162.05
C9 H8 O2 N
77.04
C6 H5
95.05
C6 H7 O
116.05
C8 H6 N
134.06
C8 H8 O N
Relative Abundance
m/z

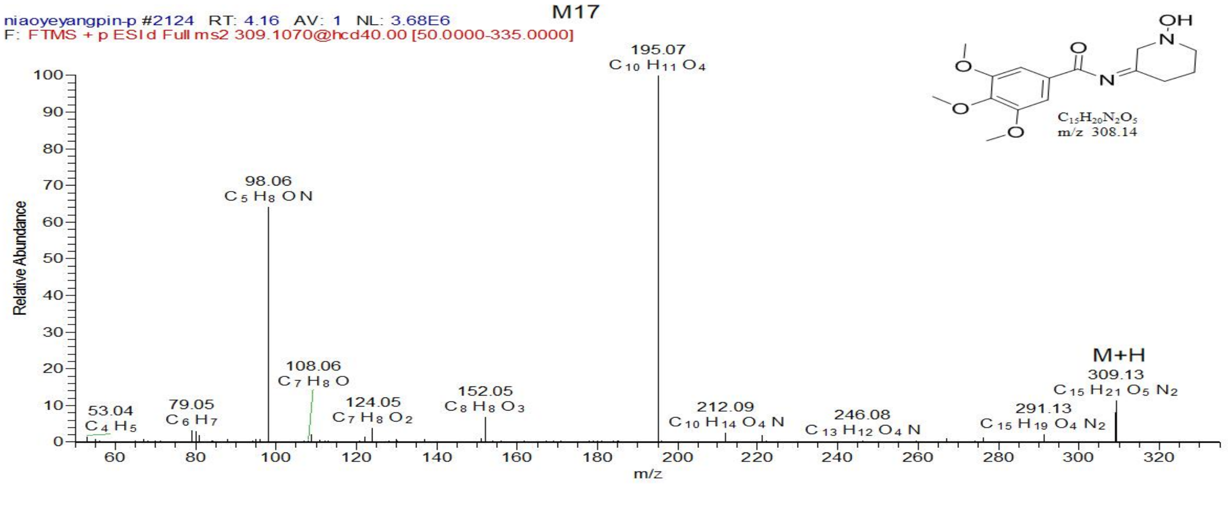
M17
niaoyeyangpin-p #2124 RT: 4.16 AV: 1 NL: 3.68E6
F: FTMS + p ESI d Full ms2 309.1070@hcd40.00 [50.0000-335.0000]
195.07
C10 H11 O4
C15H20N2O5
m/z 308.14
98.06
C5 H8 O N
108.06
C7 H8 O
124.05
C7 H8 O2
152.05
C8 H8 O3
53.04
C4 H5
79.05
C6 H7
212.09
C10 H14 O4 N
246.08
C13 H12 O4 N
291.13
C15 H19 O4 N2
M+H
309.13
C15 H21 O5 N2
Relative Abundance
m/z

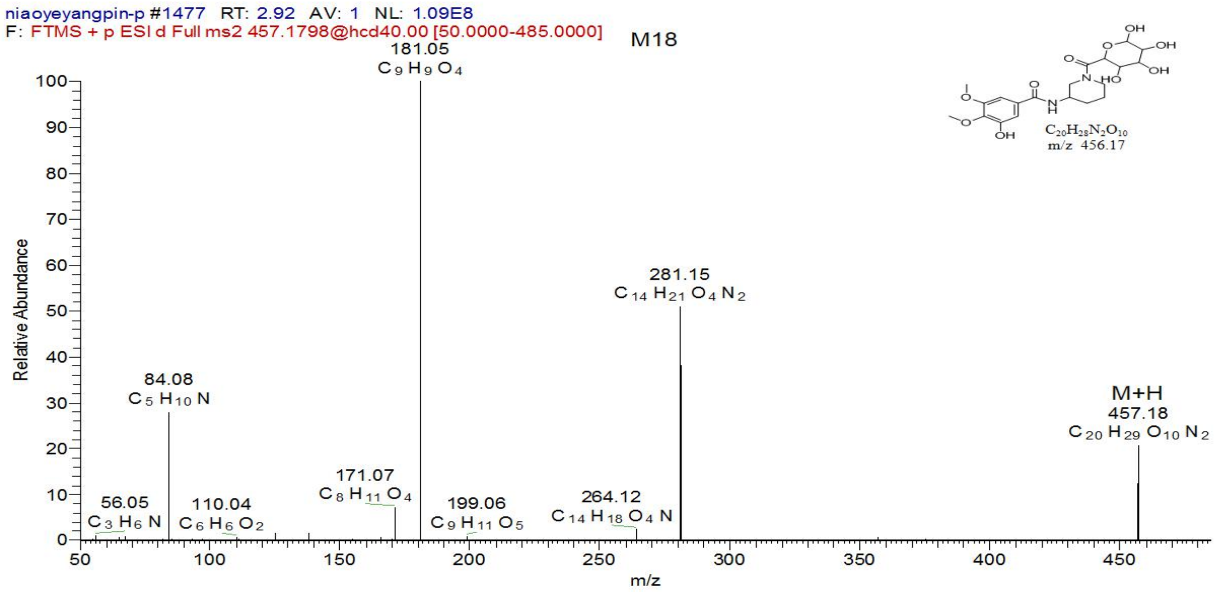
niaoyeyangpin-p #1477 RT: 2.92 AV: 1 NL: 1.09E8
F: FTMS + p ESI d Full ms2 457.1798@hcd40.00 [50.0000-485.0000]
M18
181.05
$C_9H_9O_4$
281.15
$C_{14}H_{21}O_4N_2$
84.08
$C_5H_{10}N$
M+H
457.18
$C_{20}H_{29}O_{10}N_2$
171.07
$C_8H_{11}O_4$
56.05
$C_3H_6N$
110.04
$C_6H_6O_2$
199.06
$C_9H_{11}O_5$
264.12
$C_{14}H_{18}O_4N$
$C_{20}H_{28}N_2O_{10}$
m/z 456.17
Relative Abundance
m/z

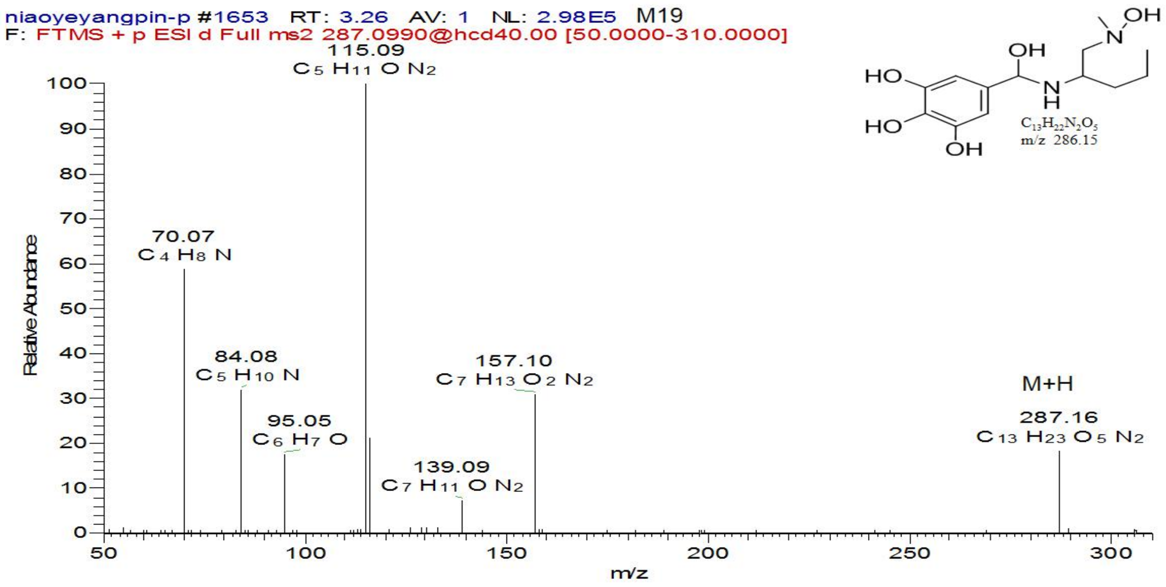
niaoyeyangpin-p #1653 RT: 3.26 AV: 1 NL: 2.98E5 M19
F: FTMS + p ESI d Full ms2 287.0990@hcd40.00 [50.0000-310.0000]
115.09
$C_5H_{11}ON_2$
70.07
$C_4H_8N$
84.08
$C_5H_{10}N$
95.05
$C_6H_7O$
157.10
$C_7H_{13}O_2N_2$
139.09
$C_7H_{11}ON_2$
M+H
287.16
$C_{13}H_{23}O_5N_2$
$C_{13}H_{22}N_2O_5$
m/z 286.15
Relative Abundance
m/z

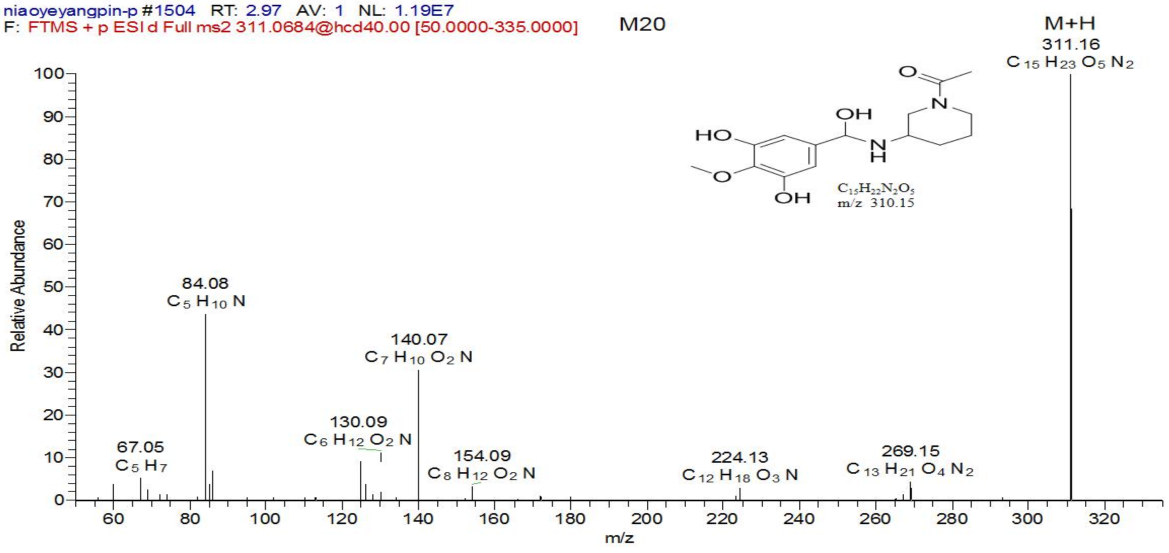
niaoyeyangpin-p #1504 RT: 2.97 AV: 1 NL: 1.19E7
F: FTMS + p ESI d Full ms2 311.0684@hcd40.00 [50.0000-335.0000]
M20
M+H
311.16
$C_{15}H_{23}O_5N_2$
$C_{15}H_{22}N_2O_5$
m/z 310.15
84.08
$C_5H_{10}N$
140.07
$C_7H_{10}O_2N$
130.09
$C_6H_{12}O_2N$
67.05
$C_5H_7$
154.09
$C_8H_{12}O_2N$
224.13
$C_{12}H_{18}O_3N$
269.15
$C_{13}H_{21}O_4N_2$
Relative Abundance
m/z

M21

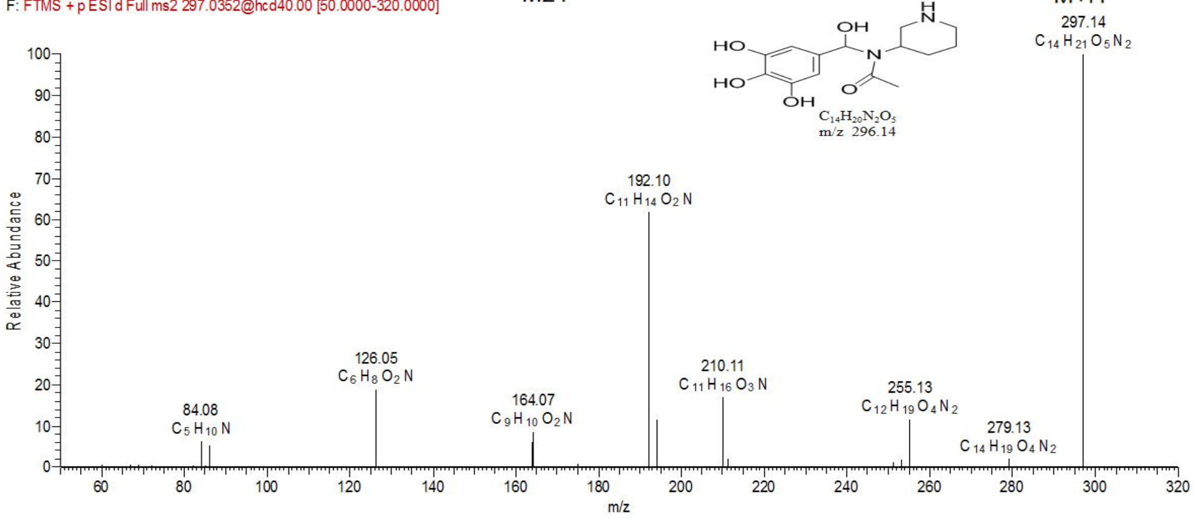
niaoyeyangpin-p #1079 RT: 2.11 AV: 1 NL: 1.12E8
F: FTMS + p ESI d Full ms2 297.0352@hcd40.00 [50.0000-320.0000]
M+H
297.14
C 14 H 21 O 5 N 2
C14H20N2O5
m/z 296.14
192.10
C 11 H 14 O 2 N
126.05
C 6 H 8 O 2 N
210.11
C 11 H 16 O 3 N
164.07
C 9 H 10 O 2 N
255.13
C 12 H 19 O 4 N 2
84.08
C 5 H 10 N
279.13
C 14 H 19 O 4 N 2
Relative Abundance
m/z

M22

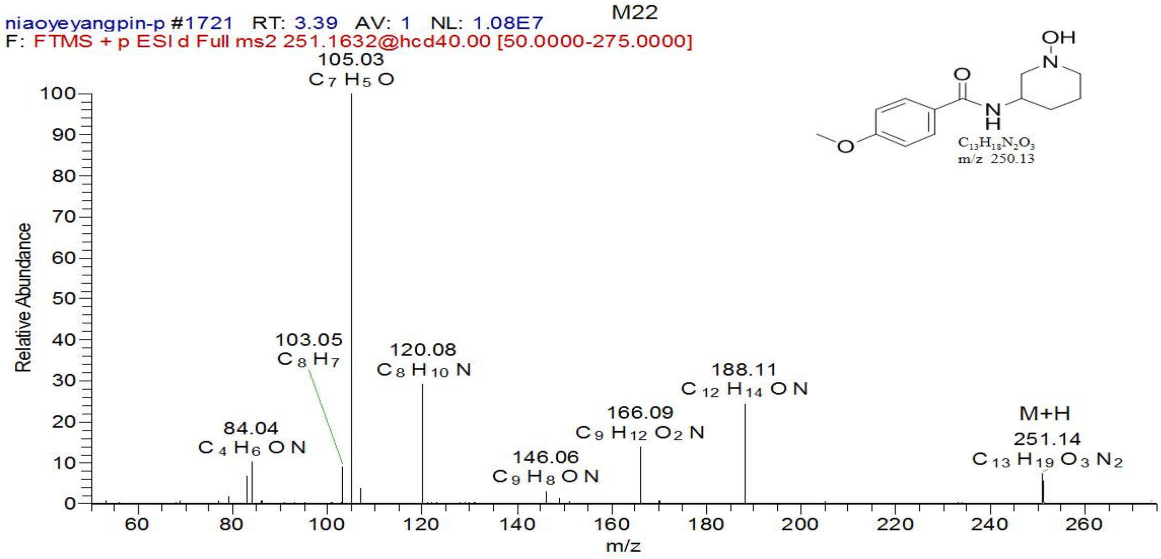
niaoyeyangpin-p #1721 RT: 3.39 AV: 1 NL: 1.08E7
F: FTMS + p ESI d Full ms2 251.1632@hcd40.00 [50.0000-275.0000]
105.03
C 7 H 5 O
C13H18N2O3
m/z 250.13
103.05
C 8 H 7
120.08
C 8 H 10 N
188.11
C 12 H 14 O N
166.09
C 9 H 12 O 2 N
84.04
C 4 H 6 O N
146.06
C 9 H 8 O N
M+H
251.14
C 13 H 19 O 3 N 2
Relative Abundance
m/z

M23

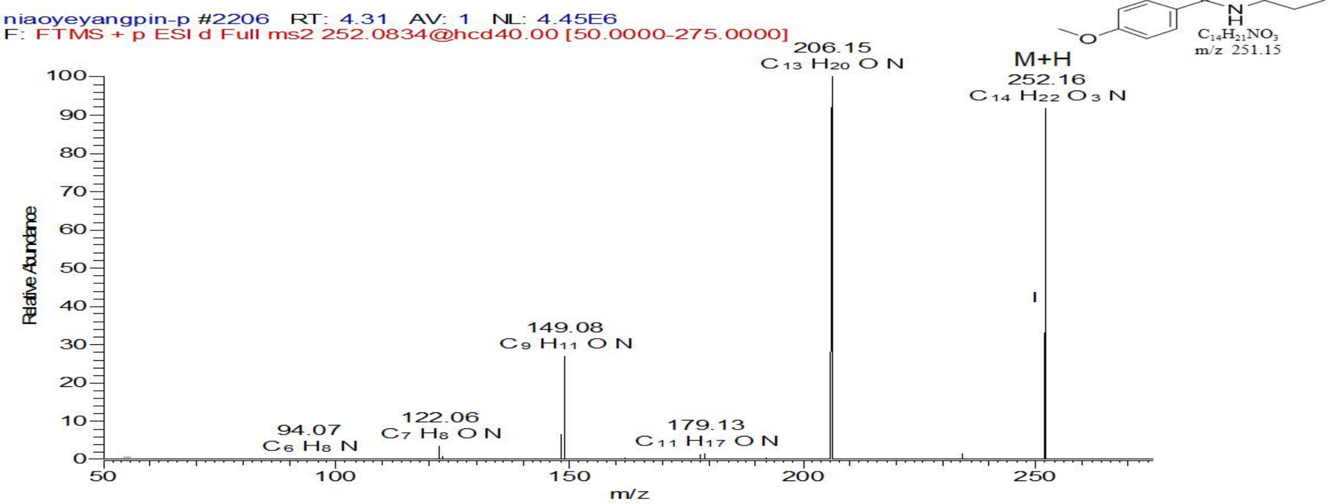
niaoyeyangpin-p #2206 RT: 4.31 AV: 1 NL: 4.45E6
F: FTMS + p ESI d Full ms2 252.0834@hcd40.00 [50.0000-275.0000]
C14H21NO3
m/z 251.15
206.15
C 13 H 20 O N
M+H
252.16
C 14 H 22 O 3 N
149.08
C 9 H 11 O N
122.06
C 7 H 8 O N
94.07
C 6 H 8 N
179.13
C 11 H 17 O N
Relative Abundance
m/z

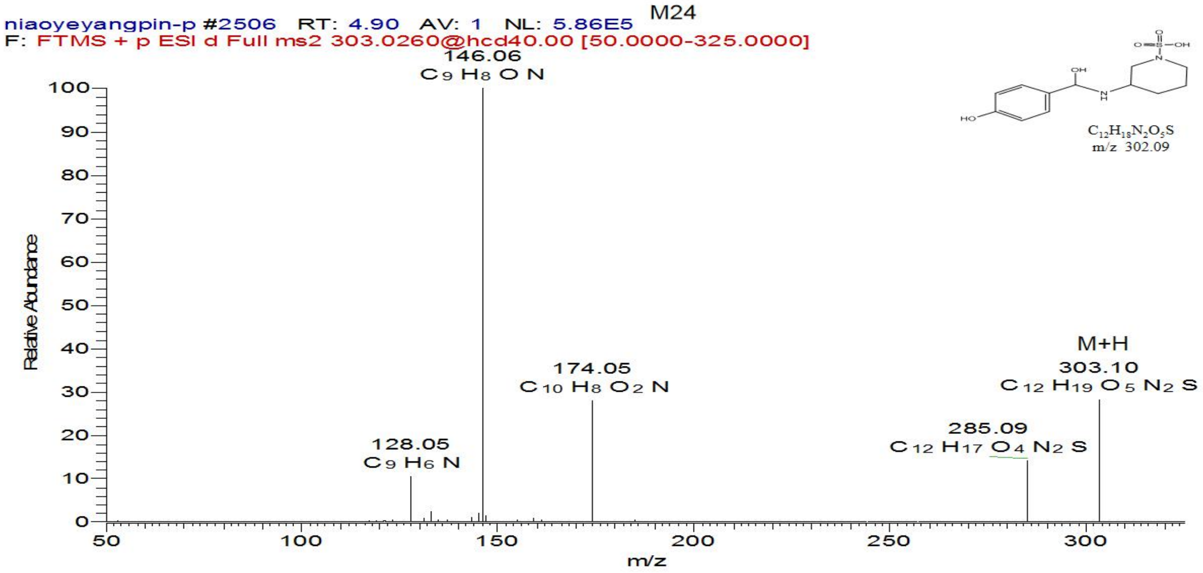
M24
niaoyeyangpin-p #2506 RT: 4.90 AV: 1 NL: 5.86E5
F: FTMS + p ESI d Full ms2 303.0260@hcd40.00 [50.0000-325.0000]
146.06
C9 H8 O N
174.05
C10 H8 O2 N
128.05
C9 H6 N
285.09
C12 H17 O4 N2 S
M+H
303.10
C12 H19 O5 N2 S
$C_{12}H_{18}N_2O_5S$
m/z 302.09
Relative Abundance
m/z

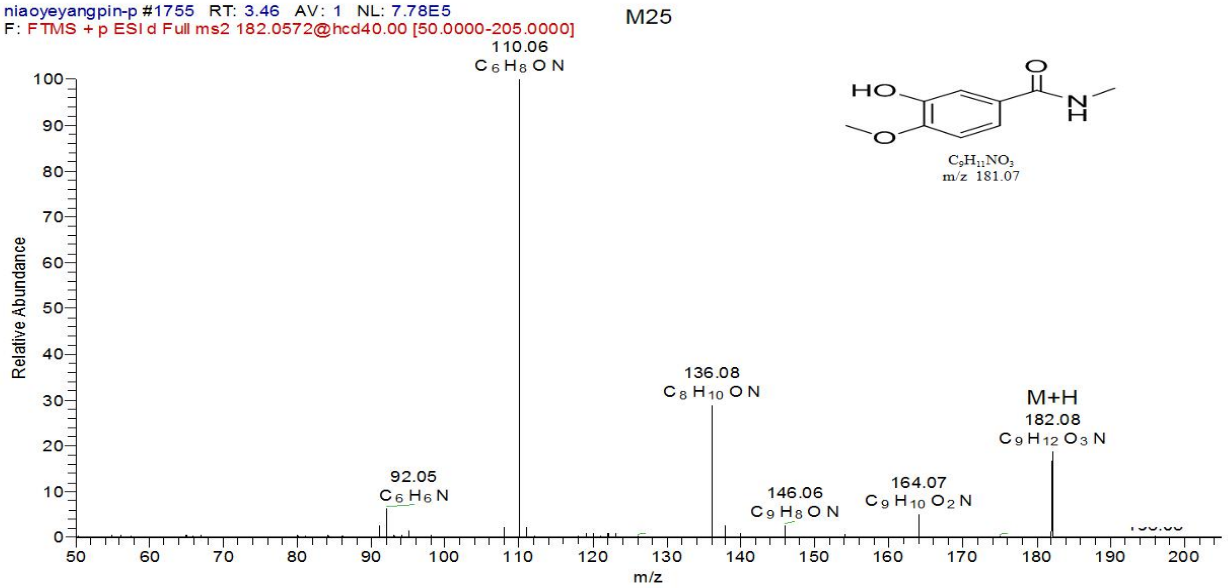
M25
niaoyeyangpin-p #1755 RT: 3.46 AV: 1 NL: 7.78E5
F: FTMS + p ESI d Full ms2 182.0572@hcd40.00 [50.0000-205.0000]
110.06
C6 H8 O N
136.08
C8 H10 O N
92.05
C6 H6 N
146.06
C9 H8 O N
164.07
C9 H10 O2 N
M+H
182.08
C9 H12 O3 N
HO
O
N
H
$C_9H_{11}NO_3$
m/z 181.07
Relative Abundance
m/z

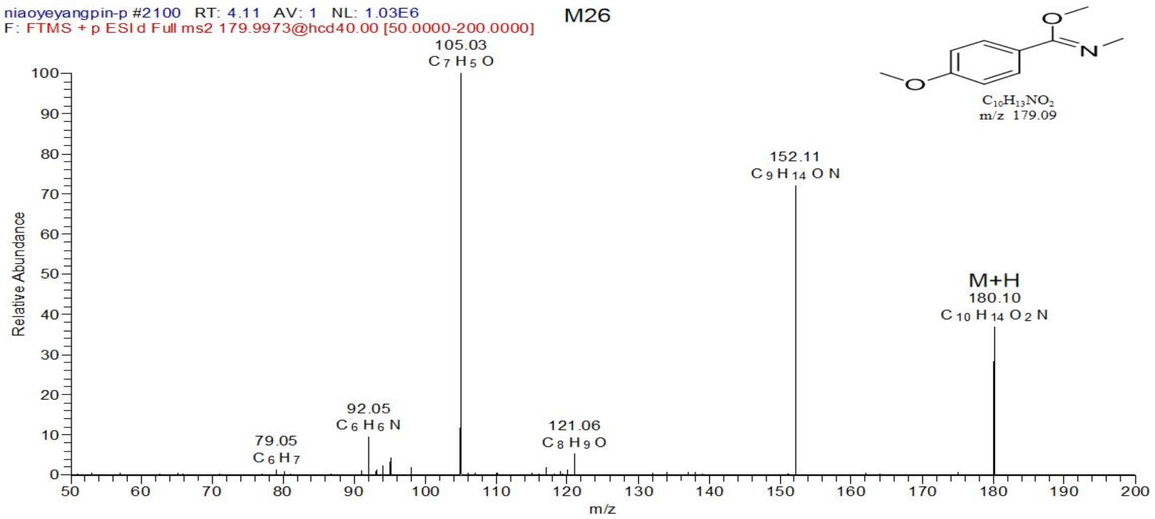
M26
niaoyeyangpin-p #2100 RT: 4.11 AV: 1 NL: 1.03E6
F: FTMS + p ESI d Full ms2 179.9973@hcd40.00 [50.0000-200.0000]
105.03
C7 H5 O
152.11
C9 H14 O N
M+H
180.10
C10 H14 O2 N
92.05
C6 H6 N
79.05
C6 H7
121.06
C8 H9 O
$C_{10}H_{13}NO_2$
m/z 179.09
Relative Abundance
m/z

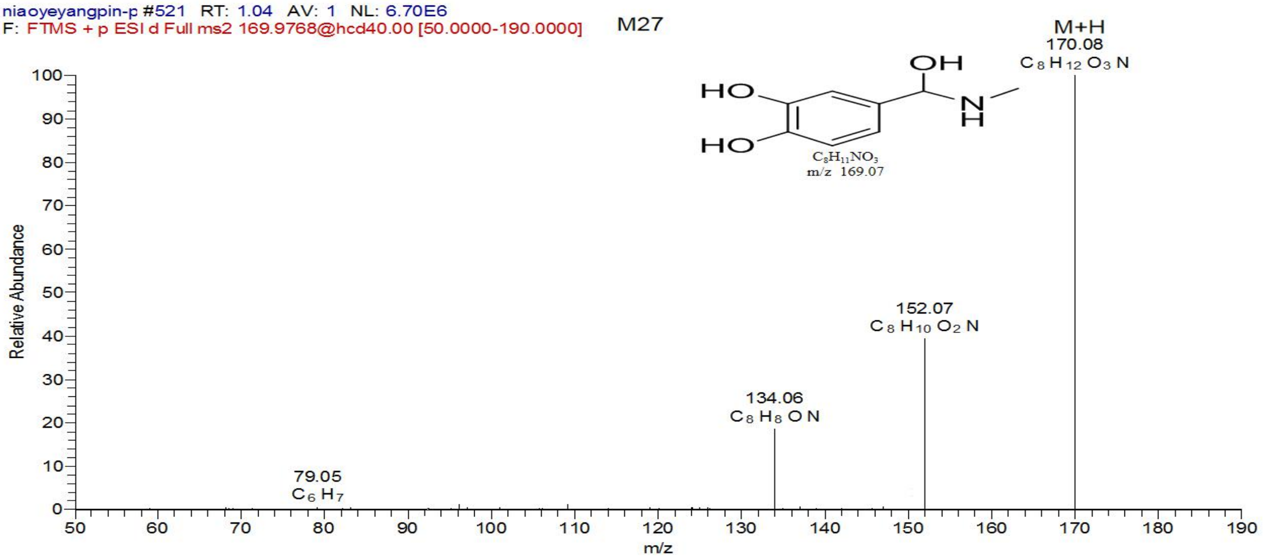
niaoyeyangpin-p #521 RT: 1.04 AV: 1 NL: 6.70E6
F: FTMS + p ESI d Full ms2 169.9768@hcd40.00 [50.0000-190.0000]
M27
M+H
170.08
C8 H12 O3 N
HO
HO
OH
N
H
C8H11NO3
m/z 169.07
152.07
C8 H10 O2 N
134.06
C8 H8 O N
79.05
C6 H7
Relative Abundance
m/z

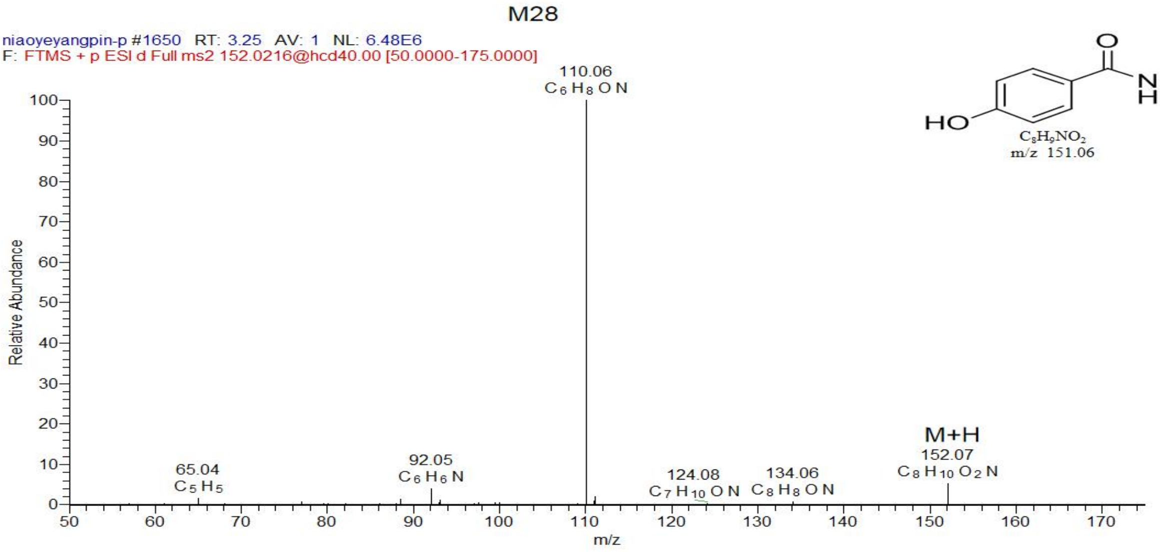
M28
niaoyeyangpin-p #1650 RT: 3.25 AV: 1 NL: 6.48E6
F: FTMS + p ESI d Full ms2 152.0216@hcd40.00 [50.0000-175.0000]
110.06
C6 H8 O N
O
N
H
HO
C8H9NO2
m/z 151.06
M+H
152.07
C8 H10 O2 N
65.04
C5 H5
92.05
C6 H6 N
124.08
C7 H10 O N
134.06
C8 H8 O N
Relative Abundance
m/z

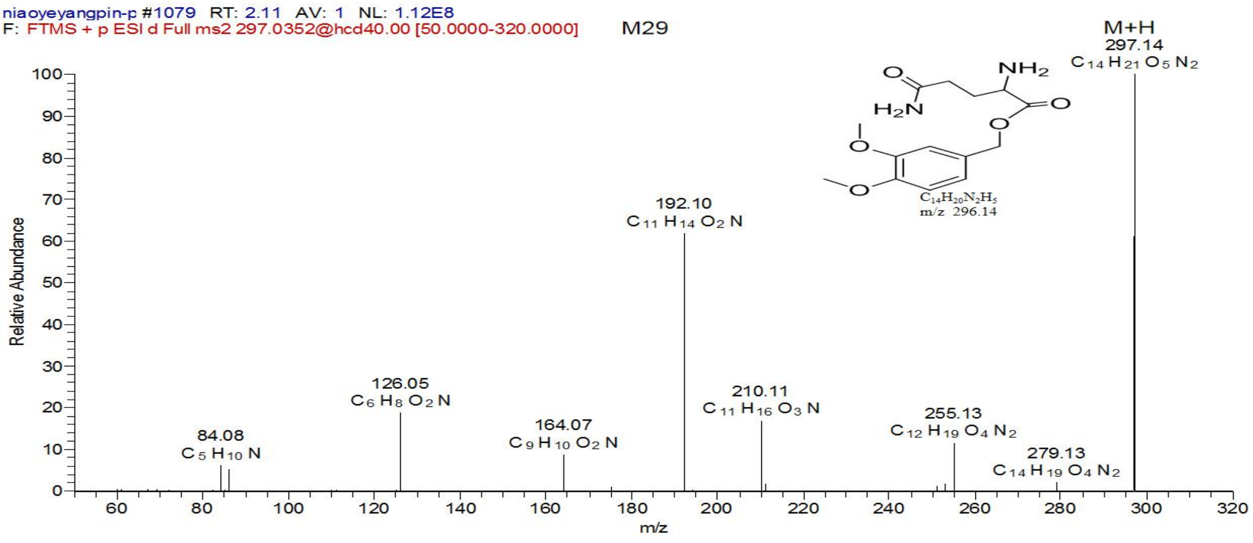
niaoyeyangpin-p #1079 RT: 2.11 AV: 1 NL: 1.12E8
F: FTMS + p ESI d Full ms2 297.0352@hcd40.00 [50.0000-320.0000]
M29
M+H
297.14
C14 H21 O5 N2
NH2
H2N
O
C14H20N2H5
m/z 296.14
192.10
C11 H14 O2 N
126.05
C6 H8 O2 N
164.07
C9 H10 O2 N
210.11
C11 H16 O3 N
255.13
C12 H19 O4 N2
279.13
C14 H19 O4 N2
84.08
C5 H10 N
Relative Abundance
m/z

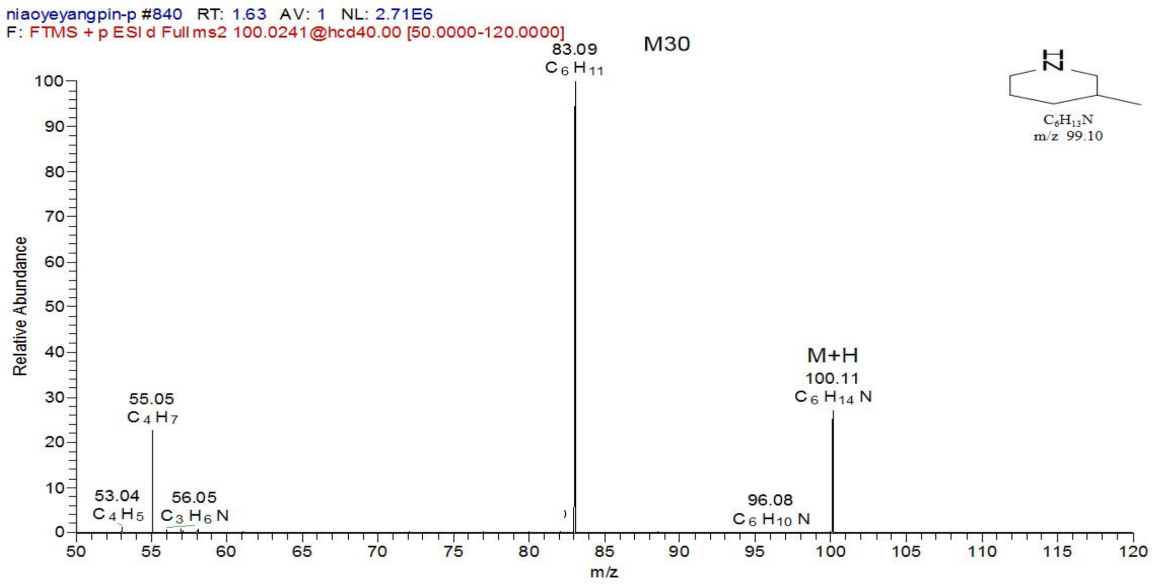
niaoyeyangpin-p #840 RT: 1.63 AV: 1 NL: 2.71E6
F: FTMS + p ESI d Full ms2 100.0241@hcd40.00 [50.0000-120.0000]
M30
83.09
C6 H11
C6H13N
m/z 99.10
M+H
100.11
C6 H14 N
55.05
C4 H7
53.04
C4 H5
56.05
C3 H6 N
96.08
C6 H10 N
Relative Abundance
m/z

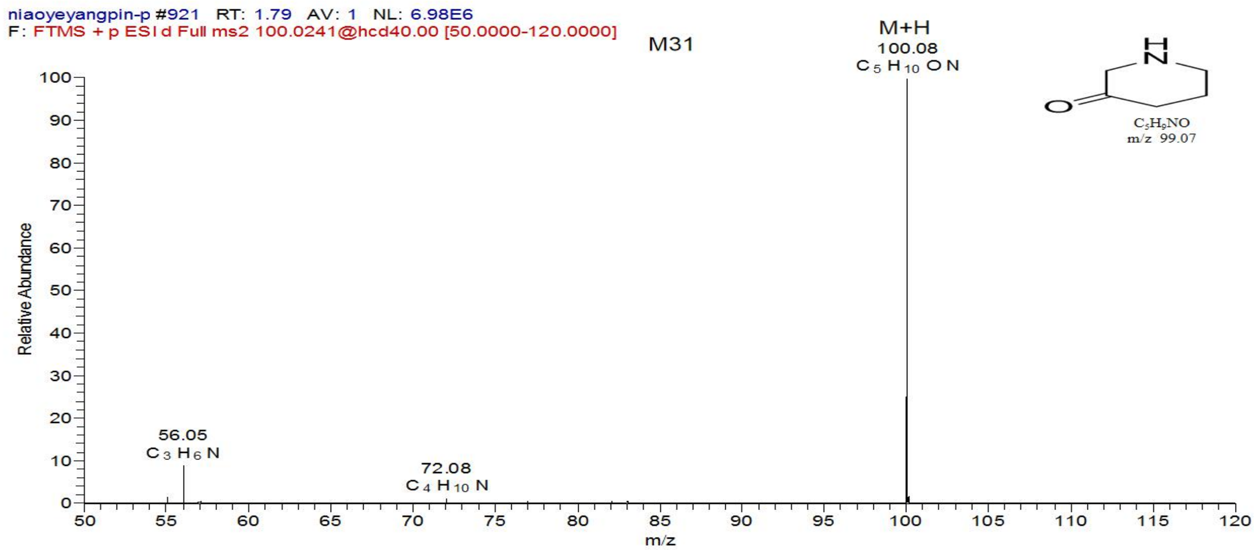
niaoyeyangpin-p #921 RT: 1.79 AV: 1 NL: 6.98E6
F: FTMS + p ESI d Full ms2 100.0241@hcd40.00 [50.0000-120.0000]
M31
M+H
100.08
C5 H10 O N
C5H9NO
m/z 99.07
56.05
C3 H6 N
72.08
C4 H10 N
Relative Abundance
m/z

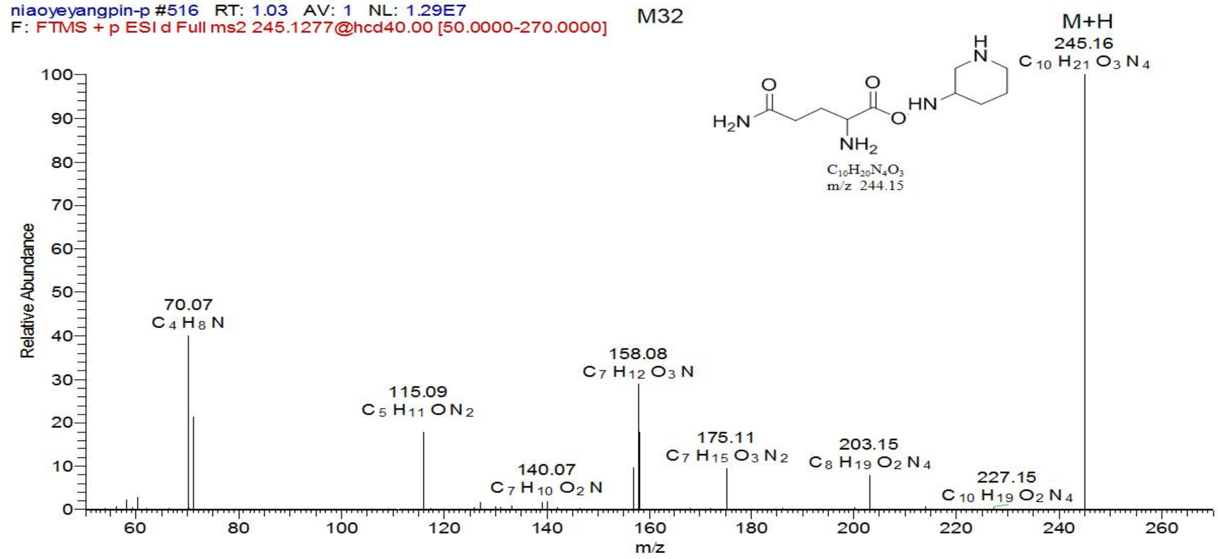
niaoyeyangpin-p #516 RT: 1.03 AV: 1 NL: 1.29E7
F: FTMS + p ESI d Full ms2 245.1277@hcd40.00 [50.0000-270.0000]
M32
M+H
245.16
C10 H21 O3 N4
C10H20N4O3
m/z 244.15
70.07
C4 H8 N
115.09
C5 H11 O N2
158.08
C7 H12 O3 N
140.07
C7 H10 O2 N
175.11
C7 H15 O3 N2
203.15
C8 H19 O2 N4
227.15
C10 H19 O2 N4
Relative Abundance
m/z

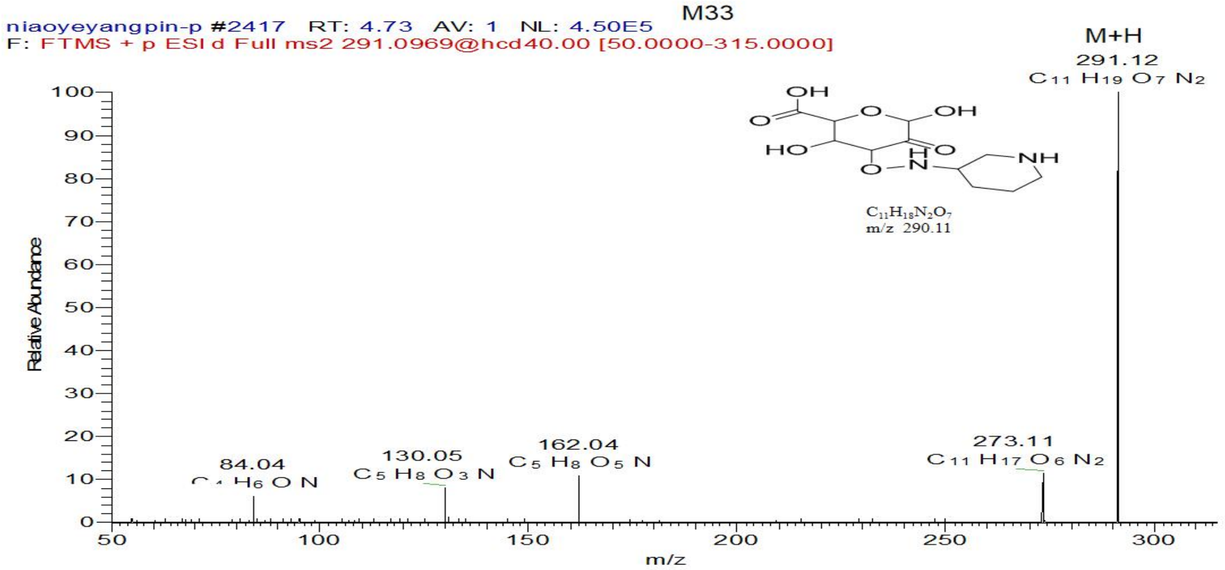
M33
niaoyeyangpin-p #2417 RT: 4.73 AV: 1 NL: 4.50E5
F: FTMS + p ESI d Full ms2 291.0969@hcd40.00 [50.0000-315.0000]
M+H
291.12
C 11 H 19 O 7 N 2
$C_{11}H_{18}N_2O_7$
m/z 290.11
273.11
C 11 H 17 O 6 N 2
162.04
C 5 H 8 O 5 N
130.05
C 5 H 8 O 3 N
84.04
H 6 O N
Relative Abundance
m/z

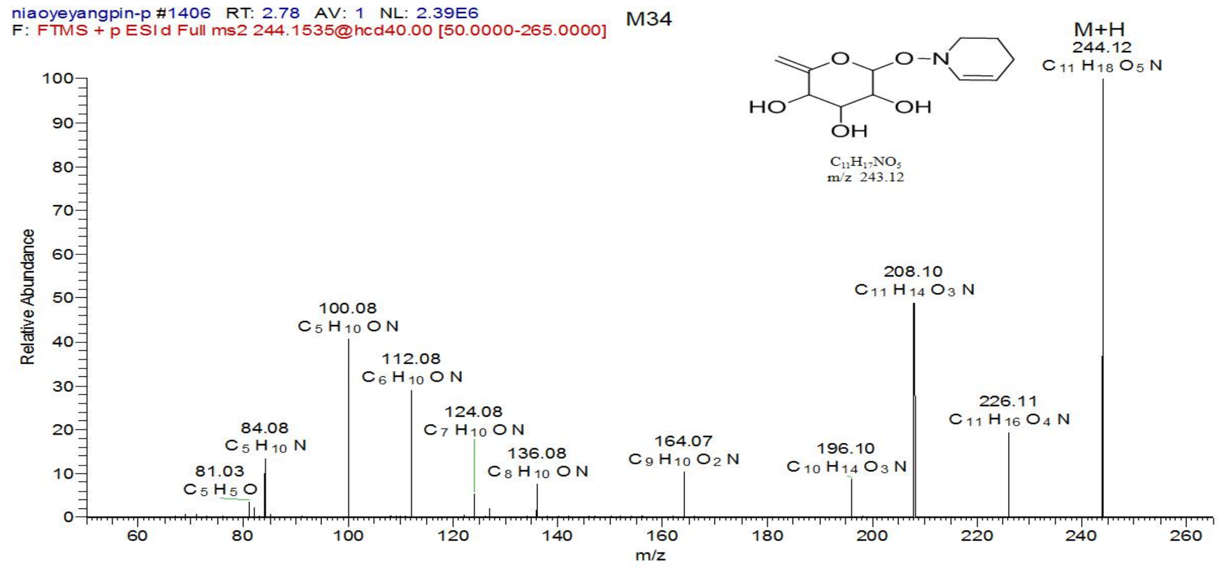
M34
niaoyeyangpin-p #1406 RT: 2.78 AV: 1 NL: 2.39E6
F: FTMS + p ESI d Full ms2 244.1535@hcd40.00 [50.0000-265.0000]
M+H
244.12
C 11 H 18 O 5 N
$C_{11}H_{17}NO_5$
m/z 243.12
208.10
C 11 H 14 O 3 N
226.11
C 11 H 16 O 4 N
196.10
C 10 H 14 O 3 N
164.07
C 9 H 10 O 2 N
136.08
C 8 H 10 O N
124.08
C 7 H 10 O N
112.08
C 6 H 10 O N
100.08
C 5 H 10 O N
84.08
C 5 H 10 N
81.03
C 5 H 5 O
Relative Abundance
m/z

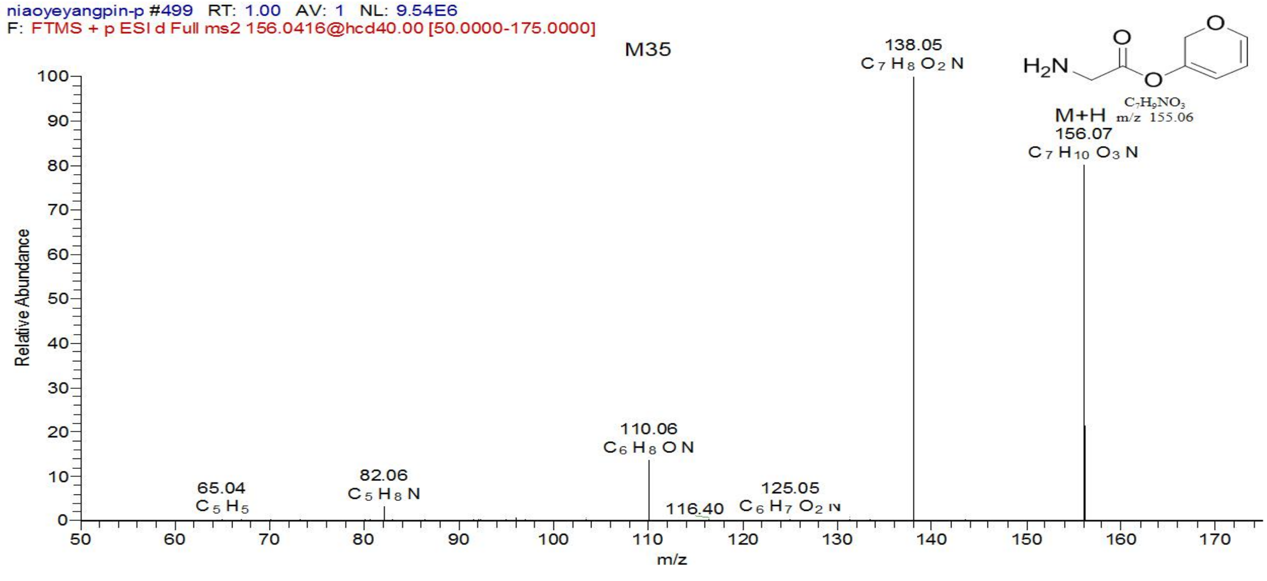
niaoyeyangpin-p #499 RT: 1.00 AV: 1 NL: 9.54E6
F: FTMS + p ESI d Full ms2 156.0416@hcd40.00 [50.0000-175.0000]
M35
138.05
C 7 H 8 O 2 N
$H_2N$
$C_7H_9NO_3$
m/z 155.06
M+H
156.07
C 7 H 10 O 3 N
110.06
C 6 H 8 O N
125.05
C 6 H 7 O 2 N
116.40
82.06
C 5 H 8 N
65.04
C 5 H 5
Relative Abundance
m/z

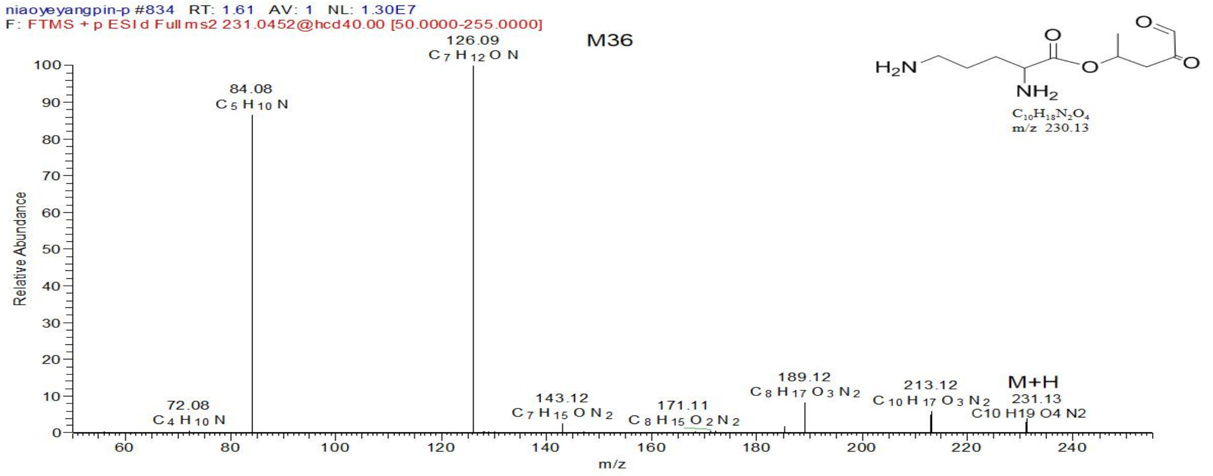
niaoyeyangpin-p #834 RT: 1.61 AV: 1 NL: 1.30E7
F: FTMS + p ESI d Full ms2 231.0452@hcd40.00 [50.0000-255.0000]
M36
126.09
C 7 H 12 O N
84.08
C 5 H 10 N
72.08
C 4 H 10 N
143.12
C 7 H 15 O N 2
171.11
C 8 H 15 O 2 N 2
189.12
C 8 H 17 O 3 N 2
213.12
C 10 H 17 O 3 N 2
M+H
231.13
C10 H19 O4 N2
Relative Abundance
m/z
$C_{10}H_{18}N_2O_4$
m/z 230.13

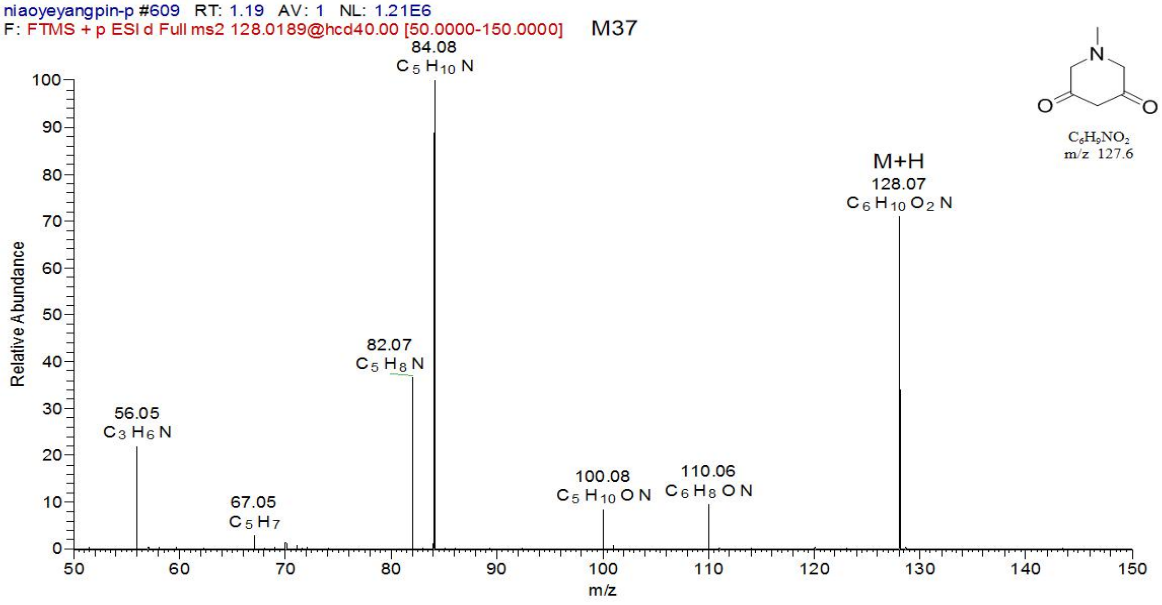
niaoyeyangpin-p #609 RT: 1.19 AV: 1 NL: 1.21E6
F: FTMS + p ESI d Full ms2 128.0189@hcd40.00 [50.0000-150.0000]
M37
84.08
C 5 H 10 N
M+H
128.07
C 6 H 10 O 2 N
82.07
C 5 H 8 N
56.05
C 3 H 6 N
67.05
C 5 H 7
100.08
C 5 H 10 O N
110.06
C 6 H 8 O N
Relative Abundance
m/z
$C_6H_9NO_2$
m/z 127.6

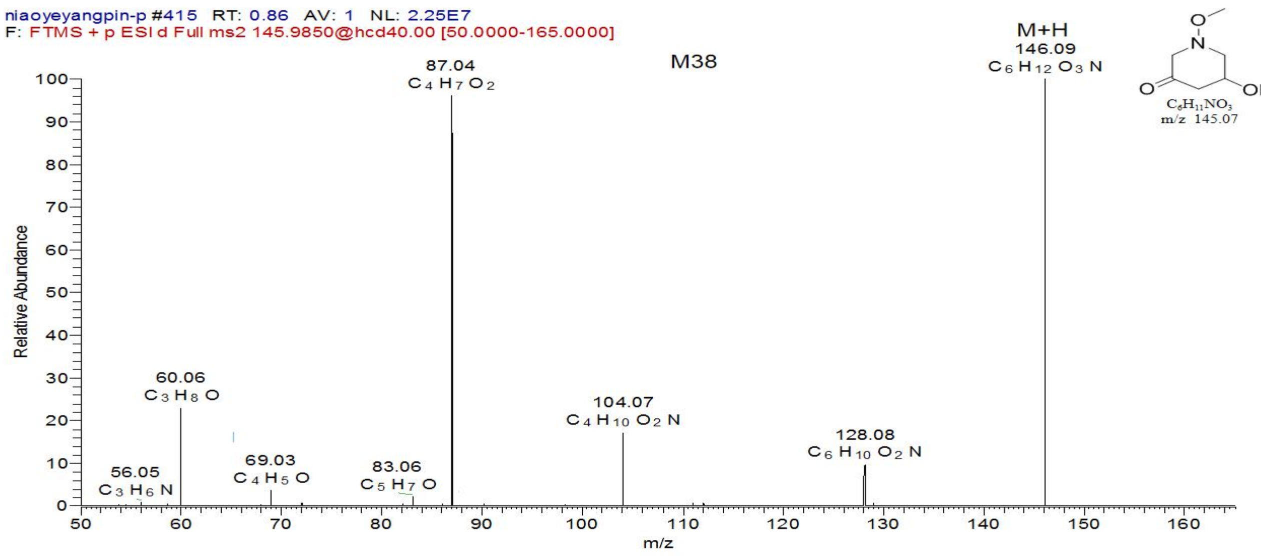
niaoyeyangpin-p #415 RT: 0.86 AV: 1 NL: 2.25E7
F: FTMS + p ESI d Full ms2 145.9850@hcd40.00 [50.0000-165.0000]
M38
M+H
146.09
C 6 H 12 O 3 N
87.04
C 4 H 7 O 2
60.06
C 3 H 8 O
104.07
C 4 H 10 O 2 N
128.08
C 6 H 10 O 2 N
56.05
C 3 H 6 N
69.03
C 4 H 5 O
83.06
C 5 H 7 O
Relative Abundance
m/z
$C_6H_{11}NO_3$
m/z 145.07

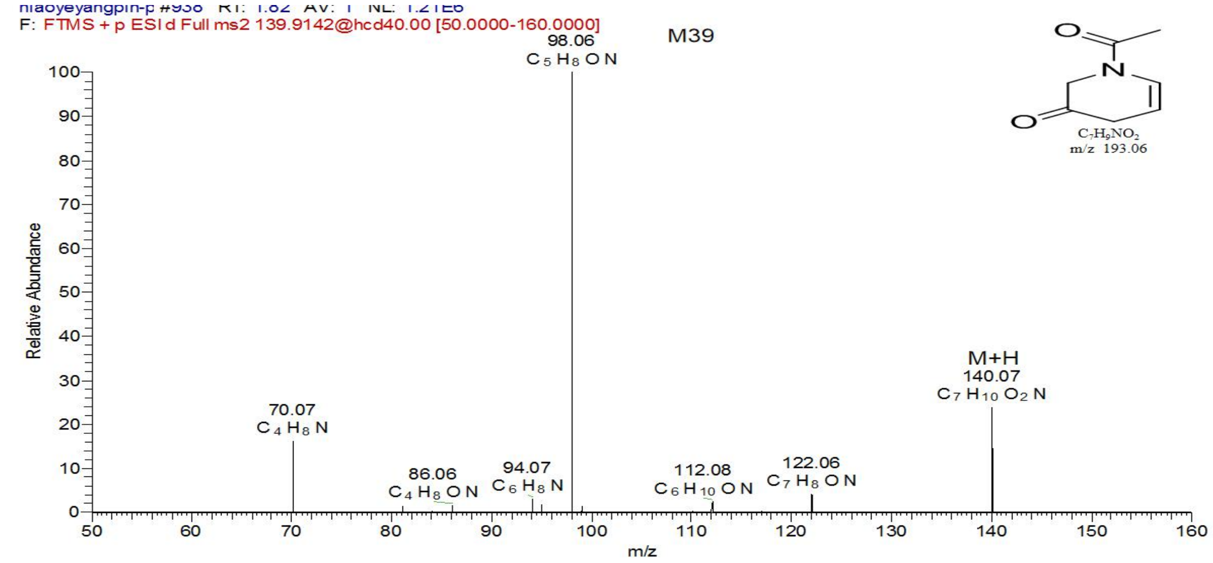
niaoyeyangpin-p #938 RT: 1.62 AV: 1 NL: 1.21E6
F: FTMS + p ESI d Full ms2 139.9142@hcd40.00 [50.0000-160.0000]
M39
98.06
C5 H8 O N
70.07
C4 H8 N
86.06
C4 H8 O N
94.07
C6 H8 N
112.08
C6 H10 O N
122.06
C7 H8 O N
M+H
140.07
C7 H10 O2 N
Relative Abundance
m/z
C7H9NO2
m/z 193.06

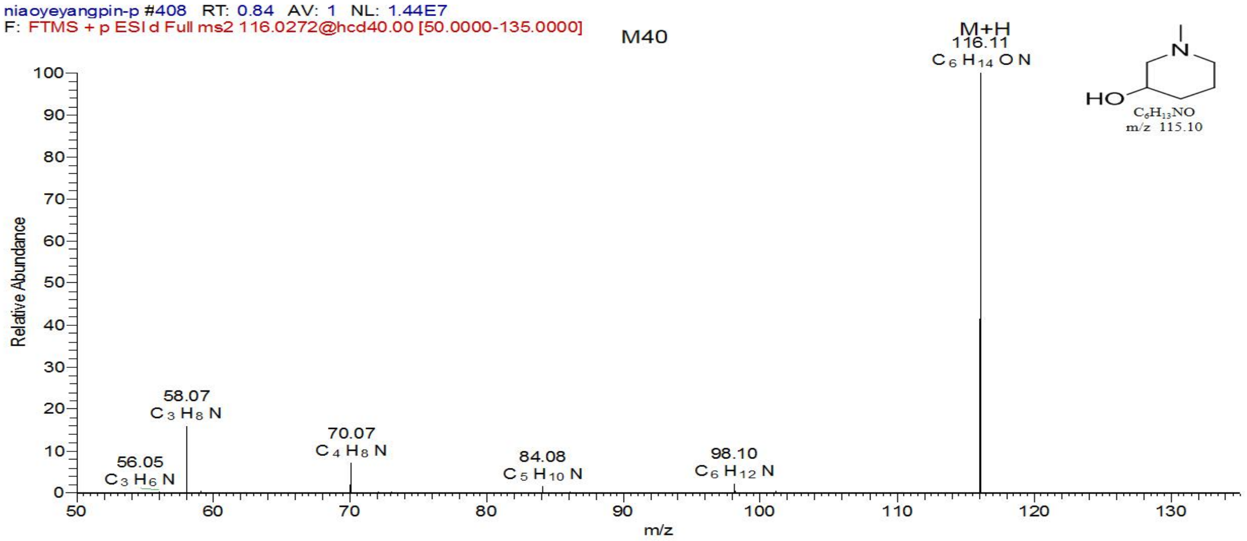
niaoyeyangpin-p #408 RT: 0.84 AV: 1 NL: 1.44E7
F: FTMS + p ESI d Full ms2 116.0272@hcd40.00 [50.0000-135.0000]
M40
M+H
116.11
C6 H14 O N
58.07
C3 H8 N
56.05
C3 H6 N
70.07
C4 H8 N
84.08
C5 H10 N
98.10
C6 H12 N
Relative Abundance
m/z
HO
N
C6H13NO
m/z 115.10

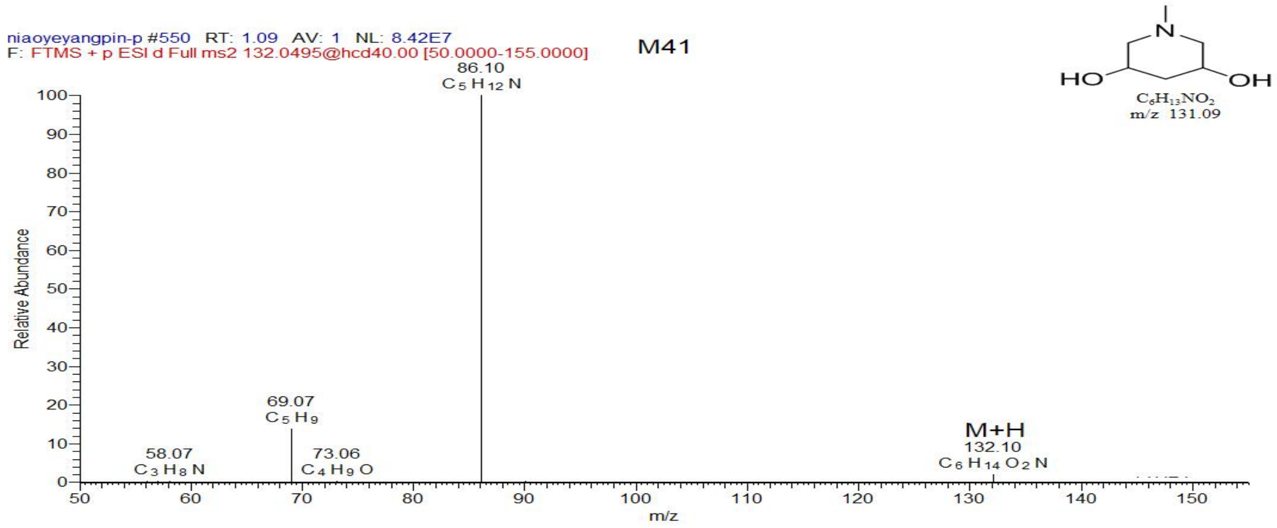
niaoyeyangpin-p #550 RT: 1.09 AV: 1 NL: 8.42E7
F: FTMS + p ESI d Full ms2 132.0495@hcd40.00 [50.0000-155.0000]
M41
86.10
C5 H12 N
69.07
C5 H9
58.07
C3 H8 N
73.06
C4 H9 O
M+H
132.10
C6 H14 O2 N
Relative Abundance
m/z
HO
OH
N
C6H13NO2
m/z 131.09

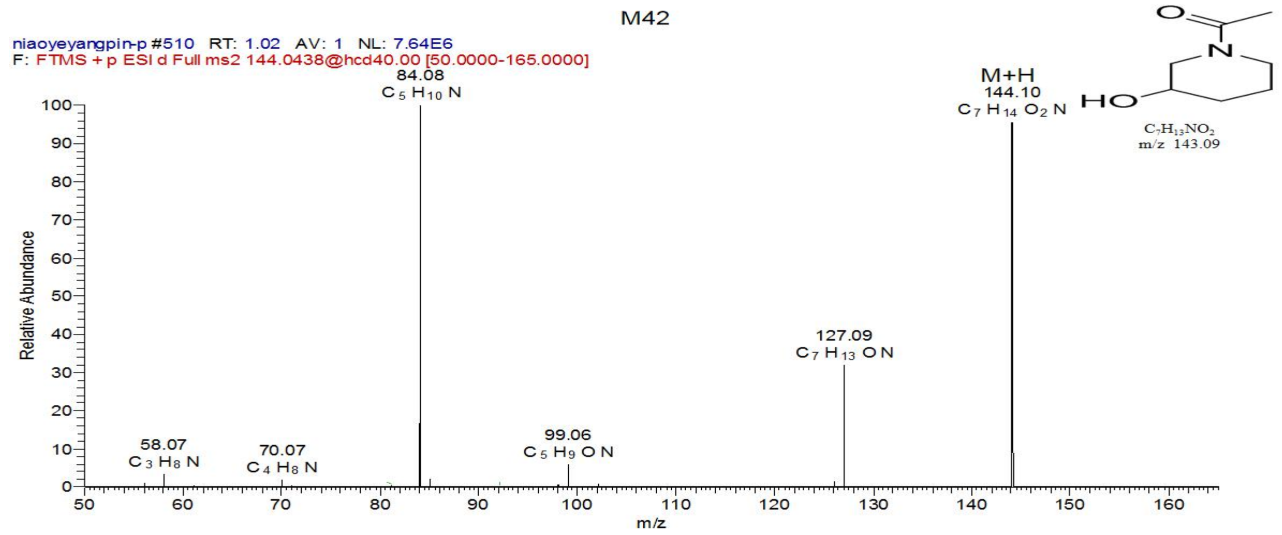
M42
niaoyeyangpin-p #510 RT: 1.02 AV: 1 NL: 7.64E6
F: FTMS + p ESI d Full ms2 144.0438@hcd40.00 [50.0000-165.0000]
84.08
C 5 H 10 N
M+H
144.10
C 7 H 14 O 2 N
127.09
C 7 H 13 O N
99.06
C 5 H 9 O N
58.07
C 3 H 8 N
70.07
C 4 H 8 N
Relative Abundance
m/z
HO
N
O
C7H13NO2
m/z 143.09

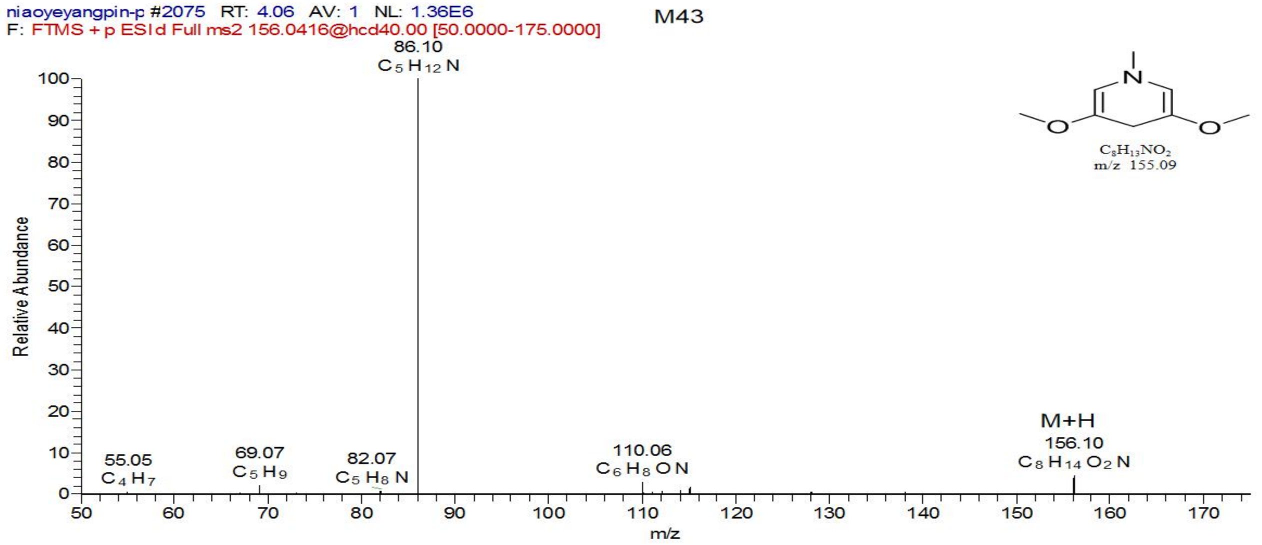
niaoyeyangpin-p #2075 RT: 4.06 AV: 1 NL: 1.36E6
F: FTMS + p ESI d Full ms2 156.0416@hcd40.00 [50.0000-175.0000]
M43
86.10
C 5 H 12 N
55.05
C 4 H 7
69.07
C 5 H 9
82.07
C 5 H 8 N
110.06
C 6 H 8 O N
M+H
156.10
C 8 H 14 O 2 N
Relative Abundance
m/z
N
O
O
C8H13NO2
m/z 155.09

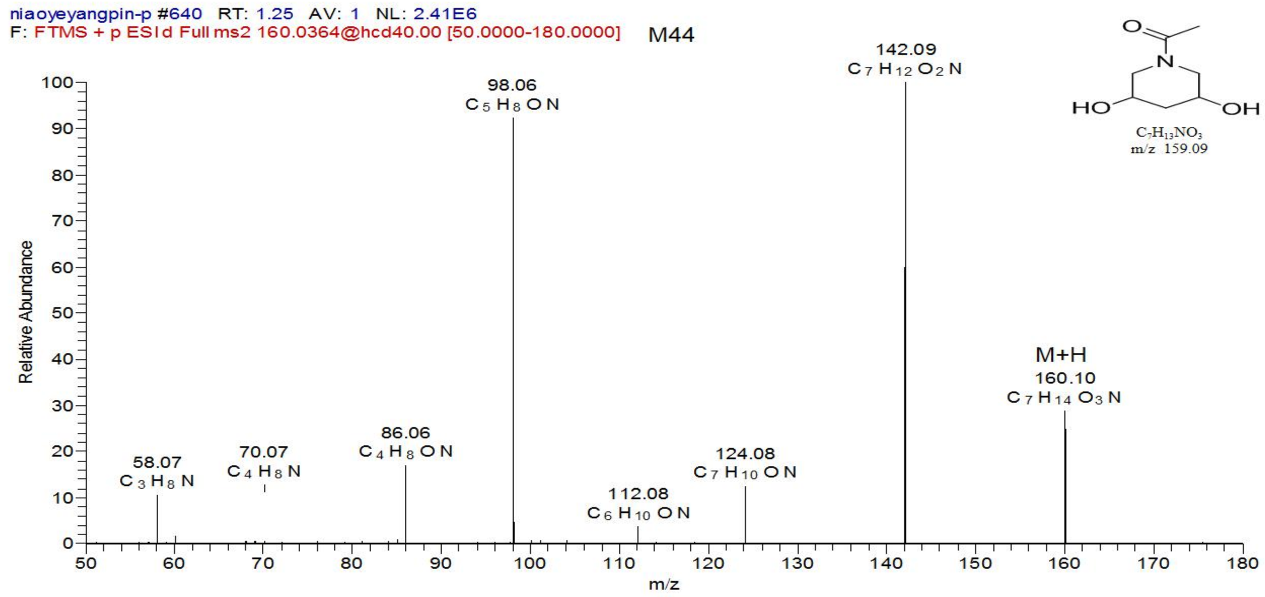
niaoyeyangpin-p #640 RT: 1.25 AV: 1 NL: 2.41E6
F: FTMS + p ESI d Full ms2 160.0364@hcd40.00 [50.0000-180.0000]
M44
142.09
C 7 H 12 O 2 N
98.06
C 5 H 8 O N
M+H
160.10
C 7 H 14 O 3 N
58.07
C 3 H 8 N
70.07
C 4 H 8 N
86.06
C 4 H 8 O N
112.08
C 6 H 10 O N
124.08
C 7 H 10 O N
Relative Abundance
m/z
O
N
HO
OH
C7H13NO3
m/z 159.09

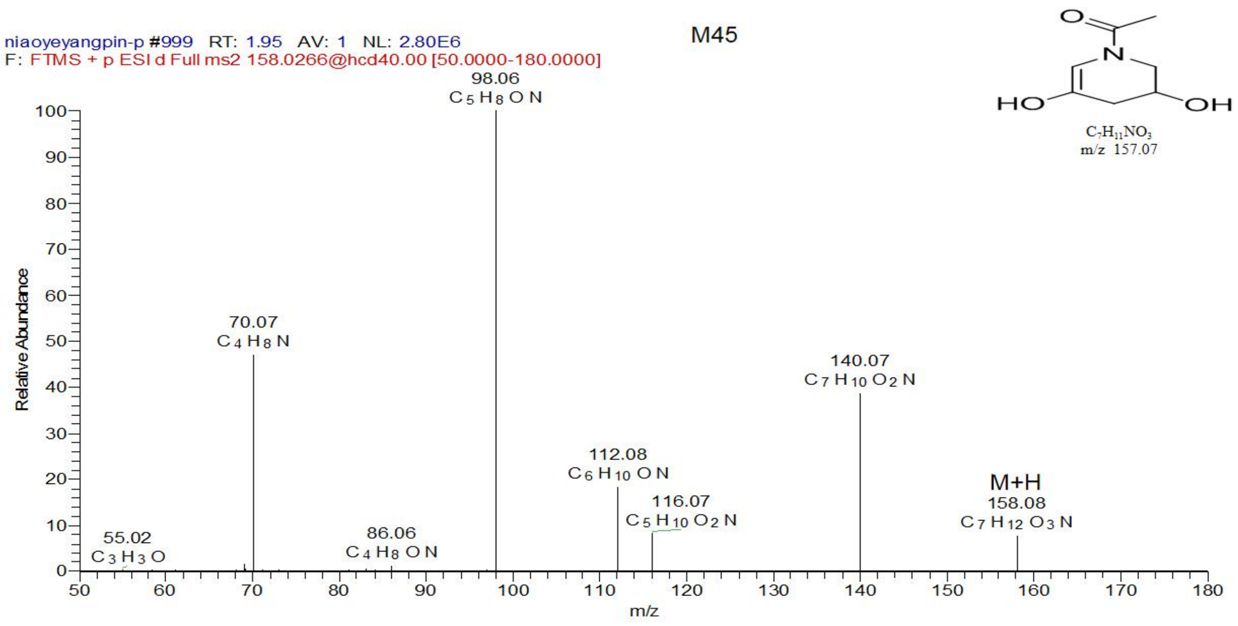


**Figure S2. LC-MS/MS spectra of troxipide metabolites (M0-M45).**

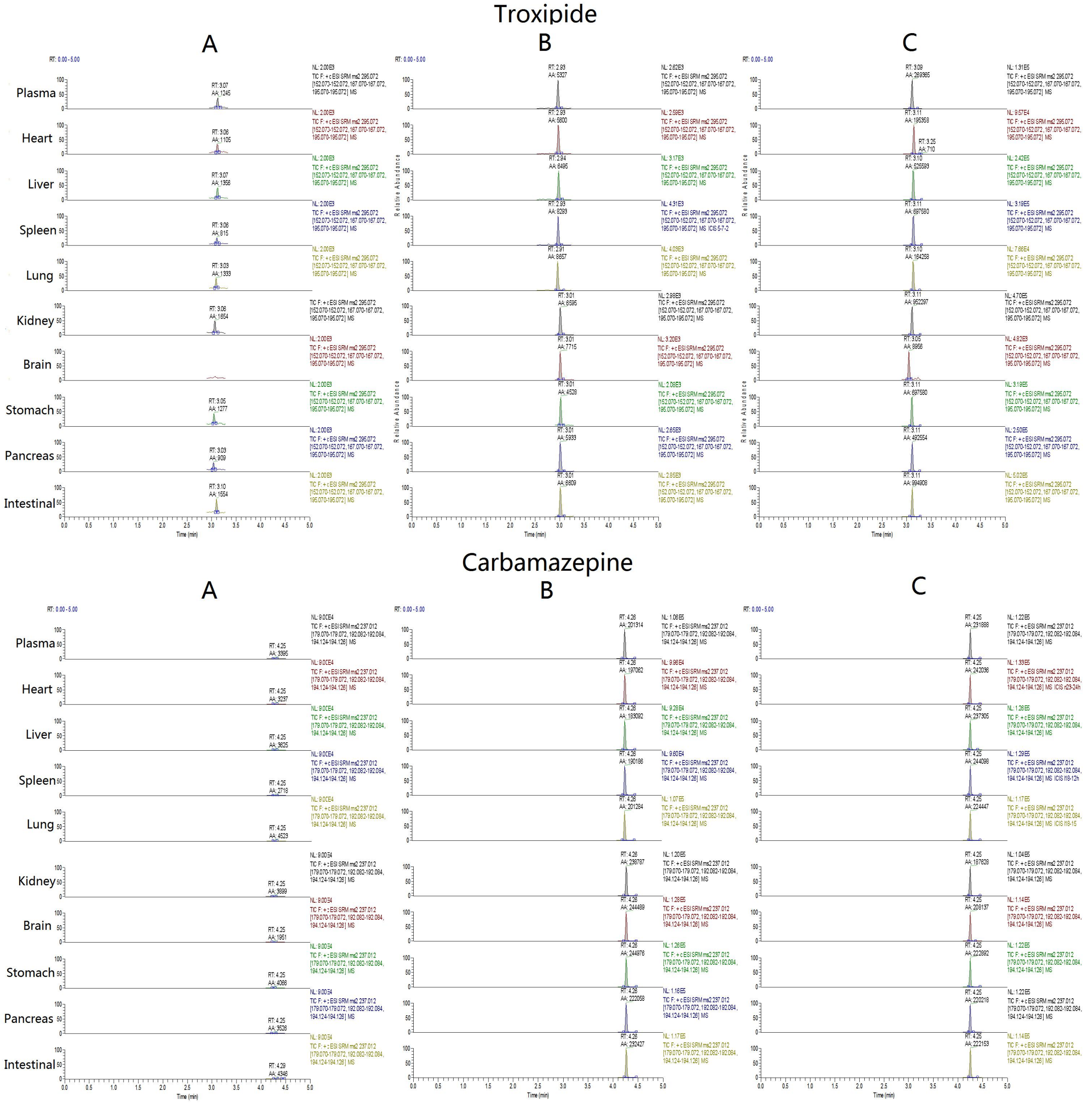


**Figure S3. Representative SRM chromatograms of troxipide and IS in the rat plasma and tissues.** (A) a blank plasma and all tissues sample; (B) a blank plasma and all tissue sample spiked with troxipide (LLOQ, 5 ng/mL); (C) plasma and all tissue sample 60 min after administration of single dosage 40 mg/kg.

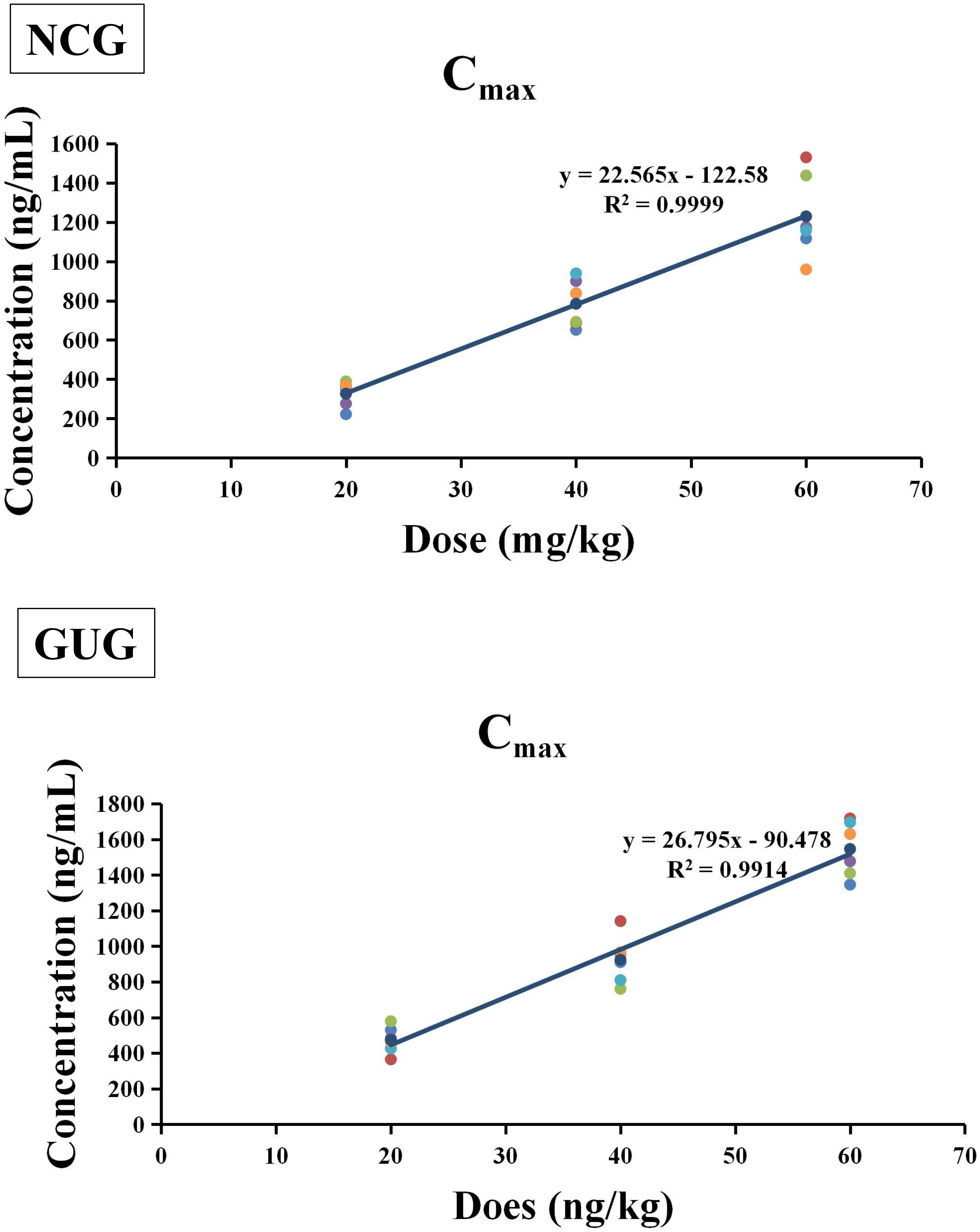


**Figure S4 A linear relationship between drug $C_{max}$ to dose in vivo in rats.**

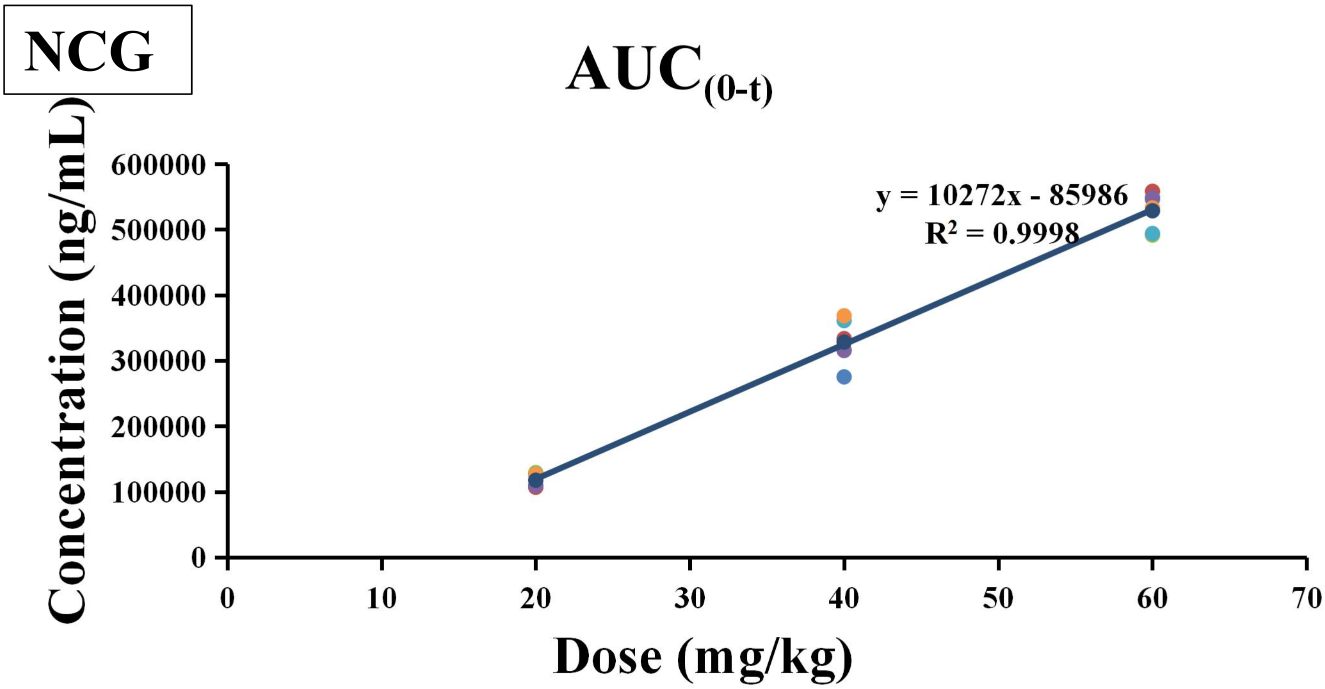


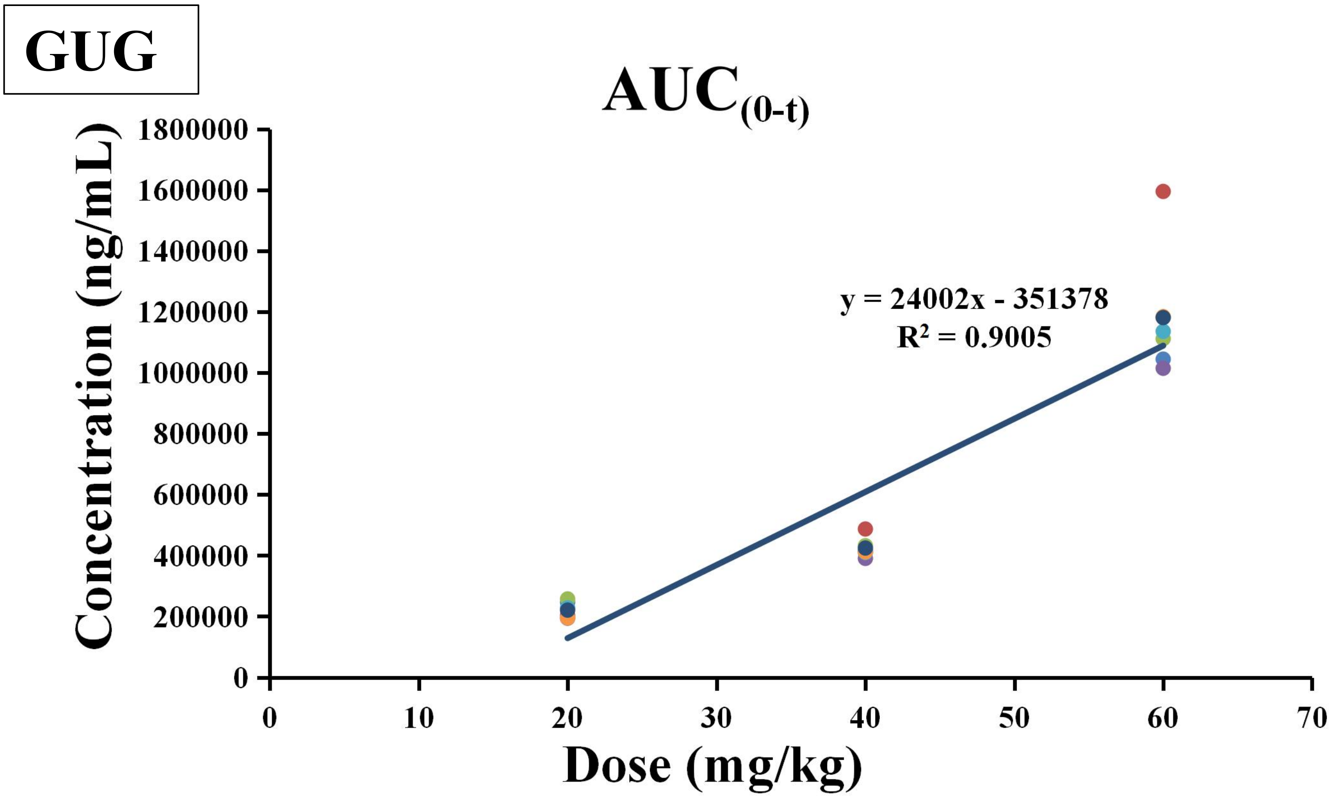


**Figure S5 A linear relationship between drug $AUC_{(0\text{-}t)}$ to dose in vivo in rats.**

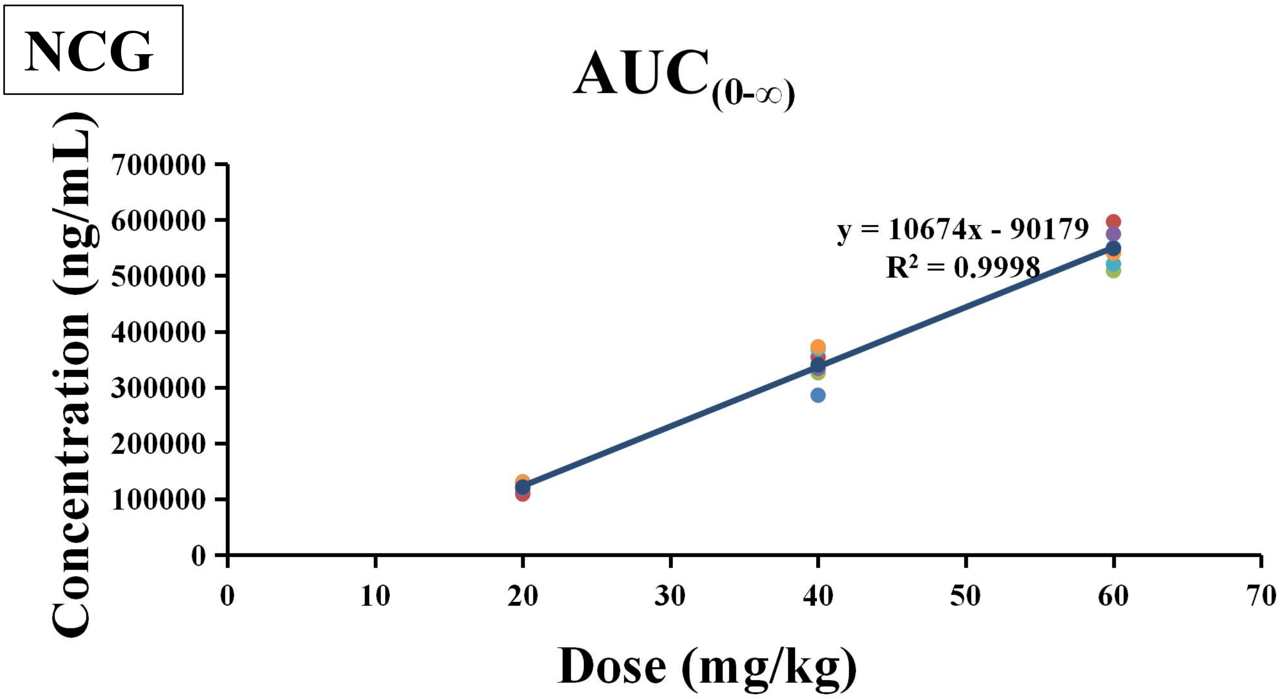


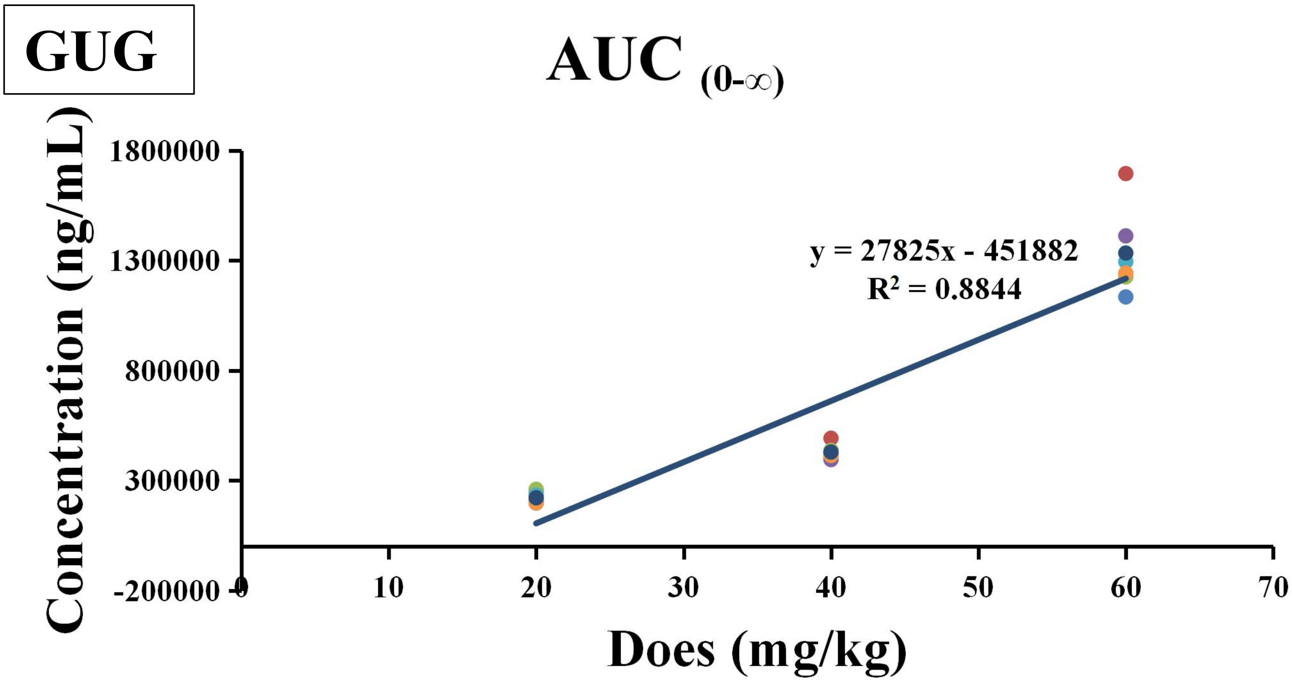


**Figure S6 A linear relationship between drug AUC$_{(0-\infty)}$ to dose in vivo in rats.**

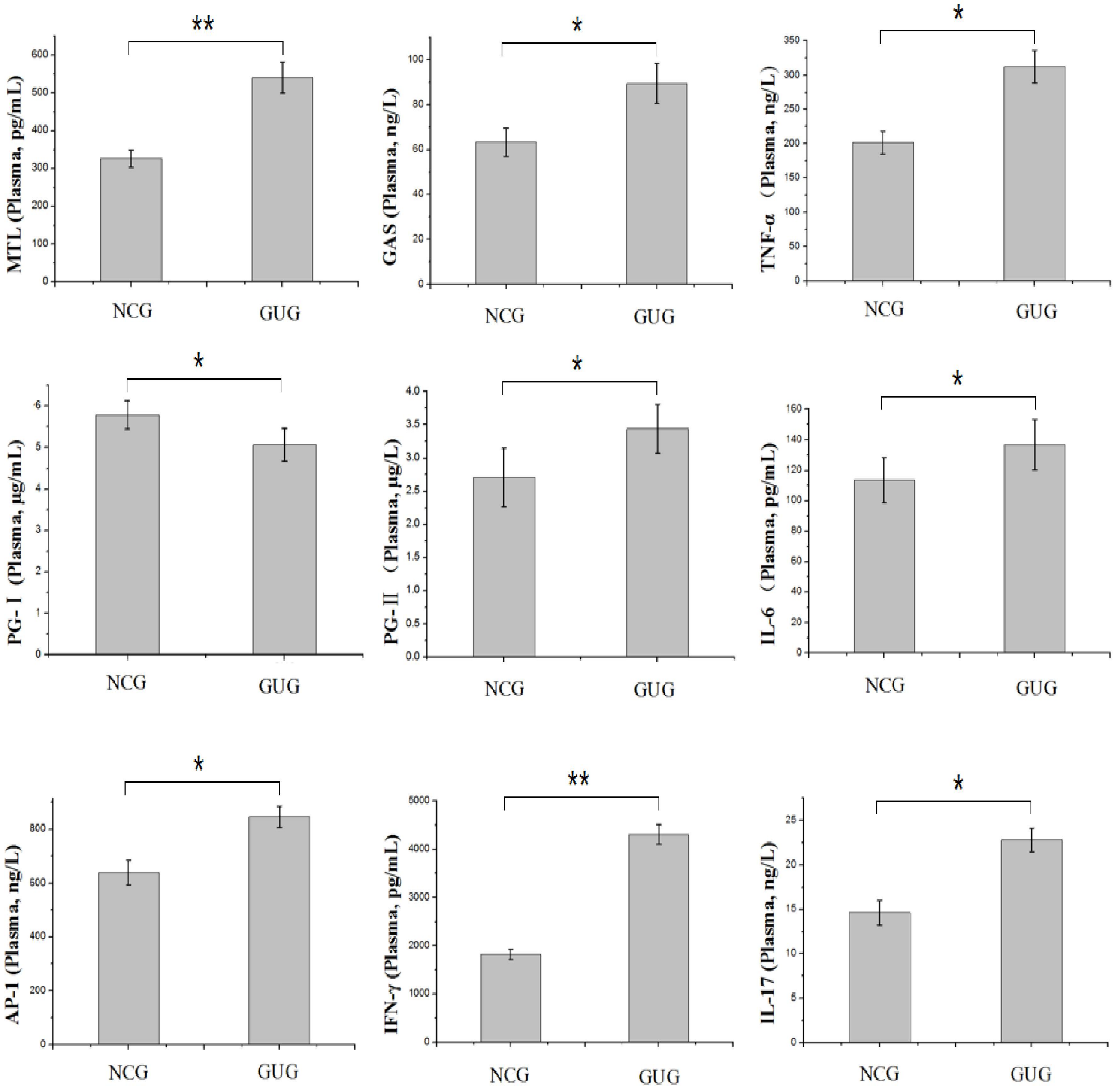


**Figure S7. MTL, GAS, TNF-α, PG-I , PG-II, IL-17, IFN-γ, AP-1 and IL-6 levels in the rat plasma before troxipide treatment.** NCG, normal group (0.9% normal saline 10 mL kg-1 day-1); GUG, gastric ulcer group (5% acetic acid 10 mL kg-1 day-1). Values are presented as means ± SD for all groups (n = 10). *P < 0.05 and **P < 0.01 indicate statistically significant differences when the GUG is compared with the NCG.

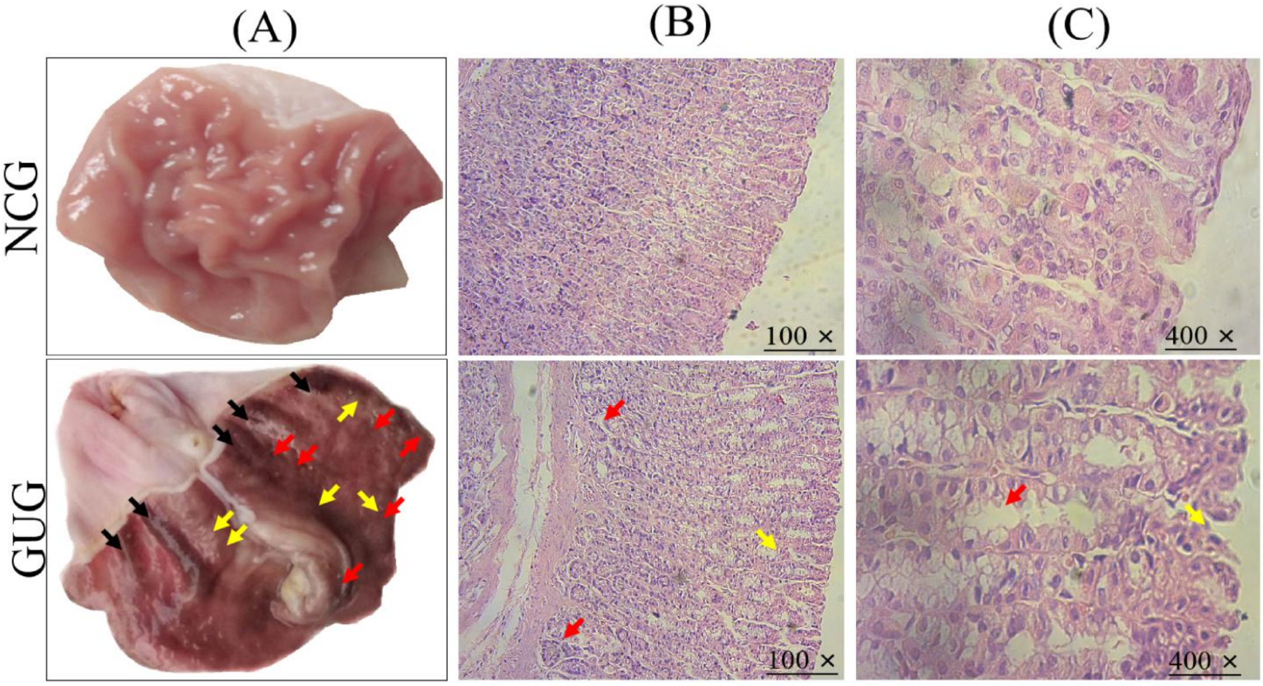


**Figure S8. Macroscopic and microscopic analysis of stomach tissue before troxipide treatment.** (A) Macroscopic analysis of GU before troxipide treatment. (B) Sections of the gastric mucosa (H&E staining) (100×). (C) Sections of the gastric mucosa (H&E staining) (400×). NCG, normal group (0.9% normal saline 10 mL kg-1 day-1); GUG, gastric ulcer group (5% acetic acid 10 mL kg-1 day-1).

# Supplementary Results and Discussion

## Metabolic study

1. Identification of troxipide metabolites in feces

The molecular ion of metabolite 1 (M1), metabolite 2 (M2), metabolite 3 (M3) were respectively m/z 281.15 ($C_{14}H_{20}N_2O_4$), 267.13 ($C_{13}H_{18}N_2O_4$) and 253.12 ($C_{12}H_{16}N_2O_4$) and were eluted at 3.45, 0.81 and 1.08 min. The product ions of m/z 281.15 (M1) weree m/z 181.15 ($C_9H_9O_4$) and 84.08 ($C_5H_{10}N$), and all 14 Da less than m/z 295.16 (M0) and its product ions were m/z 195.07 ($C_{10}H_{11}O_4$) and 84.08 ($C_5H_{10}N$). Similarly, the product ions of m/z 267.13 (M2) and 253.12 (M3) were all 14 Da less than 281.15 ($C_{14}H_{20}N_2O_4$) and 267.13 ($C_{13}H_{18}N_2O_4$). The results of above indicated that M1, M2 and M3 was probably demethylation product of M0.

Metabolite 5 (M5) with m/z 393.30 ($C_{24}H_{41}O_4$) was separated at 9.54 min in $[M+H]^+$ ion mode of which elemental composition without nitrogen atoms and the degree of unsaturation was 5, indicated that the amide group  was broken on the M0, and the group was completely removed with amino group. According to the fragmentation regular of M5, the MS/MS characteristic ions of M4 were 123.04 ($C_7H_7O_2$) and 375.33 ($C_{24}H_{39}O_3$). M5 was a product which was demethylated, reducted and palmitoyl conjugated of M0 by Mass Frontier verification.

Metabolite 6 (M6) at m/z 181.07 ($C_9H_{12}NO_3$) was tested at 1.12 min with $[M+H]^+$ ion which its degree of unsaturation was 5 and product ions were m/z 123.04 ($C_7H_7O_2$) and 136.08 ($C_8H_{10}NO$), and its elemental composition loss a methoxyl compared to M4. Based on these data and Mass Frontier verification, M6 was a product which loss pyridine, demethylation and dehydration of M0.

Metabolite 7 (M7) at 1.74 min with $[M+H]^+$ ion was observed at m/z 166.09 ($C_9H_{12}NO_2$), of which the composition was less one oxygen atom (O) than the M6. Under high-energy collision, the product ion m/z 166.09 ($C_8H_{10}N$) was formed due to less 16 Da than M6. The results suggested the M7 was a product which was oxidation, dehydration and methylation of M0.

Metabolite 9 (M9) was separated at 0.88 min with m/z 116.07 ($C_5H_{10}NO_2$) at $[M+H]^+$ ion, of which the unsaturation degree was 3. Thus, the major fragment ions were m/z 70.07 ($C_4H_8N$) and 88.08 ($C_4H_{10}NO$).

Based on data, it was inferred that M9 was the product derived from oxylation of the M8. By Mass Frontier verification to fragments ion of M9, it was proofed that M9 was oxidation and hydration on the M0.

Metabolite 10 (M10) with m/z 130.09 ($C_6H_{12}NO_2$) was presented at 0.89 min in $[M+H]^+$ ion mode, which was 14 Da more than M9 and was judged to be a methylated product of M9 by Mass Frontier verification. The product ions M10 were at m/z 84.08 ($C_5H_{10}N$) and 56.05 ($C_3H_6N$). Based on these data, M10 was a product of oxidation and methylation on the M0.

Metabolite 11 (M11) at $[M+H]^+$ ion was diagnosed at 0.91 min with m/z 118.09 ($C_5H_{12}NO_2$), of which the accurate mass was 2 Da more than M9 and its fragment ions was 72.08 ($C_4H_{10}N$). Thence, by Mass Frontier and verification, M11 was proposed to be the metabolite which was obtained by M9 reduction. However, the specific reduction position may be two reduction pathway which were the carbonyl group or that of the imino group. Overall, M11 may be oxidation, hydration and reduction on the M0. Meantime, M11 may be also Oxidative deamination to alcohol and hydration product of M0.

Metabolite 12 (M12) was separated at 0.80 min with m/z 131.12 ($C_6H_{15}N_2O$) at $[M+H]^+$ ion mode, of which the molecular ions were m/z 114.09 ($C_6H_{12}NO$) and 72.08 ($C_4H_{10}N$). According to the composition of the elements and the regulation of molecular cleavage, M12 was produced by oxidation, methylation and hydration of M0.

Metabolite 13 (M13) was detected at the retention 0.80 min. They showed an accurate $[M+H]^+$ ion at m/z 143.12 ($C_7H_{15}N_2O$), which suggested the M13 was increased 42 Da compared with the protonated molecular ion of M8. So, the M13 could be a oxidation aand acetylation metabolite of M0. Meantime, the product ions of 14 was 84.08 ($C_5H_{10}N$) which was a piperidine ring. The M13 was more 59 Da than piperidine ring. So, the M13 also could be Glycine Conjugation metabolite of piperidine ring which was a Nitro-reduction product of the M0.

Metabolite 14 (M14) with 0.80 min at $[M+H]^+$ ion was detected at 175.11 ($C_7H_{15}N_2O_3$) of which the degree of unsaturation was 2 and the fragmentation ions were m/z 70.07 ($C_4H_8N$) and 112.08 ($C_6H_{10}NO$). The molecular formula of M14 was equivalent to a composition of $C_2H_3NO$ less than the M11. By Mass Frontier and

verification, M14 was assumed to be the metabolite which was oxidative deamination to alcohol and glycine conjugated of M0.

Metabolite 15 (M15) was diagnosed at 3.65 min with $[M+K]^+$ ion at m/z 295.16 ($C_{11}H_{24}N_6OK$) of which the fragmentation ions were m/z 195.07 ($C_6H_{12}N_4OK$) and 84.08 ($C_5H_{10}N$), suggested the structure of M15 contained a piperidine ring. According to element composition and cracking law of parent ion, M16 should be the oxidation and arginine conjugation product of M0.

2. Identification of troxipide metabolites in feces

Metabolite 16 (M16) was found at 3.63 min with m/z 161.05 ($C_9H_8NO_2$) at $[M+H]^+$ ion that its degree of unsaturation was 7 and product ions were m/z 105.03 ($C_7H_5O$) and 134.06 ($C_8H_8NO$). Because of the hydroxyl group on the aromatic ring dose not readily change to a carbonyl group and the degree of unsaturation of the benzene ring was 4, the methoxy group was completely removed on the benzene ring. Based on analysis of the data and verification of Mass Frontier, M16 was suggested demethylation, dehydration and oxidation of M0.

Metabolite M17 (M17) had a retention time of 4.171 min and showed $[M+H]^+$ ion at m/z 309.14449 ($C_{15}H_{21}N_2O_5$), and the characteristic product ions of M17 were 195.07 ($C_{10}H_{11}O_4$) and 98.06 ($C_5H_8NO$), which was more 14Da than M0. And then , the unsaturation degree of M17 was 7. Based on these data, it had been verified that M18 was obtained by desaturation and oxidation on the basis of M0.

Metabolite M18 (M18) at m/z 457.18 ($C_{20}H_{29}N_2O_{10}$) with $[M+H]^+$ ion was eluted at 2.92 min and its product ions were m/z 84.08 ($C_5H_{10}N$ ), 181.05 ($C_9H_9O_4$) and 281.15 ($C_{14}H_{21}N_2O_4$). The M18 was more 176 Da compared with 281.15 ($C_{14}H_{21}N_2O_4$) of M1 and the fragmentation pathways of M18 and M1 were similar. The above results suggests that M18 could be formed by demethylation and glucoside conjugation of M0.

Metabolite M19 (M19) showed with $[M+H]^+$ ion at m/z 287.16 ($C_{13}H_{23}N_2O_5$) at 3.26 min, of which degree of unsaturation was 4. The results suggests piperidine ring has been broken and carbonyl group was reduced in M0 structure. The elemental composition of M19 was compared with M0 loss two carbon atoms and increase one hydroxyl group and its product ions were 115.09 ($C_5H_{11}N_2O$) and 175.11 ($C_7H_{13}N_2O_2$) which have been

verified by mass measurements. So, Metabolite M19 was confirmed as demethylation, reduction and hydration of M0.

Metabolite 20 (M20) was shown in urine at m/z 311.16 ($C_{15}H_{23}N_2O_5$) with $[M+H]^+$ ion at 2.97 min, of which degree of unsaturation was 6 and product ions were m/z 84.05 ($C_5H_{10}N$), 140.07 ($C_7H_{10}NO_2$) and 269.15 ($C_{13}H_{21}N_2O_4$). The m/z 269.15 ($C_{13}H_{21}N_2O_4$) was acquired by metabolite 20 loss deacetylation ($C_2H_2O$). The m/z 269.15 ($C_{13}H_{21}N_2O_4$) was increased 2D compared with M2, of which the results suggested metabolite 20 proceed  carbonyl reduction and acetylation on the basis of metabolite 2. Ultimately, it is shown that M20 was obtained by M0 demethylation, carbonyl reduction and N-acetylation.

Metabolite 21 (M21) with $[M+H]^+$ ion m/z 297.14 ($C_{14}H_{21}N_2O_5$) was detected at 2.11 min, and its degree of unsaturation was 6 and product ions were m/z 84.08 ($C_5H_{10}N$), 126.05 ($C_6H_8NO_2$) and 192.10 ($C_{11}H_{14}NO_2$) which was lost methylene compared with M20. However, since the molecular ion dissociation mode of M21 was different from that of M20, it was presumed that the site of acetylation was different from that of M21. It was verified by Mass Frontier simulation of the dissociation process that it was demethylated, reduced and N-acetylated on the amide by M0.

Metabolite 22 (M22) at m/z 251.13 ($C_{13}H_{18}N_2O_3$) with $[M+H]^+$ ion was eluted at 3.39 min, of which its degree of unsaturation was 6 and product ions were m/z 84.04 ($C_4H_6NO$), 105.03 ($C_7H_5O$) and 188.11 ($C_{12}H_{14}NO$). According to the above data information and Mass Frontier verification, M22 was a metabolite of demethylation, dehydration and hydration on the basis of M0.

Metabolite 23 (M23) with m/z 252.16($C_{14}H_{22}NO_3$) at $[M+H]^+$ ion was detected at 4.31 min. The product ions of M23 were m/z 149.08 ($C_9H_{11}NO$) and 206.15 ($C_{13}H_{20}NO$). Due to the degree of unsaturation was 5 and the elemental composition of M23, it can be judged that the piperidine ring has been broken and oxidative deamination to alcohol. It is finally concluded that the structure of M23 may be due to demethylation, oxidantion, methylation of M0.

Metabolite 24 (M24) was eluted at 4.90 min with m/z 303.10 ($C_{12}H_{19}N_2O_5S$) at $[M+H]^+$ ion. The unsaturation degree of M24 was 5 and product ions were m/z 146.06 ($C_9H_8NO$) and 174.05 ($C_{10}H_8NO_2$). The data shown a conclusion which metabolite may be a disrupted piperidine or a reduced carbonyl group. In addition, because

of the elemental composition of M24, it can be infer that M24 may be a sulfonate. Based on these data and Mass Frontier verification, Metabolite 24 was a metabolite of demethylation, reduction and N-sulfation on the basis of M0.

Metabolite 25 (M25) at m/z 181.07 ($C_9H_{12}NO_3$) was tested at 3.46 min with $[M+H]^+$ ion, of which its degree of unsaturation was 5 and product ions were m/z 110.06 ($C_6H_8NO$) and 136.08 ($C_8H_{10}NO$). And, its elemental composition was loss 30 Da ($CH_2O$) compared to M4. But, Based on these data and Mass Frontier verification, Metabolite 25 was a metabolite of demethylation, dehydration and methylation after losing pyridine of M0.

Metabolite 26 (M26) was discovered at 4.11 min with m/z 180.10($C_{10}H_{14}NO_2$) in $[M+H]^+$ ion mode and its unsaturation was 6 and product ions were m/z 105.03 ($C_7H_5O$) and 152.11 ($C_9H_{14}NO$). The elemental composition of M26 was two O less than M4. According to the above data analysis and verification of Mass Frontier, Metabolite 26 was inferred a metabolite of demethylation, dehydration, desaturation and methylation on the basis of M0.

Metabolite 27 (M27) with $[M+H]^+$ ion at m/z 170.08 ($C_8H_{12}NO_3$) was verified at 1.04 min. The unsaturation of M27 was 4 and product ions were m/z 152.07 ($C_8H_{10}NO_2$) and 134.06 ($C_8H_8NO$). According to the elemental composition, the M27 was loss $C_7H_{11}NO_2$ (125Da) than M0. The results suggested the piperidine of M0 was loss and the carbonyl of M0 was destroyed. Based on the above data and Mass Frontier verification, Metabolite 27 was a metabolite of demethylation, desaturation and methylation on the basis of M0.

Metabolite 28 (M28) with m/z 170.08 ($C_8H_{12}NO_3$) at $[M+H]^+$ ion was proved at 3.25 min, of which the unsaturation of M28 was 5 and product ions were m/z 110.06 ($C_6H_8NO$) and 134.06 ($C_8H_8NO$). The elemental composition of M28 was less two methoxy group than M4. Based on the above data and Mass Frontier verification, Metabolite 28 was a metabolite of demethylation, dehydration and methylation after losing pyridine of M0.

Metabolite 29 (M29) was detected at 2.11 min with m/z 297.14 ($C_{14}H_{21}N_2O_5$) in $[M+H]^+$ ion mode and its product ions were m/z 192.10 ($C_{11}H_{14}NO_2$) and 255.13 ($C_{12}H_9N_2O_4$), and degree of unsaturation was 6. After multiple analysis of the data and verification of Mass Frontier, M29 was a product of Glutamine Conjugation.

Finally, it was speculated that M29 was a demethylation, reduction, and Glutamine Conjugated product after M0 amide bond cleavage.

Metabolite 30 (M30) was identified at $[M+H]^+$ ion at 1.63min with m/z 100.11 ($C_6H_{14}N$) that its product ions were m/z 55.05 ($C_4H_7$) and 83.09 ($C_6H_{11}$), and its degree of unsaturation was 1. In summary, Metabolite 30 was indicated a methylated piperidine ring.

Metabolite 31 (M31) was observed at m/z 100.08 ($C_5H_{10}NO$) with $[M+H]^+$ ion at 1.79 min which its degree of unsaturation was 2 and product ions were m/z 56.05 ($C_3H_6N$) and 72.08 ($C_4H_{10}N$). According to elemental composition and verification of Mass Frontier, Metabolite 31 can be postulated to be formed through oxidative Deamination to Ketone at M8 in vivo.

Metabolite 32 (M32) with m/z 245.16 ($C_{10}H_{21}N_4O_3$) was separated at 1.03 min in $[M+H]^+$ ion mode which its degree of unsaturation was 3 and product ions were m/z 70.07 ($C_4H_8N$) and 158.08 ($C_7H_{12}NO_3$). The elemental composition of M32 were 144Da more than those of M8. By Multiple analysis of known data and Mass Frontier verification, Metabolite 32 was proved that reduction, glutamine conjugation with M0.

Metabolite 33 (M33) was observed at 4.37 min with m/z 291.12 ($C_{11}H_{19}N_2O_7$) at $[M+H]^+$ ion of which elemental composition was more 190 Da ($C_6H_7O_7$) than M8. Its degree of unsaturation was 4 and product ions were m/z 162.04 ($C_5H_8NO_5$) and 273.11 ($C_{11}H_{17}N_2O_6$). After multiple speculations and Mass Frontier verification, Metabolite 33 was confirmed to be the product of M0 reduction and glucuronide conjugation.

Metabolite 34 (M34) with $[M+H]^+$ ion was appeared m/z 244.12 ($C_{11}H_{18}NO_5$) at 2.78 min, of which its product ions were m/z 84.08 ($C_{11}H_{14}NO_3$), 100.08 ($C_5H_{10}NO$) and 208.10 ($C_{11}H_{14}NO_3$). The product ion m/z 84.08 was similar to fragment of M8. The unsaturation degree of M34 was 4, and its elemental composition and fragments in the MS/MS spectrum could be predicted that the amino group had been lost on the piperidine ring. According above stated and Mass Frontier verification, M34 was predicted to be metabolite of oxidation and glucoside conjugation after the amide bond cleavage of M0.

Metabolite 35 (M35) was identified at m/z 156.07 ($C_7H_{10}NO_3$) with $[M+H]^+$ ion at 1.00 min, of which unsaturation degree was 5. The product ions of M35 were 138.05 ($C_7H_8NO_2$) and 110.06 ($C_6H_8NO$).

According to its elemental composition and fragments in the MS/MS spectrum, the M35 could be predicted that it was oxidation and glycine conjugation after amide bond cleavage of M0.

Metabolite 36 (M36) was separated at 1.12 min with $[M+H]^+$ at m/z 231.13 ($C_{10}H_{18}N_2O_4$), of which its product ions were 126.09 ($C_7H_{12}NO$) and 84.08 ($C_5H_{10}N$), and unsaturation degree was 3. According to its fragments in the MS/MS spectrum and Mass Frontier verification, the M36 could be produced by oxidation and ornitine conjugation after amide bond cleavage of M0.

Metabolite 37 (M37) with $[M+H]^+$ ion at m/z 128.07 ($C_6H_{10}NO_2$) was eluted at 1.19 min of which product ions were at m/z 84.08 ($C_5H_{10}N$) and m/z 56.05 ($C_3H_6N$) were found to be similar to similar fragmentation spectra of M10 and the $[M+H]^+$ was 2 Da less than that of M10. Based on the unsaturation degree of M36, it was speculated that M37 was formed oxidation of hydroxyl group on M10 by Mass Frontier verification.

Metabolite 38 (M38) was detected at m/z 146.09 ($C_6H_{12}NO_3$) with $[M+H]^+$ ion at 0.86 min of which accurate mass was 30 Da higher than M9 and unsaturation degree was 2. According to element composition of M38 and Mass Frontier verification, it was deduced to be the product of M9 N-Methoxylation.

Metabolite 39 (M39) at 1.82 min was found at m/z 140.07 ($C_7H_{10}NO_2$) with $[M+H]^+$ ion of which the mass of characteristic fragment ions were m/z 70.07 ($C_4H_8N$) and 98.06 ($C_5H_8NO$) which less than 2 Da compared to m/z 72.08 ($C_4H_{10}N$) and 100.08 ($C_5H_{10}NO$) of M31 fragmentions, suggested that the part of M39 structure was formed by reduction of M31. By comparing element composition and Mass Frontier verification, the mass of the remaining part (42Da) was the mass of the acetyl group. Based on the above data, M39 should be the oxidative deamination to ketone and acetylation product after amide bond cleavage of M0.

Metabolite 40 (M40) at m/z 116.11 ($C_6H_{14}NO$) was detected at 0.84 min with $[M+H]^+$ ion that its unsaturation degree was 1 and the mass of characteristic fragment were m/z 58.07 ($C_3H_8N$), 84.08 ($C_5H_{10}N$) and 98.10 ($C_6H_{12}N$). The molecular ion of M40 at m/z 98.10 ($C_6H_{12}N$) may be due to the loss a molecule water from the parent ion. The molecular ion at m/z 84.08 ($C_5H_{10}N$) may be a piperidinyl group which can be attributed to the decrease a methyl group relative to the fragment ion at m/z 98.10 ($C_6H_{12}N$). Thus, M40 was provided a product which was obtained by oxidation deaminated to alcohol and methylation after amide bond cleavage of M0.

Metabolite 41 (M41) was revealed at 1.09 min with m/z 132.10 ($C_6H_{14}NO_2$) in $[M+H]^+$ ion mode, which was 16 Da (O) greater than M40. The M41 product ions were m/z 86.10 ($C_5H_{12}NO$) and m/z 69.07 ($C_5H_9$).According to unsaturation degree and element composition, M41 was supposed as oxidative deamination to alcohol, methylation and oxidation product after amide bond cleavage of M0 by Mass Frontier verification.

Metabolite 42 (M42) was disclosed at m/z 144.10 ($C_7H_{13}NO_2$) with $[M+H]^+$ ion at 1.02 min of which product ions were showed at m/z 84.08 ($C_5H_{10}N$) and 127.09 ($C_7H_{13}NO$) suggested the parent nucleus of M42 was piperidinyl (m/z 84.08). Because of the elemental composition and cracking law, the m/z 144.10 ($C_7H_{13}NO_2$) was loss 17 Da (OH) led to produce of fragment ion m/z 127.09 ($C_7H_{13}NO$), which indicated to loss a hydroxyl group. The fragment ions at m/z 84.08 ($C_5H_{10}N$) was 43 Da ($C_2H_3O$) less than ion at m/z 127.09 ($C_7H_{13}NO$), which showed to remove an acetyl group. Based on the above analysis data and Mass Frontier verification, it was conjectured that M42 was a metabolite to be oxidative deaminated to alcohol and acetylation after amide bond cleavage of M0.

Metabolite 43 (M43) with $[M+H]^+$ ion at m/z 156.10 ($C_8H_{14}NO_2$) was displayed at 4.06 min of which fragment ions were 86.10 ($C_5H_{12}N$) and 110.08 ($C_5H_8NO$). According to unsaturation and elemental composition, M45 was judged a product which was oxidation and methylation on the M0.

Metabolite 44 (M44) at m/z 160.09($C_7H_{14}NO_3$) was showed at 1.25 min with $[M+H]^+$ ion of which fragment ions were m/z 98.06 ($C_5H_8NO$) and 142.09 ($C_7H_{12}NO_2$). A difference of 16 Da (O) mass units compared with that of M42 combined with the elemental composition indicated that M44 could be formed by oxidation of M42. By Mass Frontier and unsaturation degree verification, The structure of M44 was acquired by oxidation deaminated to alcohol, acetylation and oxidation after amide bond cleavage of M0.

Metabolite 45 (M45) was collected at 1.95 min with m/z 158.07 ($C_7H_{12}NO_3$) in $[M+H]^+$ ion mode of which was 2 Da less than that of M44. Therefore, M45 was speculated as a oxidation deaminated to alcohol, acetylation oxidation and desaturation product after amide bond cleavage of M0 by Mass Frontier verification.

**Pharmacokinetics and tissue distribution study**

Method validation

1. Specificity and LLOQ

The specificity of the methods was verified by comparing blank samples (plasma and tissue), blank samples spiked with troxipide and IS working solution, and samples that were collected after drug administration. Due to the efficient sample preparation and high selectivity of selective reaction monitoring (SRM), the results showed that endogenous factors in the plasma and tissue did not interfere with the detection of troxipide and IS (Fig. S3) at the RT. By comparing the area and height of the signals, we found that the lower limit of quantitation (LLOQ, 5 ng/mL) signal was 5 times greater than the blank plasma and tissue sample signals. The accuracy and precision of the LLOQ was within 20%. Moreover, all biological sample signals were more than 10 times that of the blank plasma and tissue sample signals. According to European Medicine Agency (EMA) guideline, this methods could be applicable to pharmacokinetics study.

2. Calibration Curve

The LLOQ was detected at 5 ng/mL for troxipide. The good linearity for samples (plasma and tissue) was evaluated in range of 5-10000 ng/mL, and the calibration curve of plasma was y=0.0035x+0.0029, R2=0.9999. The results was summarized in Table S1, which meet Food and Drug Administration (FDA) and EMA guidelines requirements.

3. Accuracy and precision

The intra-day and inter-day accuracy and precision of troxipide in plasma and stomach was presented in Table S2 at three concentration QC levels (15, 400, 8000 ng/mL), of which all results were within the acceptable range. The intra-day and inter-day accuracy (RE, %) for troxipide were within the range of -4.3 % to 9.4 %, and the precision (RSD, %) were ranged from 1.8 % to 13.8 % in plasma and tissue.

4. Extraction Recovery and Matrix Effect

The mean results of extraction recoveries and matrix effect were shown in Table S3 and Table S4 at three concentration QC levels. The extraction recoveries were (92.10 ± 5.70) % to (102.60 ± 5.60) %, of which the RSD were 2.07 % - 14.31 % and the RE were -12.42 % - 11.23 %. The matrix effect values for troxipide were (91.66±3.43) % to (106.39 ± 5.71) %, of which the RSD were 1.85 % - 10.67 %and the RE were -7.69 % - 6.56 %. All results of above extraction recoveries and matrix effect could meet the requirements for detecting troxipide in rats vivo, which indicated the effect of extraction methods and matrix could be negligible.

5. Stability

The stability results of troxipide was summarized in Table S5 in six replicates samples at three level QC samples in different conditions (4 h exposure at room temperature, the auto-sampler 4 ◦C for 24 h , three freeze-thaw cycles, frozen at -80 ◦C for 30 days). And, the accuracy and precision were within acceptable range, which suggested the troxipide were stable under a variety condition, and the methods could be acceptable for this study.

6. Dilution Integrity

As shown in table S6, the accuracy and precision for troxipide diluted integrity (50-dilution) were 6.9 % and -4.1 %, which meet the requirements of pharmacokinetic study. The results shown that the samples of high concentration simulated plasma could be analyzed by any of the tested dilution factors, which suggested the dilution sample had no interference detection.

7. Carryover effect

As shown in table S7, no obvious carryover for troxipide and IS were observed when the blank sample was detected subsequent to ULOQ (10000 ng/mL). And, the carryover effect results of analytes were less than 20 % of LLOQ, which indicated that the carryover did not effect accuracy and precision and could be negligible during the analysis samples.

# Supplementary Materials and Methods

## Chemicals and reagents

Troxipide (purity >98.0 %) was purchased from the Laibao Technology Co., Ltd. (Beijing, China). Carbamazepine (purity >99.99 %) of internal standard (IS) was obtained from China Institute of Food and Drug Verification (Beijing, China). Methanol and acetonitrile (UPLC-MS grade) was obtained from fisher Scientific Co., Ltd. (Pittsburgh, PA, USA). Formic acid of (LC-MS grade) was purchased from Aladdin Industrial Co., Ltd. (Shanghai, China). The ultra-pure water was prepared in our laboratory by a Milli-Q water purification system (Millipore, Bedford, MA, USA). Acetic acid glacial (analytical grade) was purchased from Tianjin Damao Chemical Reagent Co., Ltd. (Tianjin, China). The 0.22 μm membranes were obtained from Tianjin Jinteng Experimental Equipment Co., Ltd. (Tianjin, China). All other chemicals and reagents were of analytical grade and obtained commercially. Pierce Triple Quadrupole Calibration Solution TA260772 was purchased from Thermo Fisher scientific (NYSE: TMO, Rockford, USA).

## Sample preparation

1. Sample Preparation for Metabolite Identification

The plasma samples were prepared by adding acetonitrile (300 μL) to rat plasma sample (100 μL). The above mixture were then vortex-mixed for 3 min and centrifuged at 13000 r/min for 10 min at 4 °C. The supernatant was transferred into a 1.5 mL centrifuge tube and evaporated to dryness in a vacuum centrifugal concentrator. The residue was dissolved by using 50 % methanol (50 μL) and was vortex-mixed for 1 min. Finally, it was centrifuged at 13000 r/min for 5 min at 4 °C and then 5 μL supernatant injected into the UHPLC-MS/MS system.

The 200 μL of urine was added into 200 μL pure water, vortexing for 1 min. After centrifuged at 13000 rpm for 10 min at 4°C, the supernatant was filtered by the 0.20 μm organic membrane for analysis. The mashed

faeces (1.0 g) of was added to 10 mL methanol, and ultrasonic dissolved soluble substances for 15 min, and then centrifuged for 10 min at 13000 rpm. The supernatant was filtered by the 0.20 μm organic membrane for further analysis. Finally, the 5 μL sample was injected into the UHPLC-MS/MS system.

2. Sample Preparation for Pharmacokinetic Study

The one-step protein precipitation method was applied for extracting troxipide and IS. Plasma samples (90 μL) and IS (10 μL) working solution (1 μg/mL) were added into a 1.5 mL centrifuge tube. The mixture was vortexed for 2 min. Afterwards, 300 μL acetonitrile was pipetted into the above mixture for precipitating protein. The tubes were then vortex-mixed for 5 min and centrifuged at 13000 r/min for 10 min at 4 °C. The supernatant (100 μL) was then transferred into a sample bottle vial for analysis.

In the tissue distribution study, the preparation method and process for tissue homogenate samples was same as the above plasma samples description.

**Method validation**

1. Specificity

To ensure no endogenous detection of the troxipide and IS, the specificity of this methods were evaluated by comparing the chromatograms of blank samples (plasma and tissue), blank samples spiked with troxipide at lower limit of quantification (LLOQ) and IS, and the samples (plasma and tissue ) after the intravenous or intragastric administration of troxipide, respectively.

2. Calibration Curve and LLOQ

Calibration curves ($y = ax + b$) of troxipide were obtained by plotting peak area ratio (y) of troxipide to IS versus the concentrations of troxipide (x) using $1/x^2$ weighted least squares linear regression model. The criterion for calibration curve correlation coefficient ($r^2$) was at lease 0.99 or better at nine concentration levels ranging from 5 to 10000 ng/mL. In addition, the LLOQ (S/N> 10) was defined as the lowest

concentration in the calibration curve, of which the precision (expressed as relative standard deviation, RSD, %) should be within 20 % and the accuracy (expressed as relative error, RE, %) should be within ± 20 %.

3. Accuracy and precision

The accuracy and precision (plasma and stomach) were determined by evaluating three concentration level QC samples (15, 400, 8000 ng/mL) in six replicates on one day (intra-day) and three consecutive days (inter-day). In agreement with the guidance of FDA, the intra-day and inter-day precision were required within ±15 % and the acceptability criterion for accuracy was within 85 %–115 %. Especially, the precision of LLOQ should be below 20 % and the accuracy should be within 20 %.

4. Extraction Recovery and Matrix Effect

Extraction recovery was evaluated by the peak mean area ratios of blank sample (plasma and tissue) spiked with troxipide before extraction to those of spiked after extraction at three QC levels (15, 400, 8000 ng/mL) in six replicates, respectively. The matrix effect was determined by the ratio of the peak area of troxipide spiked after extraction with those of the same concentrations of the neat standard solutions at three QC levels (15, 400, 8000 ng/mL) in six replicates. The acceptability criterion of matrix factor was within 85 % - 115 %, which can be suggested that there were no matrix effects for determining biological samples.

$$\text{Recovery} = \frac{\text{peak areas of troxipide before extraction}}{\text{peak areas of troxipide after extraction}} \times 100$$

$$\text{Matrix effect} = \frac{\text{peak areas of troxipide after extraction}}{\text{peak areas of same concentrations of standard solutions}} \times 100$$

5. Stability

The stability (plasma and stomach) of troxipide was studied by analyzing six replicate samples at three QC sample (15, 400, 8000 ng/mL) under different condition: 4 h exposure at room temperature (short-term stability), the auto-sampler (4 °C) for 24 h (post-treatment stability), three freeze-thaw cycles (freeze-thaw

stability) and frozen at 80 ◦C for 30 days (long-term stability). The acceptable criteria of accuracy and precision should be within ±15 %.

6. Dilution Integrity

The dilution integrity of troxipide were studied by diluting high concentration simulated plasma (100000 ng/mL) with blank samples (plasma and tissue) to low concentration (2000 ng/mL). The accuracy and precision were evaluated by freshly prepared calibration curve at six replicate, which should be within ±15 %.

7. Carryover

The upper limit of quantification ULOQ (10000 ng/mL) was injected into HPLC-MS/MS system before injection of blank samples (plasma and tissue) for evaluating the carryover of this methods. The blank sample concentration should be below 20 % of troxipide LLOQ.
